# Dynamic analysis of misalignment modelling with flexible damped coupling in rotor shaft system using classical approach

**DOI:** 10.1038/s41598-026-40743-9

**Published:** 2026-04-06

**Authors:** Harisha Malgi, Saurabh Chandraker, Mohit Aggarwal, Jayanta Kumar Dutt, Kulmani Mehar

**Affiliations:** 1 Department of Mechanical Engineering, NITK Suarthkal, Mangalore, 575025 Karnataka India; 2 Department of Mechanical Engineering, IIT Delhi, Delhi, 110016 India; 3 Manipal Institute of Technology, Manipal Academy of Higher Education, Manipal, India

**Keywords:** Rotor shaft vibration, Segmented disk coupling, Parallel and angular misalignment, Dynamic studies, Engineering, Mathematics and computing, Physics

## Abstract

Misalignment is a common practical problem during the transmission of power from one shaft to another, where the coupling plays a crucial role in determining the dynamic behaviour of the rotor-shaft system. This paper presents a dynamic analysis of two misaligned rotors coupled by a flexible coupling to accommodate both parallel and angular misalignments. A flexible coupling accommodates misalignment to solve this problem. However, in this process, the system dynamics is also influenced by the coupling characteristics, particularly the dissipation characteristics of the coupling, which rotates with the shaft and should be considered as internal damping. Among flexible couplings, segmented disc couplings are widely used in electric motors, engines, cooling towers, and in helicopter tail rotors. This work considers a segmented disc coupling featuring viscoelastic links and aims to derive the mathematical model of the restoring and dissipation behaviour in terms of non-dimensional parameters, represented as matrices. The modelling technique applies to other situations of flexible coupling; however, the stiffness and dissipation behaviour of the coupling should be found appropriately. The use of non-dimensional parameters extends their applicability to similar couplings of any specification. Next, the characteristics derived are used to study the dynamic behaviour of a coupled non-axisymmetric rotor-shaft system, including its stability limits for spin speed and rotor response amplitude due to unbalance, misalignment, and gravity. The 1X and 2X components in the frequency response, along with their interpretation, are illustrated through several examples.

**Supplementary Information:**

The online version contains supplementary material available at 10.1038/s41598-026-40743-9.

## Introduction

Misalignment is a practical condition that arises when power transmission is attempted by coupling a driving shaft to the driven shaft, because the driving and driven shafts are not on the same centerline. There are three basic types of shaft misalignment: axial, parallel, and angular, and a combination of these is typically present in practical situations. Parallel and angular misalignment, or more realistically, a combination thereof, is the second most common fault after unbalance in the rotor shaft system. Misalignment in rotating machinery produces both radial and axial vibrations, often resulting in problems such as looseness, shaft bending, bearing wear, and inertial imbalance. Misalignment produces a fixed directional preload on the shaft and bearings. This directional force is applied to the rotating shaft through the coupling element. Thus, the shaft is forced to rotate in a bent configuration. The magnitude of this preload is a function of the amount of misalignment as well as the type and condition of the coupling^1–3^.

A coupling provides three basic functions: it transmits power, accommodates misalignment to an acceptable degree and compensates for end movement. Some of the most common flexible couplings include mechanically flexible couplings, elastomeric couplings, and metallic membrane couplings, among others. Disc coupling is one of the most used metallic membrane couplings. This provides a wide operating range of speed and torque, has a long operating life, is lightweight, and requires no lubrication. In this process, the dynamic characteristics of the coupling also influence the dynamic behaviour of a coupled rotor-shaft system. However, in the literature surveyed so far, the influence of coupling characteristics, such as stiffness and dissipation, on the dynamic behaviour of the rotor-shaft system has been marginally addressed, despite their importance^1,2^.

Gibbons^2^ proposed a formulation of the misalignment forces of the coupling. Later, many researchers adopted force equations based on the author’s analysis. Sekhar and Prabhu^3^ evaluated the effect of coupling misalignment on vibration response of a rotor by including the forces imposed by the coupling, based on the formulation given by Gibbons^2^. The 2X frequency component was, as usual, found to be significant in identifying the misalignment. In doing so, the authors focused solely on the static misalignment value to model the forces. Therefore, the coupling characteristics did not enter the system matrix, and the system’s modal characteristics remained unaffected. Therefore, the influence of the coupling stiffness and damping on the stability of the rotor shaft system was not reported. It is apparent that the coupling characteristic should be found out, and the equations of motion should include the participation of the coupling characteristic. Lee and Lee^4^ presented a dynamic model of a rotor-ball bearing system that accounts for reaction loads and deformations at the bearing and coupling elements due to misalignment. However, here also, the stiffness and damping of the coupling were not included in the system matrix. Xu and Marangoni^5^ derived misalignment forces based on the kinematics of the Hooke’s joint and concluded that shaft-misalignment produced excitations even at multiples of the spin frequency of the rotor. However, the authors again did not determine the coupling characteristics (stiffness and damping) and therefore did not include them in the equations of motion for the rotor shaft system. Therefore, the aspects of stability and influence of the coupling on the Campbell diagram were also not reported by the authors in^3–5^. Redmond and Al-Hussain^6,7^ commented on the absence of the derivation of stiffness and damping of the coupling presented by Xu and Marngoni^5^. In the work, the authors modelled the stiffness and diagonal damping matrix for a rigid, pinned joint with rotational stiffness and found 1X bending–torsional coupled vibration response for the rotor with parallel misalignment. Sinha et al.^8^ proposed a method for estimating both rotor unbalance and misalignment from a single machine run-down. Experimental validation on a multi-bearing test rig demonstrates the method’s robustness, including its sensitivity to modelling errors. Patel and Darpe^9^ proposed a misalignment model based on experimentally measured data on forces induced by both parallel and angular misalignments. The authors provided only the coupling stiffness matrix. The damping matrix was assumed to be proportional. No attempt was reported to obtain the damping matrix of the coupling. Later, the stiffness matrix was utilised to investigate the system’s response. There was, however, no mention of either the Campbell diagram or the stability of the rotor shaft system. The frequency response showed higher harmonics, and the strength of the 3X frequency component was determined to be an indicator of the dominant misalignment type, either parallel or angular. Although the authors provided a stiffness matrix, there was no mathematical model to derive it, making the exercise coupling-specific and experiment-dependent. Few authors have analysed how multiple faults and uncertain parameters influence the nonlinear vibration response of rotor systems. It combines the Harmonic Balance Method with Polynomial Chaos Expansion (PCE) to efficiently model uncertainty in an asymmetric rotor. The approach accurately predicts vibration amplitudes and resonance behaviour. Results show strong agreement with Monte Carlo simulations at a much lower computational cost^10,11^. Fu et al.^12^ analysed the nonlinear vibration response of rotor systems with parallel and angular misalignments while accounting for realistic uncertainties in fault parameters. Using a finite element model combined with a Legendre collocation–based interval method, it efficiently predicts how uncertainties propagate into harmonic vibration responses. The results show that parallel misalignment mainly affects odd harmonics, angular misalignment mainly affects even harmonics, and the proposed approach provides accurate predictions at much lower computational cost for practical rotor fault analysis. Fu et al.^13^ studied how misalignment and unavoidable parameter uncertainties affect the nonlinear vibrations of a dual-rotor bearing system. Using an efficient interval-based numerical method, it shows that uncertainty in rotor speed ratio and bearing parameters can greatly amplify vibration responses. The results highlight the importance of accounting for uncertainty to ensure reliable design and fault diagnosis in real rotating machinery.

Huang and Peng^14^ investigate the effects of rotational speed, critical torsional stiffness, critical bending stiffness, and angular misalignment on the vibration behaviour of the laminated coupling. Gomes et al.^15^ aim to optimise the topology of a circular disk coupling using the Solid Isotropic Material with Penalisation (SIMP) approach and the Finite Element method to minimise the restitution moment under torque and angular misalignment. Kandil^16^ have also conducted studies on the nonlinear vibration and whirling behaviour of a 16-pole rotor supported by active magnetic bearings, accounting for rotor weight–induced asymmetry. Another attempt to explore how changing the angle of magnetic bearing poles affects the control of an oscillating rotor. It compares two common control methods—radial and Cartesian—and shows they react very differently to angle changes^17^. The author also compares two control methods for active magnetic bearing rotors and shows that using a fixed surplus current can cause severe vibrations, repeated impacts, and unstable behaviour.

By contrast, an adjustable surplus current adapts to rotor motion, greatly reducing impacts and preventing sudden jumps in vibration and is better suited for high-speed practical applications^18,19^.

All these reasons inspired the authors to develop a mathematical model to derive the stiffness and damping matrices for a coupling, particularly the segmented disc type coupling, which is widely used in industry and appears to lack a good model, based on the literature survey conducted so far. The inspiration for the paper stems from the dissipation behaviour in the coupling, which acts as a rotary coupling that rotates with the shaft and causes tangential forces to destabilise the rotor shaft system. The analysis is generic and can be applied to other models of the coupling. To make the model independent of the specification of the segmented disc type coupling, the authors have proposed suitable non-dimensional parameters to represent stiffness and damping matrices. Thus, this work is expected to address the research gap and be very helpful in formulating stiffness and damping matrices, which can be incorporated into the FE model of the coupled-rotor shaft system to predict rotor-dynamic behaviour, including the stability of the rotor-shaft system and rotor response at any point.

## Analysis

The analysis in the section below is presented in three subsections. Firstly, the linear and angular stiffness is derived for the segmented disc coupling, and damping is incorporated by using the operator form of the stiffness to consider the viscoelastic nature of the material of the segmented discs. Secondly, an equivalent operator-based stiffness matrix for the combined rotor-shaft coupling system is obtained in terms of non-dimensional parameters, making it independent of the coupling specification^20–23^. Finally, forces and moments due to the coupling are transferred to the rotor-disc to study the dynamic behaviour of the coupled rotor-shaft system. In this work, the dynamic behaviour of a flexible 4-degree-of-freedom, non-axisymmetric, coupled rotor-shaft system is studied to obtain the Campbell diagram, the stability limit speed of the rotor, and response due to unbalance, gravity, and misalignment.

### Calculation of coupling stiffness and damping

**Fig. 1:**
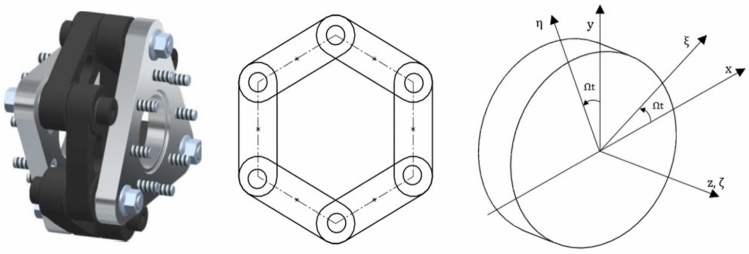
Ship motion group 6 link segmented disc coupling with side view of links and its associated rotated degree of freedom.

In the segmented disc coupling shown in Fig. 1, two hubs are fitted, one on each of the driver and driven shafts. The links (which are actually the segmented discs) are bolted alternatively on the hubs (i.e. one end of the link is bolted on driver hub and the other is bolted on the driven hub), so each hub has bolt holes half the no. of links used if ends of the consecutive links overlap, and bolt holes are equal to the no. of links if consecutive links do not overlap. These links are also known as disc packs, flex elements, flex blades, or flex packs. These flex elements undergo deflections to accommodate misalignment.

#### Coordinate system

A right-handed triad is used to represent the coordinate system, as shown in Fig. 1, where $\mathrm{x}-\mathrm{y}-\mathrm{z}$ represent the axes of the stationary coordinate frame and $\upxi -\upeta -\upzeta$ those of the frame rotating with the shaft. The $\mathrm{z}$ and $\upzeta$ axes are shown coincident at the instant; however, they may move apart.

#### Combined misalignment

**Fig. 2:**
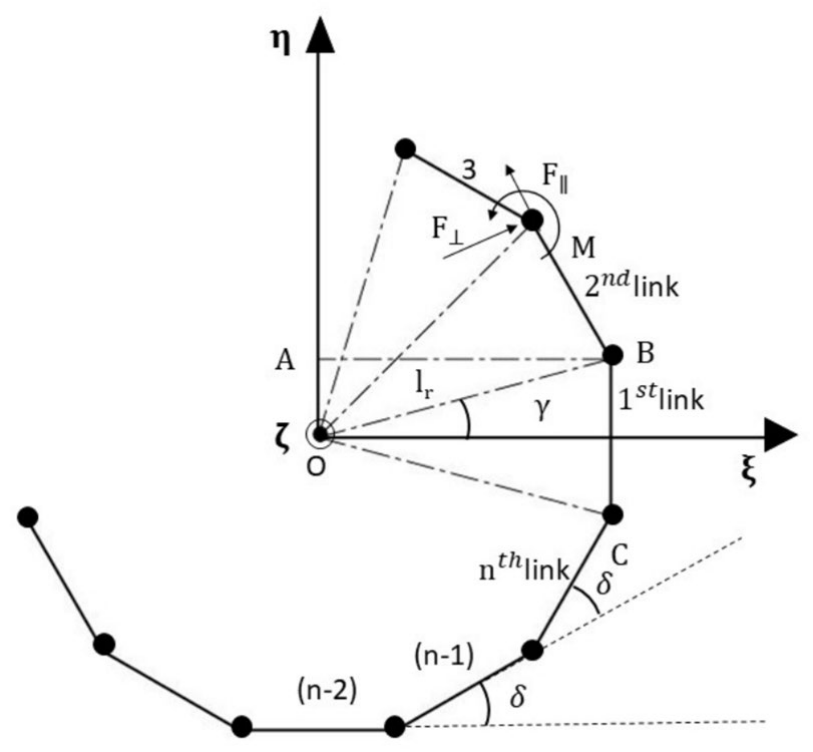
Generalized n-link coupling orientation and angles.

Fig. 2 shows the sketch of a general n-link coupling with alternate bolts attached to the driver and driven ends of the hub, as explained above in section "Calculation of coupling stiffness and damping". In this, an even number of links is assumed to connect the two hubs. The reference frame $\left( {\upxi - \upeta - \upzeta } \right)$ is assumed to be the spin-synchronized frame. The coupling is subjected to a combined misalignment, including both parallel and angular misalignments, such that one end of the coupling is in a generalised deflected position relative to the other. To reach this generalised deflected position, a sequence of transformations is applied to the coupling. Firstly, it is given angular deflections $\upalpha , \upbeta$ as measured in the clockwise sense about $\upxi \text{ and }$ $\upeta$ axes respectively. Then, it is given small instantaneous linear displacements ${\Delta }_{\upxi }$ and ${\Delta }_{\upeta }$ (of one hub of the coupling with respect to its other hub) along $\upxi \text{ and }$ $\upeta$ directions respectively. The small deflection is assumed to ensure the link material’s linear behaviour. Let ${\upxi }_{\mathrm{i}}$, ${\upeta }_{\mathrm{i}}$ and ${\upzeta }_{\mathrm{i}}$ & ${\upxi }_{\mathrm{f}}$, ${\upeta }_{\mathrm{f}}$ and ${\upzeta }_{\mathrm{f}}$ be the initial and final coordinates of any ${\text{r }}^{th}$ link of the coupling before and after deflection, respectively.

The final coordinates are obtained by

1 $$\left[ {\upxi_{{\mathrm{f}}} \;\upeta_{{\mathrm{f}}} \;\upzeta_{{\mathrm{f}}} \;1} \right] = \left[ {\upxi_{{\mathrm{i}}} \;\upeta_{{\mathrm{i}}} \;\upzeta_{{\mathrm{i}}} \;1} \right] * {\mathrm{T}}_{1} * {\mathrm{T}}_{2} * {\mathrm{T}}_{3} ,$$

where

$${\mathrm{T}}_{1} = \left[ {\begin{array}{*{20}c} 1 & 0 & 0 & 0 \\ 0 & {\cos \left( \upalpha \right)} & {\sin \left( \upalpha \right)} & 0 \\ 0 & { - \sin \left( \upalpha \right)} & {\cos \left( \upalpha \right)} & 0 \\ 0 & 0 & 0 & 1 \\ \end{array} } \right];\;{\mathrm{T}}_{2} = \left[ {\begin{array}{*{20}c} {\cos \left( \upbeta \right)} & 0 & { - \sin \left( \upbeta \right)} & 0 \\ 0 & 1 & 0 & 0 \\ {\sin \left( \upbeta \right)} & 0 & {\cos \left( \upbeta \right)} & 0 \\ 0 & 0 & 0 & 1 \\ \end{array} } \right];\;{\mathrm{and}}\;T_{3} = \left[ {\begin{array}{*{20}c} 1 & 0 & 0 & 0 \\ 0 & 1 & 0 & 0 \\ 0 & 0 & 1 & 0 \\ {\Delta \upxi } & {\Delta \upeta } & 0 & 1 \\ \end{array} } \right]$$

The total deflections along $\upxi$, $\upeta$ and $\upzeta$ axes are obtained as

2 $$\Delta_{\upxi \mathrm{t}} = \upxi_{\mathrm{f}} - \upxi_{\mathrm{i}}$$

3 $$\Delta_{{\upeta {\mathrm{t}}}} = \upeta_{{\mathrm{f}}} - \upeta_{{\mathrm{i}}}$$

4 $$\Delta_{{\upzeta {\mathrm{t}}}} = \upzeta_{{\mathrm{f}}} - \upzeta_{{\mathrm{i}}}$$

**Fig. 3:**
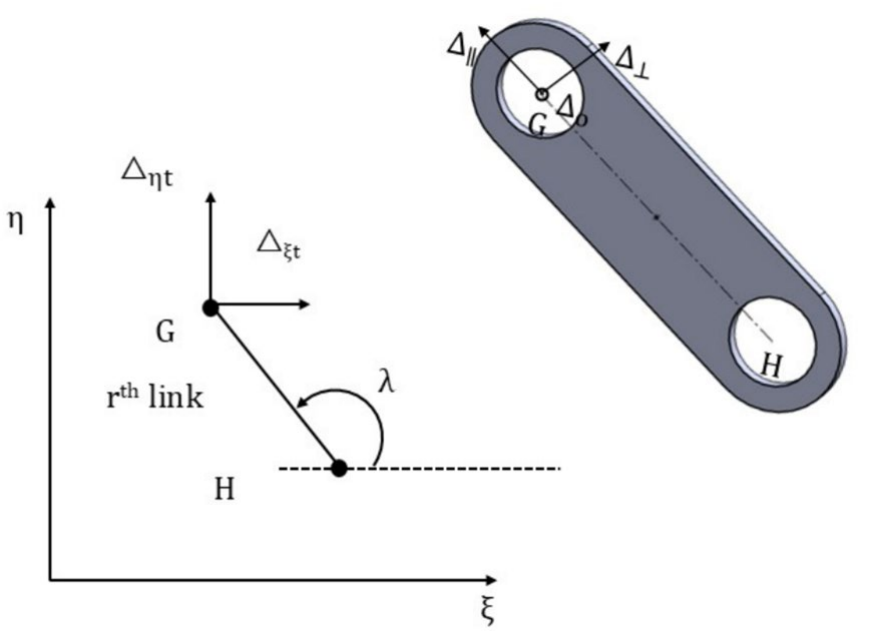
Deflections in the r^th^ link.

For any closed regular polygon of side $\mathrm{n}$, the exterior angle between two consecutive links is $\updelta$ is equal to $\frac{2\uppi }{\mathrm{n}}$ radian. In Fig. 3, let $\uplambda$ be the angle made by the r^th^ link with $\upxi$ axis where, $\uplambda =\updelta \left(\mathrm{r}-1\right).$ Let ${\Delta }_{||}$, ${\Delta }_{\perp }$ and ${\Delta }_{\mathrm{o}}$ be the deflections of the head of the link (link end G) with respect to the base (link end H), parallel, perpendicular and out of the plane to the r^th^ link, respectively, which are given by

5 $$\left[ {\begin{array}{*{20}c} {{\Delta }_{||} } & {{\Delta }_{ \bot } } & {{\Delta }_{\mathrm{o}} } & 1 \\ \end{array} } \right] = { }\left[ {\begin{array}{*{20}c} {{\Delta }_{{{\upxi \mathrm{t}}}} } & {{\Delta }_{{{\upeta \mathrm{t}}}} } & {{\Delta }_{{{\upzeta \mathrm{t}}}} } & 1 \\ \end{array} } \right]{\mathrm{*R}}$$

Here, R is rotation matrix given by

$$\left[\begin{array}{cccc}\mathrm{cos}\left(\uplambda \right)& \mathrm{sin}\left(\uplambda \right)& 0& 0\\ -\mathrm{sin}\left(\uplambda \right)& \mathrm{cos}\left(\uplambda \right)& 0& 0\\ 0& 0& 1& 0\\ 0& 0& 0& 1\end{array}\right]$$

6 $$\Delta_{||} = \cos \left( \uplambda \right)\left( {\Delta_{\upxi } - \upxi_{{\mathrm{i}}} + \upxi_{{\mathrm{i}}} \cos \left( \upbeta \right) + \upeta_{{\mathrm{i}}} \sin \left( \upalpha \right)\sin \left( \upbeta \right)} \right) - \left( {\Delta_{\upeta } - \upeta_{{\mathrm{i}}} + \upeta_{{\mathrm{i}}} \cos \left( \upalpha \right)} \right)\sin \left( \uplambda \right)$$

For small values of α and β (as angular misalignment is approx. 1-4 degrees), the above equation transforms as

7 $${\Delta }_{||} =\mathrm{cos}\left(\uplambda \right){\Delta }_{\upxi }-{\Delta }_{\upeta }\mathrm{sin}\left(\uplambda \right)$$

Similarly,

8 $${\Delta }_{\perp }={\Delta }_{\upeta }\mathrm{cos}\left(\uplambda \right)+{\Delta }_{\upxi }\mathrm{sin}\left(\uplambda \right)$$

9 $$\Delta_{\mathrm{o}} = \upeta_{{\mathrm{i}}} \upalpha - \upxi_{{\mathrm{i}}} \upbeta$$

**Fig. 4:**
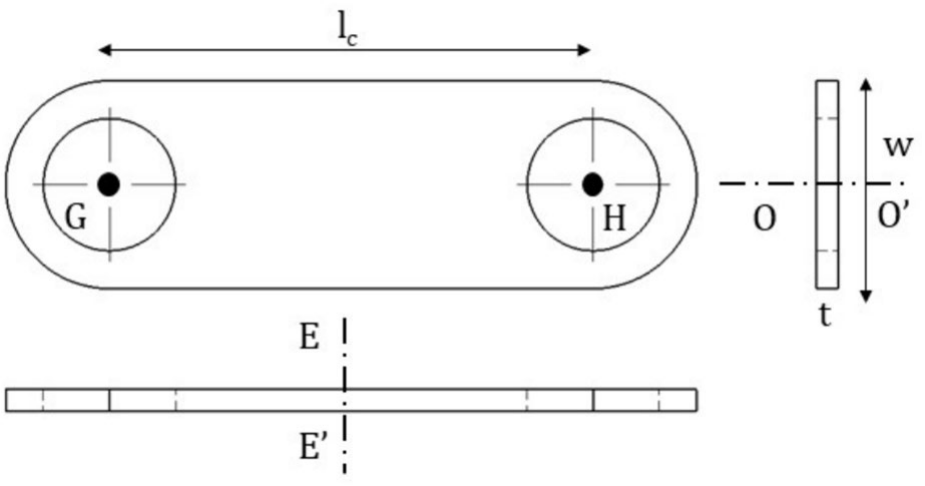
Single link of coupling.

**Fig. 5:**
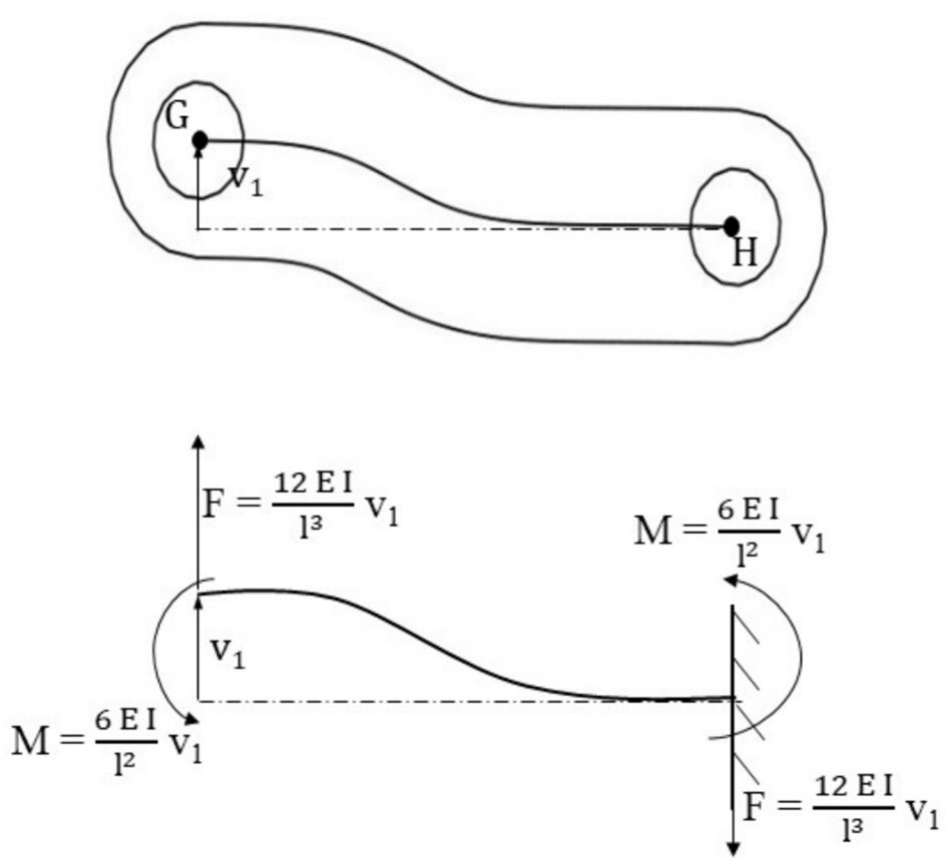
Forces and moments in single link.

The forces and moments due to ${\Delta }_{||}$ and ${\Delta }_{\perp }$ are calculated as: Each link described above acts like a beam element, the ends of which are rigidly fixed, to ensure that they are aligned perfectly with the hub at the point of fixation. This ensures that the end slopes remain zero despite deflection, as shown in Figs. 4 and 5.

**Fig. 6:**
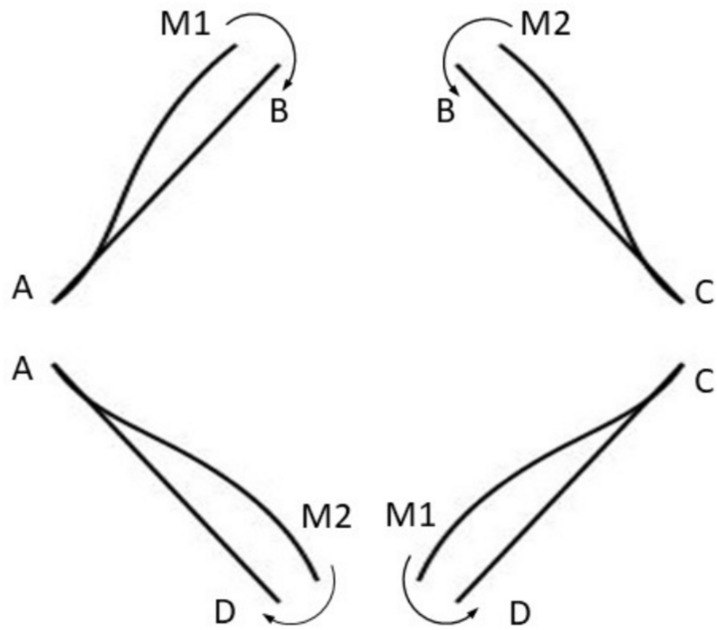
Moments after deflection in a 4-link coupling.

Since the links are alternately bolted to the driver and driven hubs, the net moment is zero, as shown in Fig. 6 below, for a 4-link coupling.

In general, the forces parallel and perpendicular to r^th^ link, and ${\mathrm{F}}_{\perp }$ respectively, are given by

10 $${\mathrm{F}}_{\parallel } = {\mathrm{k}}_{1} \Delta_{\parallel } \,{\mathrm{and}}\,{\mathrm{F}}_{ \bot } = {\mathrm{k}}_{2} {\kern 1pt} \Delta_{ \bot }$$

In the above, the expressions of stiffness ${\mathrm{k}}_{1}$ and ${\mathrm{k}}_{2}$ are given by ${\mathrm{k}}_{1}={\mathrm{AE}}_{\mathrm{c}}/\mathrm{l}$ and ${\mathrm{k}}_{2}=12{\mathrm{E}}_{\mathrm{c}}\mathrm{I}/{\mathrm{l}}_{\mathrm{c}}^{3}\mathrm{l}$, ${\mathrm{l}}_{\mathrm{c}}$ is the length of the link, E_c_ is the modulus of elasticity of coupling material, A is the area of cross section = w t, w is the width, and t is the thickness, I is the area moment of inertia of the link (G-H) about neutral axis OO’ of the link as shown in Fig. 4. Therefore, the component of forces along ξ and $\upeta$ directions at any instant of time are given by

11 $$\left( {\begin{array}{*{20}c} {{\mathrm{F}}_{{\upxi }} } \\ {{\mathrm{F}}_{{\upeta }} } \\ \end{array} } \right) = {\mathrm{R}}^{{\mathrm{T}}} *\left( {\begin{array}{*{20}c} {{\mathrm{F}}_{\parallel } } \\ {{\mathrm{F}}_{ \bot } } \\ \end{array} } \right)$$

R^T^ is the transpose of the rotation matrix R as given above.

Summing all such forces for all the links. The total force acting on the hub ${\mathrm{F}}_{\upxi }^{\mathrm{t}}$ and ${\mathrm{F}}_{\upeta }^{\mathrm{t}}$ in the $\upxi$ and $\upeta$ directions, respectively, are obtained as

12 $${\mathrm{F}}_{\upxi }^{{\mathrm{t}}} = \sum\nolimits_{{{\mathrm{r}} = 1}}^{{\mathrm{n}}} {{\mathrm{F}}_{\upxi } } \,\;{\mathrm{and}}\,\;\mathrm{F}_{\upeta }^{{\mathrm{t}}} = \sum\nolimits_{{{\mathrm{r}} = 1}}^{{\mathrm{n}}} {{\mathrm{F}}_{\eta } }$$

13 $${\mathrm{F}}_{\upxi }^{{\mathrm{t}}} = {\mathrm{k}}\Delta_{\upxi } \;{\mathrm{and}}\,\,{\mathrm{F}}_{\upeta }^{{\mathrm{t}}} = {\mathrm{k}}\Delta_{\upeta }$$

Here, k denotes the equivalent lateral stiffness of the coupling. Variation of k with the number of links is shown in Tables 1.

**Table 1:** Variation of stiffness k with no. of links n as calculated in parallel misalignment.

No. of links	Stiffness (k)
4	2 $\left({\mathrm{k}}_{1}+{\mathrm{k}}_{2}\right)$
6	3 $\left({\mathrm{k}}_{1}+{\mathrm{k}}_{2}\right)$
8	4 $\left({\mathrm{k}}_{1}+{\mathrm{k}}_{2}\right)$
n	0.5n $\left({\mathrm{k}}_{1}+{\mathrm{k}}_{2}\right)$

Here $${\mathrm{k}}_{1}=\frac{{\mathrm{AE}}_{\mathrm{C}}}{\mathrm{l}}$$ and $${\mathrm{k}}_{2}=\frac{12{\mathrm{E}}_{\mathrm{C}}\mathrm{l}}{{\mathrm{l}}_{\mathrm{C}}^{3}}$$

If similar links are used as the sides of the regular polygon to connect the hubs, then the coupling stiffness becomes the same in both $\upxi$ & $\upeta$ directions. They will certainly be different if the links are dissimilar. Therefore, using similar links to form the sides of a regular polygon produces isotropic stiffness in the coupling.

Forces and moments due to ${\Delta }_{\mathrm{o}}$ are calculated as: The orientation of n link coupling is shown again to define angular misalignment in Fig. 2. Here, 1, 2, 3…. n-1, n represents the location of the 1st, 2nd, 3rd ……. nth bolt, respectively, as seen in the end view; a similar view was given in Fig. 3.

Here $\upgamma$ is the half interior angle given by, $\upgamma =\frac{\uppi }{\mathrm{n}}$ radian. Let ${\mathrm{l}}_{\mathrm{c}}$ be the length of a single link (BC) of the coupling, then ${\mathrm{l}}_{\mathrm{r}}=\frac{{\mathrm{l}}_{\mathrm{c}}}{2\mathrm{sin}\left(\upgamma \right)}$.

Eq. 9 gives the value of the deflection out of the plane, i.e ${\Delta }_{\mathrm{o}}$ which depends on the angular deflections $\upalpha$ and $\upbeta$ and the initial positions of the bolts ${\upxi }_{\mathrm{i}}$ and ${\upeta }_{\mathrm{i}}$ Thus, Eq. 9 can be rewritten as deflection due to α and β separately as $\Delta_{{\mathrm{o}}} = \Delta_{{{\mathrm{o}}\upalpha }} + \Delta_{{{\mathrm{o}}\upbeta }}$ where $\Delta_{{{\mathrm{o}}\upalpha }} = \upeta_{{\mathrm{i}}} \upalpha$ and $\Delta_{{{\mathrm{o}}\upbeta }} = \xi_{{\mathrm{i}}} \upbeta$.

**Fig. 7:**
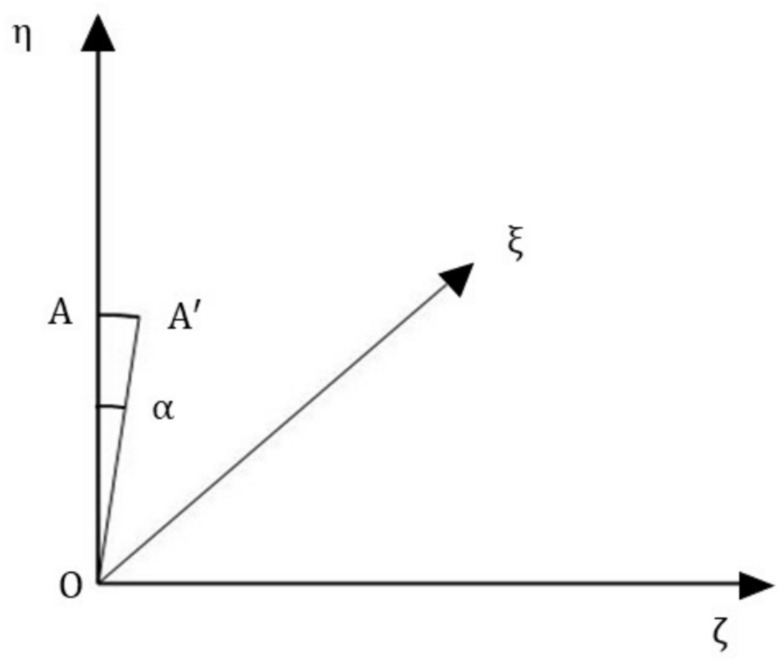
Deflection in 1^st^ link due to ${\boldsymbol{\upalpha}}$.

Fig. 7 explains the angular deflection of the length OA by an angle $\alpha$ considered clockwise about the $\upxi$ axis. In this process, OA moves to OA^’^. Forces and Moments produced due to angular displacement $\upalpha$ are calculated as follows.

The component of the OA in the $\upzeta$ direction (out of the plane of the coupling) due to angular deflection $\upalpha$ is Δ_oα._

For bolt 1, ${\upxi }_{\mathrm{i}}={\mathrm{l}}_{\mathrm{r}}\mathrm{sin}\left(\upgamma \right)$ and ${\upeta }_{\mathrm{i}}={\mathrm{l}}_{\mathrm{r}}\mathrm{cos}\left(\upgamma \right)$ Therefore, the force outside the plane (in the direction of $\upzeta$), $\mathrm{F}_{\mathrm{o}\upalpha }$ is calculated as

14 $${\mathrm{F}}_{{{\mathrm{o}}\alpha }} = {\mathrm{k}}_{3} \Delta_{{{\mathrm{o}}\alpha }}$$

where,${\mathrm{k}}_{3}$ is $\frac{12{\mathrm{E}}_{\mathrm{c}}{\mathrm{I}}_{1}}{{\mathrm{l}}^{3}}$, ${\mathrm{I}}_{1}$ is the area moment of inertia about the neutral axis $\mathrm{EE}$ (Fig. 4) of the link.

Moments due to this force about $\upxi { }$ and $\upeta$ axes are given as

15 $${\mathrm{M}}_{\upxi \upalpha } = {\mathrm{F}}_{{{\mathrm{o}}\upalpha }} {\mathrm{OA}} = \upalpha {\mathrm{k}}_{3} {\mathrm{l}}_{{\mathrm{r}}}^{2} \sin^{2} \left( \upgamma \right)$$

16 $${\mathrm{M}}_{\upeta \upalpha } = - {\mathrm{F}}_{{{\mathrm{o}}\upalpha }} {\mathrm{OB}} = - \upalpha {\mathrm{k}}_{3} {\mathrm{l}}_{{\mathrm{r}}}^{2} \sin \left( \upgamma \right)\cos \left( \upgamma \right) = - \frac{{\upalpha {\mathrm{k}}_{3} {\mathrm{l}}_{{\mathrm{r}}}^{2} \sin \left( {2\upgamma } \right)}}{2}$$

Since alternate bolts are present on the same hub, the angle made with the $\upxi$ axis by different bolts is given by $\upgamma$, 5 $\upgamma$, 9 $\upgamma$…. etc. This means that the ${\mathrm{r}}^{\mathrm{th}}$ bolt will make an angle of $\left(4\mathrm{r}-3\right)$ $\upgamma$ with the $\upxi -$ axis. The total moment is the summation of the moments due to all links.

The summation of all the forces comes out to be zero as half of the coupling goes inside, while the other half comes out, if the number of links is chosen even.

17 $$\sum {\mathrm{M}}_{\upxi \upalpha } = \upalpha {\mathrm{k}}_{3} {\mathrm{l}}_{{\mathrm{r}}}^{2} \left( {\sin^{2} \left( \upgamma \right) + \sin^{2} \left( {5\upgamma } \right) + \sin^{2} \left( {9\upgamma } \right)..} \right) = \upalpha {\mathrm{k}}_{{\mathrm{b}}}$$

18 $$\sum {\mathrm{M}}_{\upeta \upalpha } = - \upalpha {\mathrm{k}}_{3} {\mathrm{l}}_{{\mathrm{r}}}^{2} \frac{{\left( {\sin \left( {2\upgamma } \right) + \sin \left( {2\left( {5\upgamma } \right)} \right) + \sin \left( {2\left( {9\upgamma } \right)} \right)..} \right)}}{2} = - \upalpha {\mathrm{k}}_{{{\mathrm{bc}}}}$$

Similarly, moments due to angular deflection $\upbeta$ about $\upeta$ axis is given by

19 $$\sum {\mathrm{M}}_{\upxi \upbeta } = - \upbeta {\mathrm{k}}_{3} {\mathrm{l}}_{{\mathrm{r}}}^{2} \frac{{\sin \left( {2\upgamma } \right)}}{2} \,\,{\mathrm{and}}\,\, {\mathrm{M}}_{\upeta \upbeta } = \upbeta {\mathrm{k}}_{3} {\mathrm{l}}_{{\mathrm{r}}}^{2} \cos^{2} \left( \upgamma \right)$$

20 $$\sum {\mathrm{M}}_{\upxi \upbeta } = { } - { }\upbeta {\text{ k}}_{3} {\text{ l}}_{{\mathrm{r}}}^{2} { }\frac{{\left( {\sin \left( {2\upgamma } \right) + \sin \left( {2\left( {5\upgamma } \right)} \right) + \sin \left( {2\left( {9\upgamma } \right)} \right)..} \right)}}{2}{ } = { } - { }\upbeta {\text{ k}}_{{{\mathrm{bc}}}}$$

21 $$\sum {\mathrm{M}}_{\upeta \upbeta } = { }\upbeta {\text{ k}}_{3} {\text{ l}}_{{\mathrm{r}}}^{2} \left( {\cos^{2} \left( \upgamma \right) + \cos^{2} \left( {2\left( {5\upgamma } \right)} \right) + \cos^{2} \left( {2\left( {9\upgamma } \right)} \right)} \right) = { }\upbeta {\text{ k}}_{{\mathrm{b}}}$$

Here, ${\mathrm{k}}_{\mathrm{b}}$ and ${\mathrm{k}}_{\mathrm{bc}}$ denotes the equivalent bending stiffness and cross-coupling bending stiffness of the coupling, respectively. Values of ${\mathrm{k}}_{\mathrm{b}}$ and ${\mathrm{k}}_{\mathrm{bc}}$ For a different number of links, see in Table 2.

**Table 2:** Variation of stiffness k_b_ and k_bc_ with no. of links n as calculated in angular misalignment.

**No. of links**	**k**_**b**_	**k**_**bc**_
4	k_3_ l_r_^2^	k_3_ l_r_^2^
6	1.5 k_3_ l_r_^2^	0
8	2 k_3_ l_r_^2^	0
10	2.5 k_3_ l_r_^2^	0

From here, we get equivalent total moments due to $\upalpha$ and $\upbeta$ as

22 $${\mathrm{M}}_{\upxi }^{{\mathrm{t}}} = { }\upalpha {\mathrm{k}}_{{\mathrm{t}}} { } - { }\upbeta {\text{ k}}_{{{\mathrm{bc}}}} \,{\mathrm{and}}\;{\mathrm{M}}_{\upeta }^{{\mathrm{t}}} = { }\upbeta {\text{ k}}_{{\mathrm{t}}} { } - \upalpha {\text{ k}}_{{{\mathrm{bc}}}}$$

For a number of links greater than six ${\mathrm{k}}_{\mathrm{bc}}$ is zero shown in Table 2, thus

23 $${\mathrm{M}}_{\upxi }^{{\mathrm{t}}} = \upalpha {\mathrm{k}}_{{\mathrm{b}}} \;{\mathrm{and}}\;{\mathrm{M}}_{\upeta }^{{\mathrm{t}}} = \upbeta {\mathrm{k}}_{{\mathrm{b}}}$$

Here ${\mathrm{k}}_{3}=\frac{12{\mathrm{E}}_{\mathrm{C}}{\mathrm{I}}_{1}}{{\mathrm{l}}_{3}}$, I_1_ is the area moment of inertia about the neutral axis EE’ (Fig. 4) of the link.

Thus, the equivalent coupling stiffness operator for a number of links greater than 6 is given as

$$\left[\begin{array}{cccc}\mathrm{k}& 0& 0& 0\\ 0& \mathrm{k}& 0& 0\\ 0& 0& {\mathrm{k}}_{\mathrm{t}}& 0\\ 0& 0& 0& {\mathrm{k}}_{\mathrm{t}}\end{array}\right]$$

Since an even number of links, or in that sense, the number of bolts is even, so due to any angular movement between the two hubs, about their diameter, half of the bolts will deflect in one direction and the other half will go to the opposite direction from a hypothetical mean nominal plane when the hubs are parallel. Under this situation, the use of similar links arranged as sides of a regular polygon is shown in Fig. 2. Equal and opposite forces will result for the pair of diametrically opposite bolts, causing no net force. However, there will be a net moment. This also means that if the links are dissimilar or the number of links is uneven, such a conclusion will not be accurate. Possibly for this reason, any coupling with an odd no. of links is never used.

### Calculating the combined coupling rotor-shaft system stiffness matrix

If misalignment is not present in the system, forces and moments due to coupling still occur, as the deflection at the end is restricted by the coupling stiffness. Thus, the system is modified, as the end conditions are determined by the coupling. Thus, it is necessary to find the modified stiffness matrix rather than the stiffness and dissipation matrices.

**Fig. 8:**
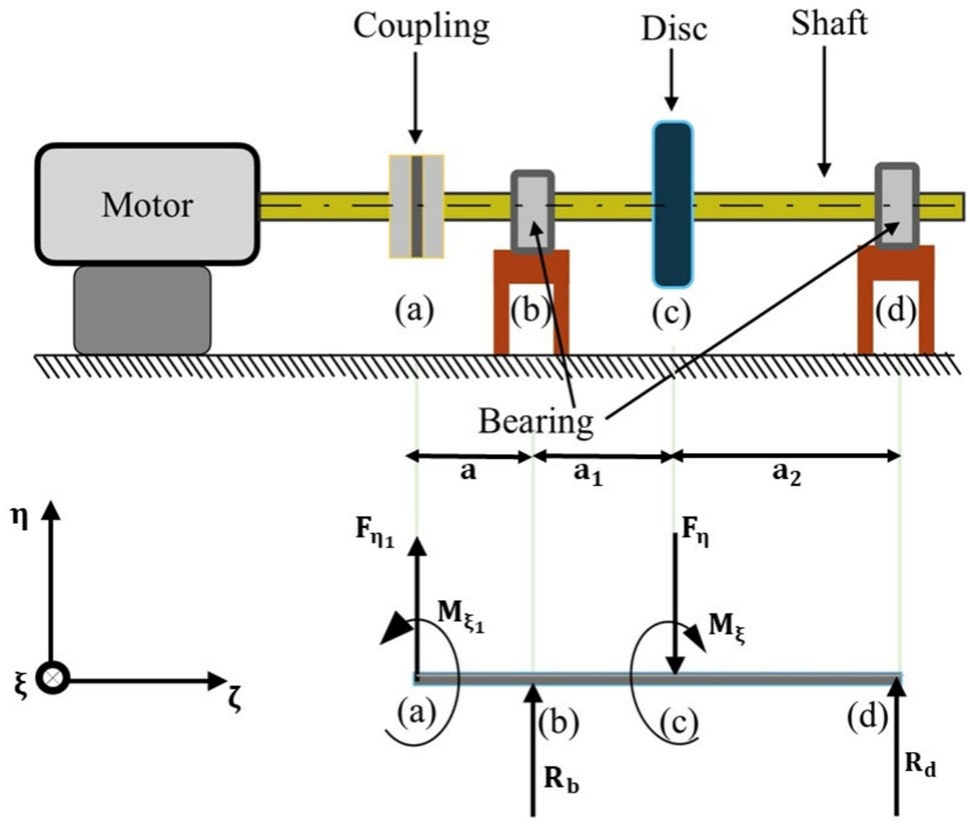
Rotor-Coupling system with rigid pedestals.

The linear and angular stiffness obtained from the above analysis will now be used to determine the equivalent stiffness of the rotor-coupling system. The system is shown in Fig. 8, where a short rigid input shaft is coupled to a relatively long, flexible output shaft. In this system, all pedestals are assumed rigid, and no attempt is made to account for support stiffness and damping to investigate the influence of coupling characteristics on the dynamic behaviour of the coupled-rotor shaft system.

Calculating reactions and the bending moment in the shaft in $\upeta -\upzeta$ plane.

Fig.8 shows the forces and moments on the shaft and the coupling in the $\upeta -\upzeta$ plane. Let ${\mathrm{F}}_{\upeta 1}$, ${\mathrm{M}}_{\upxi 1}$ and ${\mathrm{F}}_{\upeta }$, ${\mathrm{M}}_{\upxi }$ be the forces and moments at coupling and rotor location, respectively, and R_1_ and R_2_ be the reactions at bearing end B and C, respectively. The figure above shows the locations of the bearings, coupling, and supports. Whereas ${\mathrm{F}}_{\upeta }$ and ${\mathrm{M}}_{\upxi }$ are supposed to be known quantities, all the rest are unknown. The section below attempts to derive other unknown quantities in terms of ${\mathrm{F}}_{\upeta }$ and ${\mathrm{M}}_{\upxi }$.

By balancing all the forces and moments

24 $${\mathrm{R}}_{\mathrm{b}}=\frac{-{\mathrm{F}}_{{\upeta }_{1}}\left(\mathrm{a}+{\mathrm{a}}_{1}+{\mathrm{a}}_{2}\right)+{{\mathrm{a}}_{2}\mathrm{F}}_{\upeta }-{\mathrm{M}}_{{\upxi }_{1}}-{\mathrm{M}}_{\upxi }}{\left({\mathrm{a}}_{1}+{\mathrm{a}}_{2}\right)}$$

25 $${\mathrm{R}}_{\mathrm{d}}=\frac{{\mathrm{aF}}_{{\upeta}_{1}}+{\mathrm{a}}_{1}{\mathrm{F}}_{\upeta}+{{\mathrm{M}}_{\upxi}+\mathrm{M}}_{{\upxi}_{1}}}{\left({\mathrm{a}}_{1}+{\mathrm{a}}_{2}\right)}$$

Bending moments in the section $\mathrm{ab}$, $\mathrm{bc}$, and $\mathrm{cd}$ of the shaft, M_ab_, ${\mathrm{M}}_{\mathrm{bc}}$ and ${\mathrm{M}}_{\mathrm{cd}}$ respectively, are written in the equations (26) to (28), where $\upzeta$ is the distance from the leftmost end of each section in the ζ direction.

26 $${\mathrm{M}}_{\mathrm{ab}}={{\mathrm{M}}_{{\upxi }_{1}}+\upzeta \mathrm{F}}_{{\upeta }_{1}}$$

27 $${\mathrm{M}}_{\mathrm{bc}}={\mathrm{M}}_{{\upxi }_{1}}+\left(\upzeta +\mathrm{a}\right){\mathrm{F}}_{{\upeta }_{1}}+\upzeta {\mathrm{R}}_{\mathrm{b}}$$

28 $${\mathrm{M}}_{\mathrm{cd}}={{\mathrm{M}}_{{\upxi }_{1}}+\mathrm{M}}_{\upxi }-\upzeta {\mathrm{F}}_{\upeta }+{(\upzeta +\mathrm{a}+{\mathrm{a}}_{1})\mathrm{F}}_{{\upeta }_{1}}+{\left(\upzeta +{\mathrm{a}}_{1}\right)\mathrm{R}}_{\mathrm{a}}$$

Using Castigliano’s theorem, the total internal energy in the shaft is given as

$$\text{U }= {\int }_{0}^{\mathrm{l}}\frac{{\mathrm{M}}^{2}}{2\mathrm{EI}}d\upzeta ={\int }_{0}^{\mathrm{a}}\frac{{{\mathrm{M}}_{\mathrm{ab}}}^{2}}{2\mathrm{EI}}d\upzeta +{\int }_{0}^{\mathrm{a}1}\frac{{{\mathrm{M}}_{\mathrm{bc}}}^{2}}{2\mathrm{EI}}d\upzeta +{\int }_{0}^{\mathrm{a}2}\frac{{{\mathrm{M}}_{\mathrm{cd}}}^{2}}{2\mathrm{EI}}d\upzeta$$

Let ${\mathrm{v}}_{{\upeta {\mathrm{d}}}}$ be deflection along $\upeta$ direction, $\upalpha_{{\upxi {\mathrm{d}}}}$ angular deflection about $\upxi$ axis at the location of the disc and ${\mathrm{v}}_{{\upeta {\mathrm{c}}}}$ be deflection along $\upeta$ direction and $\upalpha_{\upxi c}$ angular deflection about $\upxi$ axis at coupling. Thus

29 $${\mathrm{v}}_{\upeta \mathrm{d}}=\frac{\partial \mathrm{U}}{\partial {\mathrm{F}}_{\upeta }}={\mathrm{C}}_{11}{\mathrm{F}}_{\upeta }+{\mathrm{C}}_{12}{\mathrm{M}}_{\upxi }+{\mathrm{C}}_{13}{\mathrm{F}}_{\upeta 1}+{\mathrm{C}}_{14}{\mathrm{M}}_{\upxi 1}$$

30 $${\upalpha }_{\upxi \mathrm{d}}=\frac{\partial \mathrm{U}}{\partial {\mathrm{M}}_{\upxi }}={\mathrm{C}}_{21}{\mathrm{F}}_{\upeta }+{\mathrm{C}}_{22}{\mathrm{M}}_{\upxi }+{\mathrm{C}}_{23}{\mathrm{F}}_{\upeta 1}+{\mathrm{C}}_{24}{\mathrm{M}}_{\upxi 1}$$

31 $${\mathrm{v}}_{\upeta \mathrm{c}}=\frac{\partial \mathrm{U}}{\partial {\mathrm{F}}_{\upeta 1}}={\mathrm{C}}_{31}{\mathrm{F}}_{\upeta }+{\mathrm{C}}_{32}{\mathrm{M}}_{\upxi }+{\mathrm{C}}_{33}{\mathrm{F}}_{\upeta 1}+{\mathrm{C}}_{34}{\mathrm{M}}_{\upxi 1}$$

32 $$\upalpha_{{\upxi {\mathrm{c}}}} = \frac{{\partial {\mathrm{U}}}}{{\partial {\mathrm{M}}_{\upxi 1} }} = {\mathrm{C}}_{41} {\mathrm{F}}_{\upeta } + {\mathrm{C}}_{42} {\mathrm{M}}_{\upxi } + {\mathrm{C}}_{43} {\mathrm{F}}_{\upeta 1} + {\mathrm{C}}_{44} {\mathrm{M}}_{\upxi 1}$$

In the above expressions ${\mathrm{C}}_{\mathrm{ij}}\left(i=j=1\, to\, 4\right)$ is the indicial notation of the influence coefficients. At the coupling, ${\mathrm{F}}_{\upeta 1} = - {\mathrm{kv}}_{{\upeta {\mathrm{c}}}}$ and ${\mathrm{M}}_{\upxi 1} = - {\mathrm{k}}_{{\mathrm{b}}} \upalpha_{{\upxi {\mathrm{c}}}}$ (From Eq. (13) and Eq. (23)). Putting the expressions of ${\mathrm{v}}_{{\upeta {\mathrm{c}}}}$ and $\upalpha_{{\upxi {\mathrm{c}}}}$ from above in Eqs (31) and (32), ${\mathrm{F}}_{\upeta 1}$ and ${\mathrm{M}}_{\upxi 1}$ may be calculated in terms of ${\mathrm{F}}_{\upeta }$ and ${\mathrm{M}}_{\upxi }$. Finally, putting the expressions of ${\mathrm{F}}_{\upeta 1}$ and ${\mathrm{M}}_{\upxi 1}$ in Eq. (29) and (30), ${\mathrm{v}}_{{\upeta {\mathrm{d}}}}$ and $\upalpha_{{\upxi {\mathrm{d}}}}$ are obtained as

$$\left[ {\begin{array}{*{20}c} {{\mathrm{v}}_{{\upeta {\mathrm{d}}}} } \\ {\upalpha_{{\upxi {\mathrm{d}}}} } \\ \end{array} } \right] = \left[ { \begin{array}{*{20}c} {{\mathrm{C}}^{\prime}_{11} } & {{\mathrm{C}}^{\prime}_{12} } \\ {{\mathrm{C}}^{\prime}_{21} } & {{\mathrm{C}}^{\prime}_{22} } \\ \end{array} } \right]\left[ {\begin{array}{*{20}c} {{\mathrm{F}}_{\upeta } } \\ {{\mathrm{M}}_{\upxi } } \\ \end{array} } \right]$$

This gives

$$\left[ {\begin{array}{*{20}c} {{\mathrm{F}}_{\upeta } } \\ {{\mathrm{M}}_{\upxi } } \\ \end{array} } \right] = \left[ {{ }\begin{array}{*{20}c} {{\mathrm{k}}_{{\upeta {\mathrm{t}}}} } & { - {\text{ k}}_{{\upeta {\mathrm{c}}}} } \\ { - {\text{ k}}_{{\upeta {\mathrm{c}}}} } & {{\text{ k}}_{{\upeta {\mathrm{r}}}} } \\ \end{array} { }} \right]\left[ {\begin{array}{*{20}c} {{\mathrm{v}}_{{\upeta {\mathrm{d}}}} } \\ {\upalpha_{{\upxi {\mathrm{d}}}} } \\ \end{array} } \right]$$

Where, the stiffness matrix

33 $$\left[ {\begin{array}{*{20}c} {{\mathrm{k}}_{{{\upeta t}}} } & { - {\text{ k}}_{{{\upeta c}}} } \\ { - {\text{ k}}_{{{\upeta c}}} } & {{\text{ k}}_{{{\upeta r}}} } \\ \end{array} { }} \right] = \left[ {{ }\begin{array}{*{20}c} {{\mathrm{C}}^{\prime }_{11} } & {{\mathrm{C}}^{\prime }_{12} } \\ {{\mathrm{C}}^{\prime }_{21} } & {{\mathrm{C}}^{\prime }_{22} } \\ \end{array} { }} \right]^{ - 1}$$

Similarly, carrying out the analysis in $\upxi -\upzeta$ plane

34 $$\left[ {\begin{array}{*{20}c} {{\mathrm{k}}_{{\upxi {\mathrm{t}}}} } & { - {\mathrm{k}}_{{\upxi {\mathrm{c}}}} } \\ { - {\mathrm{k}}_{{\upxi {\mathrm{c}}}} } & { {\mathrm{k}}_{{\upxi {\mathrm{r}}}} } \\ \end{array} } \right] = \left[ { \begin{array}{*{20}c} {{\mathrm{C}}^{\prime }_{31} } & {{\mathrm{C}}^{\prime }_{32} } \\ {{\mathrm{C}}^{\prime }_{41} } & {{\mathrm{C}}^{\prime }_{42} } \\ \end{array} } \right]^{ - 1}$$

The elements in the stiffness matrix and their non-dimensionalized form are given in the supplementary material (Appendix 1 (a)).

### Torque transmission

**Fig. 9:**
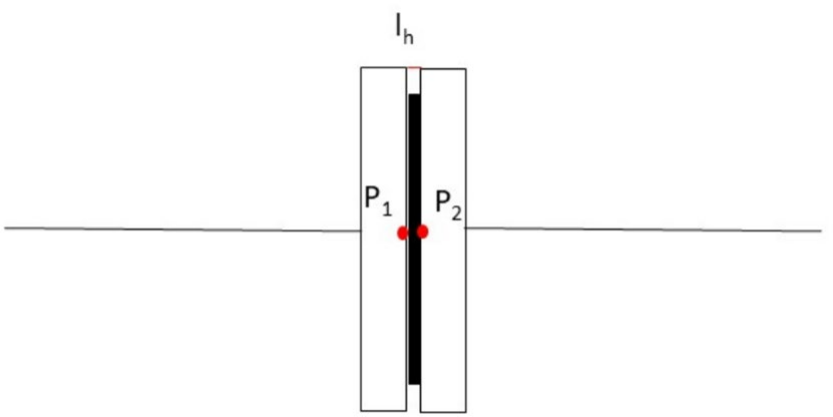
Coupling in a perfectly aligned condition.

The initially aligned condition of the coupling is shown in Fig. 9, where P_1_ and P_2_ are the centres of the two coupling ends joining the driver and driven shafts. Let the initial length between the centre of the driver and driven hubs, i.e., the distance between P_1_ to P_2,_ be l_h_.

#### Torque with parallel misalignment

**Fig. 10:**
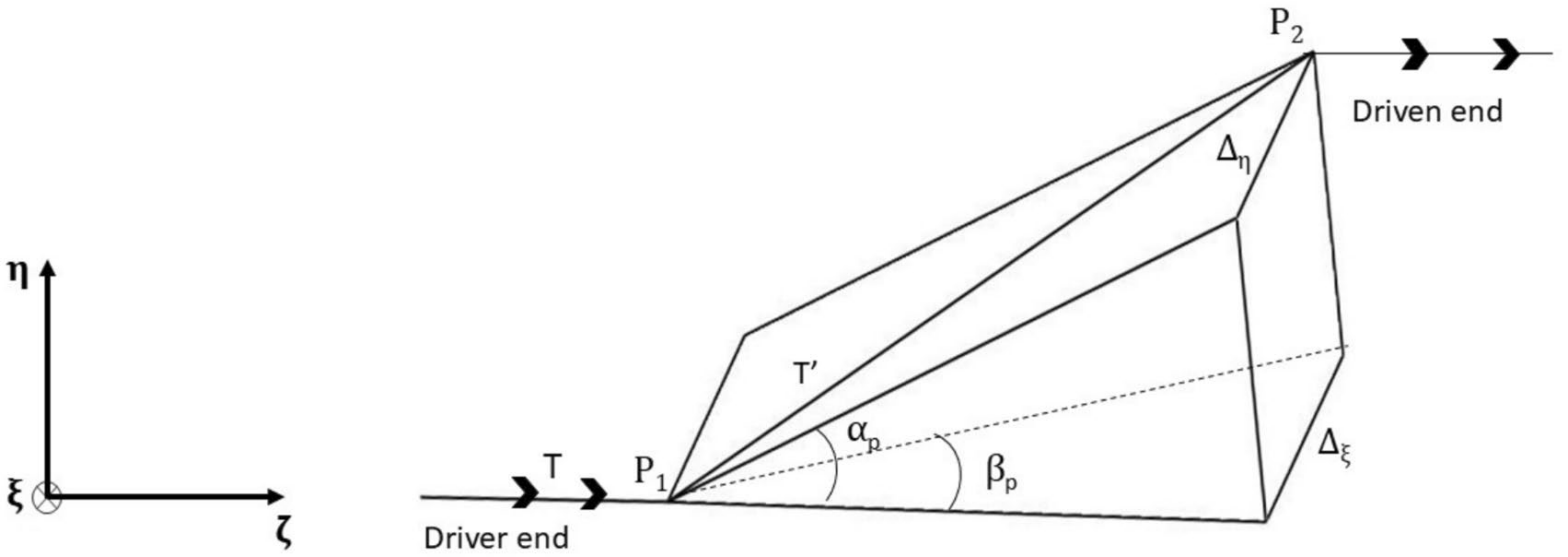
Torque transmitted across coupling ends P_1_ and P_2_ in the case of parallel misalignment.

Fig. 10 shows the torque transmitted under parallel misalignment. Let ∆_η_ and ∆_ξ_ be the known values of parallel misalignment present in the η and ξ directions, respectively. Let T and T’ be the torque applied by the driver end and the torque transmitted across the coupling P1-P2, respectively.

Resolving $\mathrm{T}^{\prime }$ at end ${\mathrm{C}}_{1}$ we get,

35 $${\mathrm{M}}_{\upxi } = {\mathrm{T}}^{\prime } \sin \left( {\upbeta_{{\mathrm{p}}} } \right)$$

36 $${\mathrm{M}}_{\upeta } = {\mathrm{T}}^{\prime } \cos \left( {\upbeta_{{\mathrm{p}}} } \right)\sin \left( {\upalpha_{{\mathrm{p}}} } \right)$$

37 $${\mathrm{M}}_{\upzeta } = \mathrm{T}^{\prime}\cos \left( {\upbeta_{{\mathrm{p}}} } \right)\cos \left( {\upalpha_{{\mathrm{p}}} } \right)$$

$${\Delta }_{\upxi }= {\mathrm{l}}_{\mathrm{h}}\mathrm{sin}\left({\upbeta }_{\mathrm{p}}\right)\text{ and }\Delta \upeta ={\mathrm{l}}_{\mathrm{h}}\mathrm{cos}\left({\upbeta }_{\mathrm{p}}\right)\mathrm{sin}\left({\upalpha }_{\mathrm{p}}\right)$$

For small values of $\upbeta$_p_ and $\upalpha$_p_

38 $${\mathrm{M}}_{\upzeta } \approx {\mathrm{T}}^{\prime } \approx {\mathrm{T}}$$

39 $${\mathrm{M}}_{\upxi } = {\mathrm{T}}^{\prime } \sin \left( {\upbeta_{p} } \right) \approx \frac{{\mathrm{T}}}{{{\mathrm{l}}_{{\mathrm{h}}} }}\Delta_{\upxi }$$

40 $${\mathrm{M}}_{\upeta } = {\mathrm{T}}^{\prime } \cos \left( {\upbeta_{{\mathrm{p}}} } \right)\sin \left( {\upalpha_{{\mathrm{p}}} } \right) \approx \frac{{\mathrm{T}}}{{{\mathrm{l}}_{{\mathrm{h}}} }}\Delta_{\upeta }$$

Similarly resolving ${\mathrm{T}}^{\prime }$ at end ${\mathrm{P}}_{2}$ we get the same values for ${\mathrm{M}}_{\upzeta }$, ${\mathrm{M}}_{\upxi }$ and ${\mathrm{M}}_{\upeta }$ for the driven end.

#### Torque with angular misalignment

**Fig. 11:**
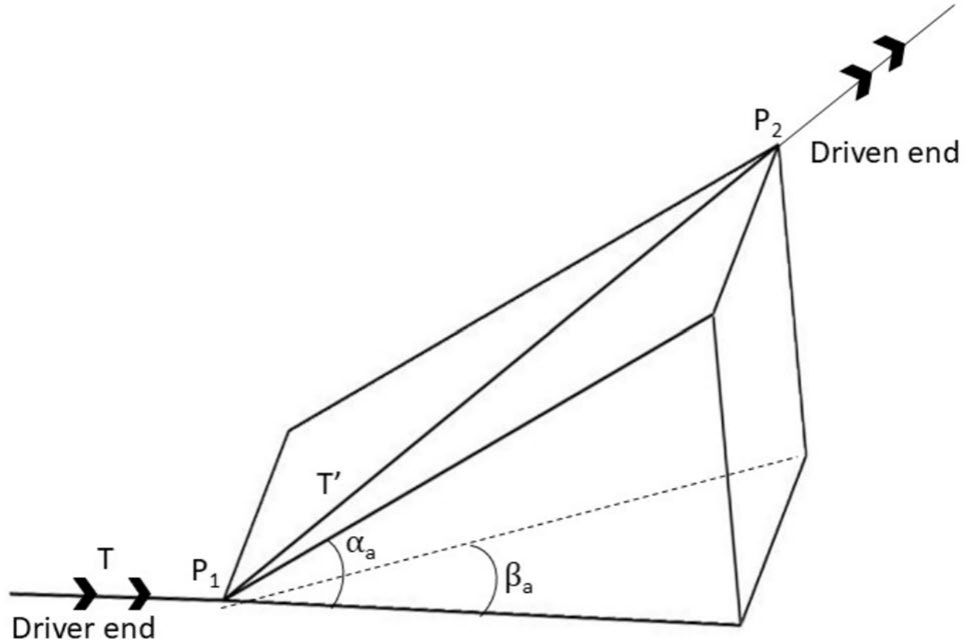
Torque transmitted across coupling ends P_1_ and P_2_ in angular misalignment.

Fig. 11 shows the torque transmitted in the presence of angular misalignment. Let ‘$\upalpha_{{\mathrm{a}}}$’ and ‘$\upbeta_{{\mathrm{a}}}$’ be the known values of angular misalignments about $\upxi$ and $\upeta$ axis respectively. The final shaft axis after misalignment now lies along P_1_-P_2_.

For small values of $\alpha_{{\mathrm{a}}}$ and ${\upbeta }_{\mathrm{a}}$, as typically the values lie within 1 to 3 degrees, resolving T along the axis P_1_-P_2_ and its perpendicular axes, the following expressions are derived.

41 $${\mathrm{M}}_{\upzeta } = {\mathrm{T}}^{\prime } = {\mathrm{T}}\cos \left( {\upalpha_{{\mathrm{a}}} } \right)\cos \left( {\upbeta_{{\mathrm{a}}} } \right) \approx {\mathrm{T}}$$

42 $${\mathrm{M}}_{\upxi } = - {\mathrm{T}}\cos \left( {\upalpha_{{\mathrm{a}}} } \right)\sin \left( {\upbeta_{{\mathrm{a}}} } \right) \approx - {\mathrm{T}}\upbeta_{{\mathrm{a}}}$$

43 $${\mathrm{M}}_{\upeta } = - {\mathrm{T}}\sin \left( {\upalpha_{{\mathrm{a}}} } \right) \approx - {\mathrm{T}}\upalpha_{{\mathrm{a}}}$$

## Equations of motion

**Fig. 12:**
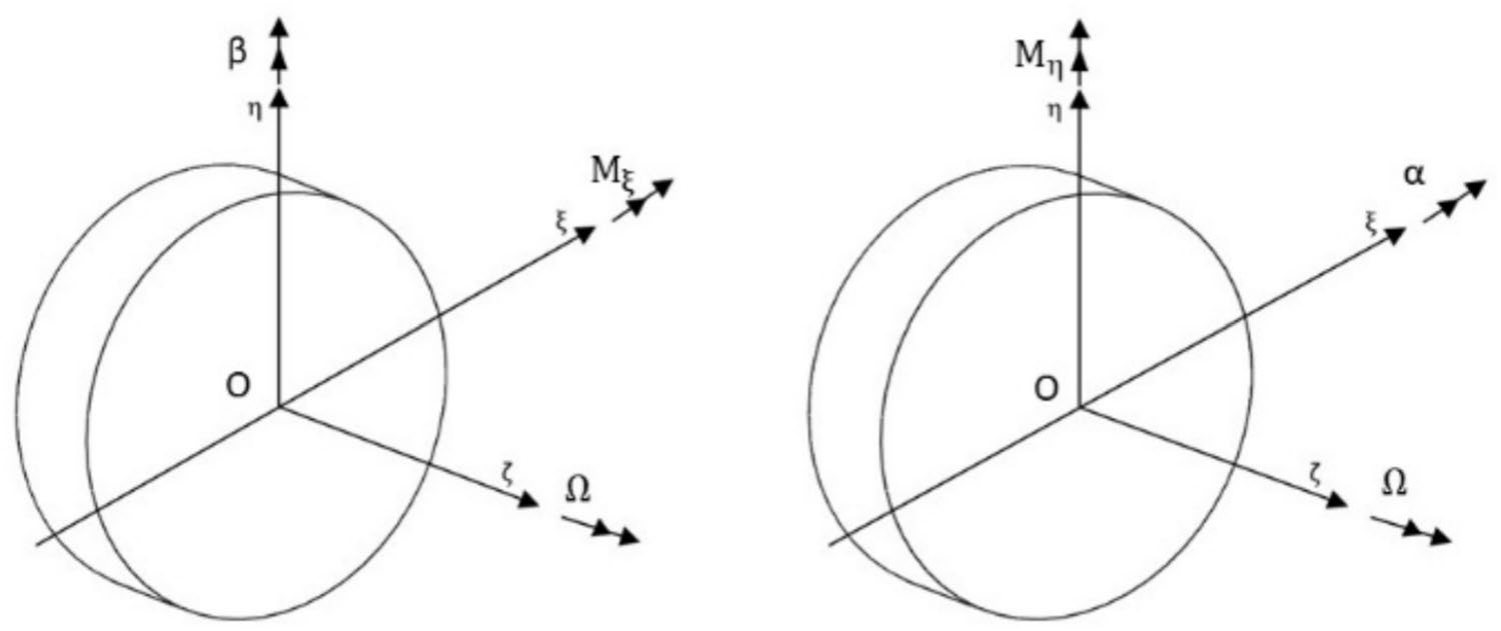
Vector diagram showing moments and angles in different directions at disc location.

Equations of motion of the rotor system with 4 degrees of freedom in the rotating coordinate frame are written down as in equations 44-47, after following^24–28^. In doing so, the forces and moments due to unbalance, gravity, torque transmitted and misalignment are taken and are denoted by superscripts $^{\prime } {\mathrm{un}}^{\prime }$, $^{\prime } {\mathrm{g}}^{\prime }$, $^{\prime } {\mathrm{T}}^{\prime }$ and $^{\prime } {\mathrm{ma}}^{\prime }$ respectively, whereas the subscripts represent the direction along and about which these forces and moments act, respectively. Fig. 12 represent the moments and angle in different directions at the disc location guiding in forming the characteristics equation of motion for the system.

44 $$\begin{gathered} {\mathrm{m}}\ddot{\upxi } - 2{\mathrm{m}}\Omega \dot{\upeta } + {\mathrm{c}}_{{{\mathrm{b}}_{\upxi } }} \dot{\upxi } + {\mathrm{c}}_{{{\mathrm{b}}_{{\upxi {\mathrm{c}}}} }} \dot{\upbeta } + {\mathrm{k}}_{{{\mathrm{b}}_{\upxi } }} \upxi + k_{{{\mathrm{b}}_{{\upxi {\mathrm{c}}}} }} \upbeta - {\mathrm{m}}\Omega ^{2} \upxi + {\mathrm{k}}_{{\upxi {\mathrm{t}}}} \left( {} \right)\upxi \hfill \\ + {\mathrm{k}}_{{\upxi {\mathrm{c}}}} \left( {} \right)\upbeta = {\mathrm{F}}_{\upxi }^{{{\mathrm{ma}}}} + {\mathrm{F}}_{\upxi }^{{{\mathrm{un}}}} + {\mathrm{F}}_{\upxi }^{{\mathrm{g}}} \hfill \\ \end{gathered}$$

45 $$\begin{gathered} {\mathrm{m}}\ddot{\upeta } - 2{\mathrm{m}}\Omega \dot{\upxi } + {\mathrm{c}}_{{{\mathrm{b}}_{\upeta } }} \dot{\upeta } - {\mathrm{c}}_{{{\mathrm{b}}_{{\upeta {\mathrm{c}}}} }} \dot{\upalpha } + {\mathrm{k}}_{{{\mathrm{b}}_{\upeta } }} \upeta - {\mathrm{k}}_{{{\mathrm{b}}_{{\upeta {\mathrm{c}}}} }} \upalpha - {\mathrm{m}}\Omega ^{2} \upeta + {\mathrm{k}}_{{\upeta {\mathrm{t}}}} \left( {} \right)\upeta \hfill \\ - {\mathrm{k}}_{{\upeta {\mathrm{c}}}} \left( {} \right)\upalpha = {\mathrm{F}}_{\upeta }^{{{\mathrm{ma}}}} + {\mathrm{F}}_{\upeta }^{{{\mathrm{un}}}} + {\mathrm{F}}_{\upeta }^{{\mathrm{g}}} \hfill \\ \end{gathered}$$

46 $$\begin{gathered} {\mathrm{I}}_{{\mathrm{d}}} \ddot{\upalpha } + \Omega \left( { - 2{\mathrm{I}}_{{\mathrm{d}}} + {\mathrm{I}}_{{\mathrm{p}}} } \right)\dot{\upbeta } - \Omega ^{2} \left( {\mathrm{I}_{{\mathrm{d}}} - \mathrm{I}_{{\mathrm{p}}} } \right)\upalpha - {\mathrm{c}}_{{{\mathrm{b}}_{{\upeta {\mathrm{c}}}} }} \dot{\upeta } + {\mathrm{c}}_{{{\mathrm{b}}_{\upalpha } }} \dot{\upalpha } - {\mathrm{k}}_{{{\mathrm{b}}_{{\upeta {\mathrm{c}}}} }} \upeta + {\mathrm{k}}_{{{\mathrm{b}}_{\upalpha } }} \upalpha \hfill \\ - {\mathrm{k}}_{{\upeta {\mathrm{c}}}} \left( {} \right)\upeta + {\mathrm{k}}_{{\upeta {\mathrm{r}}}} \left( {} \right)\upalpha = {\mathrm{M}}_{\upxi }^{{{\mathrm{ma}}}} + {\mathrm{M}}_{\upxi }^{{\mathrm{T}}} \hfill \\ \end{gathered}$$

47 $$\begin{gathered} {\mathrm{I}}_{{\mathrm{d}}} \ddot{\upbeta } + \Omega \left( {2{\mathrm{I}}_{{\mathrm{d}}} - {\mathrm{I}}_{p} } \right)\dot{\upalpha } - \Omega ^{2} \left( {{\mathrm{I}}_{{\mathrm{d}}} - {\mathrm{I}}_{{\mathrm{p}}} } \right)\upbeta + {\mathrm{c}}_{{{\mathrm{b}}_{{\upxi {\mathrm{c}}}} }} \dot{\upxi } + {\mathrm{c}}_{{{\mathrm{b}}_{\upbeta } }} \dot{\upbeta } + {\mathrm{k}}_{{{\mathrm{b}}_{{\upxi {\mathrm{c}}}} }} \upxi + {\mathrm{k}}_{{{\mathrm{b}}_{\upbeta } }} \upbeta \hfill \\ + {\mathrm{k}}_{{\upxi {\mathrm{c}}}} \left( {} \right)\upxi + {\mathrm{k}}_{{\upxi {\mathrm{r}}}} \left( {} \right)\upbeta = {\mathrm{M}}_{\upeta }^{{{\mathrm{ma}}}} + \mathrm{M}_{\upeta }^{{\mathrm{T}}} \hfill \\ \end{gathered}$$

In the above equations ${\mathrm{I}}_{\mathrm{d}}$ and ${\mathrm{I}}_{\mathrm{p}}$ are the diametral and polar mass moments of inertia of the disc, respectively, m is the mass of the disc, $\Omega$ is the angular velocity of the shaft.

The stiffness operators can be derived by the procedure mentioned in given in the supplementary material (Appendix 1 (a)), from which, ${\mathrm{k}}_{{\upeta {\mathrm{t}}}} \left( {} \right) = {\mathrm{K}}_{{\mathrm{s}}} {\mathrm{A}}_{{\upeta {\mathrm{t}}}} \left( {} \right),$ ${\mathrm{k}}_{{\upeta {\mathrm{c}}}} \left( {} \right) = {\mathrm{K}}_{{\mathrm{s}}} {\mathrm{lA}}_{{\upeta {\mathrm{c}}}} \left( {} \right),$ ${\mathrm{k}}_{{\upeta {\mathrm{r}}}} \left( {} \right) = {\mathrm{K}}_{{\mathrm{s}}} {\mathrm{l}}^{2} {\mathrm{A}}_{{\upeta {\mathrm{r}}}} \left( {} \right),$ ${\mathrm{k}}_{{\upxi {\mathrm{t}}}} \left( {} \right) = {\mathrm{K}}_{{\mathrm{s}}} {\mathrm{A}}_{{\upxi {\mathrm{t}}}} \left( {} \right),$ ${\mathrm{k}}_{{\upxi {\mathrm{c}}}} \left( {} \right) = {\mathrm{K}}_{{\mathrm{s}}} {\mathrm{lA}}_{{\upxi {\mathrm{c}}}} \left( {} \right),$ ${\mathrm{k}}_{{\upxi {\mathrm{r}}}} \left( {} \right) = {\mathrm{K}}_{{\mathrm{s}}} {\mathrm{l}}^{2} {\mathrm{A}}_{{\upxi {\mathrm{r}}}} \left( {} \right)$.

In the above derivations, the expressions of ${\mathrm{A}}_{\mathrm{ij}}\left(\right)$ has been given in the supplementary material (Appendix 1 (b)). An indicial representation of ${\mathrm{A}}_{\mathrm{ij}}\left(\right)$ may be written as $\frac{{{\mathrm{q}}_{{0}}^{{{\mathrm{ij}}}} + {\mathrm{q}}_{{1}}^{{{\mathrm{ij}}}} \mathrm{D}^{\prime } + {\mathrm{q}}_{{2}}^{{{\mathrm{ij}}}} \mathrm{D}^{\prime 2} }}{{{\mathrm{r}}_{{0}}^{{{\mathrm{ij}}}} + {\mathrm{r}}_{{1}}^{{{\mathrm{ij}}}} \mathrm{D}^{\prime } + {\mathrm{r}}_{{2}}^{{{\mathrm{ij}}}} \mathrm{D}^{\prime 2} }}$. Expressions of ${\mathrm{A}}_{\mathrm{ij}}\left(\right)$ for the six-link coupling, assumed for finding results, are given in the ‘Results and Discussion’ section.

The above equations of motion (Eqs. 44–47) can also be written in the form of a matrix as follows.

48 $$\begin{gathered} \left[ {\begin{array}{*{20}c} {\begin{array}{*{20}c} {\begin{array}{*{20}c} {\mathrm{m}} \\ 0 \\ \end{array} } \\ 0 \\ 0 \\ \end{array} } & {\begin{array}{*{20}c} {\begin{array}{*{20}c} 0 \\ {\mathrm{m}} \\ \end{array} } \\ 0 \\ 0 \\ \end{array} } & {\begin{array}{*{20}c} {\begin{array}{*{20}c} {\begin{array}{*{20}c} 0 \\ 0 \\ \end{array} } \\ {{\mathrm{I}}_{{\mathrm{d}}} } \\ 0 \\ \end{array} } & {\begin{array}{*{20}c} {\begin{array}{*{20}c} 0 \\ 0 \\ \end{array} } \\ 0 \\ {{\mathrm{I}}_{{\mathrm{d}}} } \\ \end{array} } \\ \end{array} } \\ \end{array} } \right]\left\{ {\begin{array}{*{20}c} {\begin{array}{*{20}c} {\ddot{\upxi }} \\ {\ddot{\upeta }} \\ \end{array} } \\ {\ddot{\upalpha }} \\ {\ddot{\upbeta }} \\ \end{array} } \right\} \hfill \\ + \left( {\left[ {\begin{array}{*{20}c} {\begin{array}{*{20}c} {\begin{array}{*{20}c} 0 \\ { - 2{\text{m }}\Omega } \\ \end{array} } \\ 0 \\ 0 \\ \end{array} } & {\begin{array}{*{20}c} {\begin{array}{*{20}c} { - 2{\text{m }}\Omega } \\ 0 \\ \end{array} } \\ 0 \\ 0 \\ \end{array} } & {\begin{array}{*{20}c} {\begin{array}{*{20}c} {\begin{array}{*{20}c} 0 \\ 0 \\ \end{array} } \\ 0 \\ {\Omega (2{\mathrm{I}}_{{\mathrm{d}}} - {\mathrm{I}}_{{\mathrm{p}}} )} \\ \end{array} } & {\begin{array}{*{20}c} {\begin{array}{*{20}c} 0 \\ 0 \\ \end{array} } \\ {\Omega ( - 2{\mathrm{I}}_{{\mathrm{d}}} + {\mathrm{I}}_{{\mathrm{p}}} )} \\ 0 \\ \end{array} } \\ \end{array} } \\ \end{array} } \right]} \right. \hfill \\ + \left. {\left[ {\begin{array}{*{20}c} {{\mathrm{c}}_{{{\mathrm{b}}_{\upxi } }} } & 0 & 0 & {{\mathrm{c}}_{{{\mathrm{b}}_{{\upxi {\mathrm{c}}}} }} } \\ 0 & {{\mathrm{c}}_{{{\mathrm{b}}_{\upeta } }} } & { - {\mathrm{c}}_{{{\mathrm{b}}_{{\upeta {\mathrm{c}}}} }} } & 0 \\ 0 & { - {\mathrm{c}}_{{{\mathrm{b}}_{{\upeta {\mathrm{c}}}} }} } & {{\mathrm{c}}_{{{\mathrm{b}}_{\upalpha } }} } & 0 \\ {{\mathrm{c}}_{{{\mathrm{b}}_{{\upxi {\mathrm{c}}}} }} } & 0 & 0 & {{\mathrm{c}}_{{{\mathrm{b}}_{\upbeta } }} } \\ \end{array} } \right]} \right)\left\{ {\begin{array}{*{20}c} {\begin{array}{*{20}c} {\dot{\upxi }} \\ {\dot{\upeta }} \\ \end{array} } \\ {\dot{\upalpha }} \\ {\dot{\upbeta }} \\ \end{array} } \right\} \hfill \\ + \left( {\left[ {\begin{array}{*{20}c} {\begin{array}{*{20}c} {\begin{array}{*{20}c} { - {\text{ m}}\Omega ^{2} } \\ 0 \\ \end{array} } \\ 0 \\ 0 \\ \end{array} } & {\begin{array}{*{20}c} {\begin{array}{*{20}c} 0 \\ { - {\text{ m}}\Omega ^{2} } \\ \end{array} } \\ 0 \\ 0 \\ \end{array} } & {\begin{array}{*{20}c} {\begin{array}{*{20}c} {\begin{array}{*{20}c} 0 \\ 0 \\ \end{array} } \\ { - \Omega ^{2} ({\mathrm{I}}_{{\mathrm{d}}} - {\mathrm{I}}_{{\mathrm{p}}} )} \\ 0 \\ \end{array} } & {\begin{array}{*{20}c} {\begin{array}{*{20}c} 0 \\ 0 \\ \end{array} } \\ 0 \\ { - \Omega ^{2} ({\mathrm{I}}_{{\mathrm{d}}} - {\mathrm{I}}_{{\mathrm{p}}} )} \\ \end{array} } \\ \end{array} } \\ \end{array} } \right]} \right. \hfill \\ + \left[ {\begin{array}{*{20}c} {\begin{array}{*{20}c} {\begin{array}{*{20}c} {{\mathrm{k}}_{{{\mathrm{b}}_{\upxi } }} } \\ 0 \\ \end{array} } \\ 0 \\ {{\mathrm{k}}_{{{\mathrm{b}}_{{\upxi {\mathrm{c}}}} }} } \\ \end{array} } & {\begin{array}{*{20}c} {\begin{array}{*{20}c} 0 \\ {{\mathrm{k}}_{{{\mathrm{b}}_{\upeta } }} } \\ \end{array} } \\ { - {\mathrm{k}}_{{{\mathrm{b}}_{{\upeta {\mathrm{c}}}} }} } \\ 0 \\ \end{array} } & {\begin{array}{*{20}c} {\begin{array}{*{20}c} {\begin{array}{*{20}c} 0 \\ { - {\mathrm{k}}_{{{\mathrm{b}}_{{\upeta {\mathrm{c}}}} }} } \\ \end{array} } \\ {{\mathrm{k}}_{{{\mathrm{b}}_{\upalpha } }} } \\ 0 \\ \end{array} } & {\begin{array}{*{20}c} {\begin{array}{*{20}c} {{\mathrm{k}}_{{{\mathrm{b}}_{{\upxi {\mathrm{c}}}} }} } \\ 0 \\ \end{array} } \\ 0 \\ {{\mathrm{k}}_{{{\mathrm{b}}_{\upbeta } }} } \\ \end{array} } \\ \end{array} } \\ \end{array} } \right] \hfill \\ \left. { + \left[ {\begin{array}{*{20}c} {{\mathrm{K}}_{{\mathrm{s}}} {\mathrm{A}}_{{\upxi {\mathrm{t}}}} ()} & 0 & 0 & {{\mathrm{K}}_{{\mathrm{s}}} {\mathrm{lA}}_{{\upxi {\mathrm{c}}}} ()} \\ 0 & {{\mathrm{K}}_{{\mathrm{s}}} {\mathrm{A}}_{{\upeta {\mathrm{t}}}} ()} & { - {\mathrm{K}}_{{\mathrm{s}}} {\mathrm{lA}}_{{\upeta {\mathrm{c}}}} ()} & 0 \\ 0 & { - {\mathrm{K}}_{{\mathrm{s}}} {\mathrm{lA}}_{{\upeta {\mathrm{c}}}} ()} & {{\mathrm{K}}_{{\mathrm{s}}} {\mathrm{l}}^{2} {\mathrm{A}}_{{\upeta {\mathrm{r}}}} ()} & 0 \\ {{\mathrm{K}}_{{\mathrm{s}}} {\mathrm{lA}}_{{\upxi {\mathrm{c}}}} ()} & 0 & 0 & {{\mathrm{K}}_{{\mathrm{s}}} {\mathrm{l}}^{2} {\mathrm{A}}_{{\upxi {\mathrm{r}}}} ()} \\ \end{array} } \right]} \right)\left\{ {\begin{array}{*{20}c} \upxi \\ \upeta \\ \upalpha \\ \upbeta \\ \end{array} } \right\} \hfill \\ = \left\{ {\begin{array}{*{20}c} {({\mathrm{F}}_{\upxi }^{{{\mathrm{ma}}}} + {\mathrm{F}}_{\upxi }^{{{\mathrm{un}}}} + {\mathrm{F}}_{\upxi }^{{\mathrm{g}}} )} \\ {({\mathrm{F}}_{\upeta }^{{{\mathrm{ma}}}} + {\mathrm{F}}_{\upeta }^{{{\mathrm{un}}}} + {\mathrm{F}}_{\upeta }^{{\mathrm{g}}} )} \\ {{\mathrm{(M}}_{\upxi }^{{{\mathrm{ma}}}} + {\mathrm{M}}_{\upxi }^{{\mathrm{T}}} )} \\ {{\mathrm{(M}}_{\upeta }^{{{\mathrm{ma}}}} + {\mathrm{M}}_{\upeta }^{{\mathrm{T}}} )} \\ \end{array} } \right\} \hfill \\ \end{gathered}$$

### Stability and response analysis

#### Stability

For stability analysis, the homogeneous equations of motion (Eq. 48) are used. The values of ${\mathrm{K}}_{\mathrm{ij}}\left(\right)={\mathrm{K}}_{\mathrm{s}}{\mathrm{A}}_{\mathrm{ij}}\left(\right)$, where ‘i’ represents both $\upxi$ and $\upeta$ coordinates and ‘j’ represents both the transverse and coupling effect of the stiffness forces.

$$\begin{gathered} \mathrm{A}_{{\upeta \mathrm{t}}} \left( {} \right) = \left( {\frac{{\mathrm{q}_{0}^{{\upeta \mathrm{t}}} + \mathrm{q}_{1}^{{\upeta \mathrm{t}}} \mathrm{D}^{\prime } + \mathrm{q}_{2}^{{\upeta \mathrm{t}}} \mathrm{D}^{{\prime 2}} }}{{r_{0}^{{\upeta \mathrm{t}}} + r_{1}^{{\upeta \mathrm{t}}} \mathrm{D}^{\prime } + r_{2}^{{\upeta \mathrm{t}}} \mathrm{D}^{{\prime 2}} }}} \right),\,{\kern 1pt} {\kern 1pt} \mathrm{A}_{{\upeta \mathrm{c}}} \left( {} \right) = \left( {\frac{{\mathrm{q}_{0}^{{\upeta \mathrm{c}}} + \mathrm{q}_{1}^{{\upeta \mathrm{c}}} \mathrm{D}^{\prime } + \mathrm{q}_{2}^{{\upeta c}} \mathrm{D}^{{\prime 2}} }}{{\mathrm{r}_{0}^{{\upeta \mathrm{c}}} + \mathrm{r}_{1}^{{\upeta  \mathrm{c}}} \mathrm{D}^{\prime } + \mathrm{r}_{2}^{{\upeta \mathrm{c}}} \mathrm{D}^{{\prime 2}} }}} \right),\,\mathrm{A}_{{\upeta \mathrm{r}}} \left( {} \right) \hfill \\ =\left( {\frac{{\mathrm{q}_{0}^{{\upeta \mathrm{r}}} + \mathrm{q}_{1}^{{\upeta r}} \mathrm{D}^{\prime } + \mathrm{q}_{2}^{{\upeta r}} \mathrm{D}^{{\prime 2}} }}{{\mathrm{r}_{0}^{{\upeta r}} + \mathrm{r}_{1}^{{\upeta \mathrm{r}}} \mathrm{D}^{\prime } + \mathrm{r}_{2}^{{\upeta \mathrm{r}}} \mathrm{D}^{{\prime 2}} }}} \right),\,\mathrm{A}_{{\upxi \mathrm{t}}} \left( {} \right) = \left( {\frac{{\mathrm{q}_{0}^{{\upxi \mathrm{t}}} + \mathrm{q}_{1}^{{\upxi \mathrm{t}}} \mathrm{D}^{\prime } + \mathrm{q}_{2}^{{\upxi \mathrm{t}}} \mathrm{D}^{{\prime 2}} }}{{\mathrm{r}_{0}^{{\upxi \mathrm{t}}} + \mathrm{r}_{1}^{{\upxi \mathrm{t}}} \mathrm{D}^{\prime } + \mathrm{r}_{2}^{{\upxi \mathrm{t}}} \mathrm{D}^{{\prime 2}} }}} \right),\,\mathrm{A}_{{\upxi c}} \left( {} \right) \hfill \\ = \left( {\frac{{\mathrm{q}_{0}^{{\upxi c}} + \mathrm{q}_{1}^{{\upxi c}} \mathrm{D}^{\prime } + \mathrm{q}_{2}^{{\upxi c}} \mathrm{D}^{{\prime 2}} }}{{\mathrm{r}_{0}^{{\upxi c}} + \mathrm{r}_{1}^{{\upxi c}} \mathrm{D}^{\prime } + \mathrm{r}_{2}^{{\upxi c}} \mathrm{D}^{{\prime 2}} }}} \right),\,\mathrm{A}_{{\upxi \mathrm{r}}} \left( {} \right) = \left( {\frac{{\mathrm{q}_{0}^{{\upxi \mathrm{r}}} + \mathrm{q}_{1}^{{\upxi \mathrm{r}}} \mathrm{D}^{\prime } + \mathrm{q}_{2}^{{\upxi r}} \mathrm{D}^{{\prime 2}} }}{{\mathrm{r}_{0}^{{\upxi \mathrm{r}}} + \mathrm{r}_{1}^{{\upxi \mathrm{r}}} \mathrm{D}^{\prime } + \mathrm{r}_{2}^{{\upxi r}} \mathrm{D}^{{\prime 2}} }}} \right) \hfill \\ \end{gathered}$$

Putting the values of ${\mathrm{A}}_{\mathrm{ij}}\left(\right)$ in the equations (48), the modified equation can be seen as,

49 $$\begin{gathered} \left[ \begin{array}{*{20}c} \begin{array}{*{20}c} \begin{array}{*{20}c} {\mathrm{m}} \\ 0 \\ \end{array}  \\ 0 \\ 0 \\ \end{array}  & \begin{array}{*{20}c} {\begin{array}{*{20}c} 0 \\ {\mathrm{m}} \\ \end{array} } \\ 0 \\ 0 \\ \end{array}  & \begin{array}{*{20}c} \begin{array}{*{20}c} {\begin{array}{*{20}c} 0 \\ 0 \\ \end{array} } \\ {{\mathrm{I}}_{{\mathrm{d}}} } \\ 0 \\ \end{array}  & \begin{array}{*{20}c} {\begin{array}{*{20}c} 0 \\ 0 \\ \end{array} } \\ 0 \\ {{\mathrm{I}}_{{\mathrm{d}}} } \\ \end{array}  \\ \end{array}  \\ \end{array}  \right]\left\{ {\begin{array}{*{20}c} {\begin{array}{*{20}c} {\ddot{\upxi }} \\ {\ddot{\upeta }} \\ \end{array} } \\ {\ddot{\upalpha }} \\ {\ddot{\upbeta }} \\ \end{array} } \right\} \hfill \\ + \left( \left[ \begin{array}{*{20}c} {\begin{array}{*{20}c} {\begin{array}{*{20}c} 0 \\ { - 2{\mathrm{m}}\Omega } \\ \end{array} } \\ 0 \\ 0 \\ \end{array} } & {\begin{array}{*{20}c} {\begin{array}{*{20}c} { - 2{\mathrm{m}}\Omega } \\ 0 \\ \end{array} } \\ 0 \\ 0 \\ \end{array} } & \begin{array}{*{20}c} {\begin{array}{*{20}c} {\begin{array}{*{20}c} 0 \\ 0 \\ \end{array} } \\ 0 \\ {\Omega (2{\mathrm{I}}_{{\mathrm{d}}} - {\mathrm{I}}_{{\mathrm{p}}} )} \\ \end{array} } & {\begin{array}{*{20}c} {\begin{array}{*{20}c} 0 \\ 0 \\ \end{array} } \\ {\Omega ( - 2{\mathrm{I}}_{{\mathrm{d}}} + {\mathrm{I}}_{{\mathrm{p}}} )} \\ 0 \\ \end{array} } \\ \end{array}  \\ \end{array}  \right] \right. \hfill \\ + \left. {\left[ {\begin{array}{*{20}c} \begin{array}{*{20}c} \begin{array}{*{20}c} {{\mathrm{c}}_{{{\mathrm{b}}_{\upxi } }} } \\ 0 \\ \end{array}  \\ 0 \\ {{\mathrm{c}}_{{{\mathrm{b}}_{{\upxi {\mathrm{c}}}} }} } \\ \end{array}  & {\begin{array}{*{20}c} {\begin{array}{*{20}c} 0 \\ {{\mathrm{c}}_{{{\mathrm{b}}_{\upeta } }} } \\ \end{array} } \\ { - {\mathrm{c}}_{{{\mathrm{b}}_{{\upeta {\mathrm{c}}}} }} } \\ 0 \\ \end{array} } & {\begin{array}{*{20}c} {\begin{array}{*{20}c} {\begin{array}{*{20}c} 0 \\ { - {\mathrm{c}}_{{{\mathrm{b}}_{{\upeta {\mathrm{c}}}} }} } \\ \end{array} } \\ {{\mathrm{c}}_{{{\mathrm{b}}_{\upalpha } }} } \\ 0 \\ \end{array} } & {\begin{array}{*{20}c} {\begin{array}{*{20}c} {{\mathrm{c}}_{{{\mathrm{b}}_{{\upxi {\mathrm{c}}}} }} } \\ 0 \\ \end{array} } \\ 0 \\ {{\mathrm{c}}_{{{\mathrm{b}}_{\upbeta } }} } \\ \end{array} } \\ \end{array} } \\ \end{array} } \right]} \right)\left\{ {\begin{array}{*{20}c} {\begin{array}{*{20}c} {\dot{\upxi }} \\ {\dot{\upeta }} \\ \end{array} } \\ {\dot{\upalpha }} \\ {\dot{\upbeta }} \\ \end{array} } \right\} \hfill \\ + \left( {\left[ {\begin{array}{*{20}c} {\begin{array}{*{20}c} { - {\text{ m}}\Omega ^{2} } \\ 0 \\ 0 \\ \end{array} } & {\begin{array}{*{20}c} {\begin{array}{*{20}c} 0 \\ { - {\text{ m}}\Omega ^{2} } \\ \end{array} } \\ 0 \\ 0 \\ \end{array} } & {\begin{array}{*{20}c} {\begin{array}{*{20}c} {\begin{array}{*{20}c} 0 \\ 0 \\ \end{array} } \\ { - \Omega ^{2} ({\mathrm{I}}_{{\mathrm{d}}} - {\mathrm{I}}_{{\mathrm{p}}} )} \\ 0 \\ \end{array} } & {\begin{array}{*{20}c} {\begin{array}{*{20}c} 0 \\ 0 \\ \end{array} } \\ 0 \\ { - \Omega ^{2} ({\mathrm{I}}_{{\mathrm{d}}} - {\mathrm{I}}_{{\mathrm{p}}} )} \\ \end{array} } \\ \end{array} } \\ \end{array} } \right]} \right. + \left[ {\begin{array}{*{20}c} {{\mathrm{k}}_{{{\mathrm{b}}_{\upxi } }} } & 0 & 0 & {{\mathrm{k}}_{{{\mathrm{b}}_{{\upxi {\mathrm{c}}}} }} } \\ 0 & {{\mathrm{k}}_{{{\mathrm{b}}_{\upeta } }} } & { - {\mathrm{k}}_{{{\mathrm{b}}_{{\upeta {\mathrm{c}}}} }} } & 0 \\ 0 & { - {\mathrm{k}}_{{{\mathrm{b}}_{{\upeta {\mathrm{c}}}} }} } & {{\mathrm{k}}_{{{\mathrm{b}}_{\upalpha } }} } & 0 \\ {{\mathrm{k}}_{{{\mathrm{b}}_{{\upxi {\mathrm{c}}}} }} } & 0 & 0 & {{\mathrm{k}}_{{{\mathrm{b}}_{\upbeta } }} } \\ \end{array} } \right] \hfill \\ + {\mathrm{K}}_{{\mathrm{S}}} \tiny \left. \left[ \begin{array}{*{20}c} \left( {\frac{{\mathrm{q}_{0}^{{\upxi \mathrm{t}}} + \mathrm{q}_{1}^{{\upxi \mathrm{t}}} \mathrm{D}^{\prime } + \mathrm{q}_{2}^{{\upxi \mathrm{t}}} \mathrm{D}^{{\prime 2}} }}{{\mathrm{r}_{0}^{{\upxi \mathrm{t}}} + \mathrm{r}_{1}^{{\upxi \mathrm{t}}} \mathrm{D}^{\prime } + \mathrm{r}_{2}^{{\upxi \mathrm{t}}} \mathrm{D}^{{\prime 2}} }}} \right) & 0 & 0 & {\mathrm{l}}\left( {\frac{{\mathrm{q}_{0}^{{\upxi c}} + \mathrm{q}_{1}^{{\upxi c}} \mathrm{D}^{\prime } + \mathrm{q}_{2}^{{\upxi c}} \mathrm{D}^{{\prime 2}} }}{{\mathrm{r}_{0}^{{\upxi c}} + \mathrm{r}_{1}^{{\upxi c}} \mathrm{D}^{\prime } + \mathrm{r}_{2}^{{\upxi c}} \mathrm{D}^{{\prime 2}} }}} \right) \\ 0 & \left( {\frac{{\mathrm{q}_{0}^{{\upeta \mathrm{t}}} + \mathrm{q}_{1}^{{\upeta \mathrm{t}}} \mathrm{D}^{\prime } + \mathrm{q}_{2}^{{\upeta \mathrm{t}}} \mathrm{D}^{{\prime 2}} }}{{r_{0}^{{\upeta \mathrm{t}}} + r_{1}^{{\upeta \mathrm{t}}} \mathrm{D}^{\prime } + r_{2}^{{\upeta \mathrm{t}}} \mathrm{D}^{{\prime 2}} }}} \right) & {\left( {\frac{{\mathrm{q}_{0}^{{\upeta \mathrm{c}}} + \mathrm{q}_{1}^{{\upeta \mathrm{c}}} \mathrm{D}^{\prime } + \mathrm{q}_{2}^{{\upeta c}} \mathrm{D}^{{\prime 2}} }}{{\mathrm{r}_{0}^{{\upeta \mathrm{c}}} + \mathrm{r}_{1}^{{\upeta  \mathrm{c}}} \mathrm{D}^{\prime } + \mathrm{r}_{2}^{{\upeta \mathrm{c}}} \mathrm{D}^{{\prime 2}} }}} \right)} & 0 \\ 0 & { - {\mathrm{l}} \left( {\frac{{\mathrm{q}_{0}^{{\upeta \mathrm{c}}} + \mathrm{q}_{1}^{{\upeta \mathrm{c}}} \mathrm{D}^{\prime } + \mathrm{q}_{2}^{{\upeta c}} \mathrm{D}^{{\prime 2}} }}{{\mathrm{r}_{0}^{{\upeta \mathrm{c}}} + \mathrm{r}_{1}^{{\upeta  \mathrm{c}}} \mathrm{D}^{\prime } + \mathrm{r}_{2}^{{\upeta \mathrm{c}}} \mathrm{D}^{{\prime 2}} }}} \right)} & {\left( {\frac{{\mathrm{q}_{0}^{{\upeta \mathrm{r}}} + \mathrm{q}_{1}^{{\upeta r}} \mathrm{D}^{\prime } + \mathrm{q}_{2}^{{\upeta r}} \mathrm{D}^{{\prime 2}} }}{{\mathrm{r}_{0}^{{\upeta r}} + \mathrm{r}_{1}^{{\upeta \mathrm{r}}} \mathrm{D}^{\prime } + \mathrm{r}_{2}^{{\upeta \mathrm{r}}} \mathrm{D}^{{\prime 2}} }}} \right)} & 0 \\ {\left( {\frac{{\mathrm{q}_{0}^{{\upxi c}} + \mathrm{q}_{1}^{{\upxi c}} \mathrm{D}^{\prime } + \mathrm{q}_{2}^{{\upxi c}} \mathrm{D}^{{\prime 2}} }}{{\mathrm{r}_{0}^{{\upxi c}} + \mathrm{r}_{1}^{{\upxi c}} \mathrm{D}^{\prime } + \mathrm{r}_{2}^{{\upxi c}} \mathrm{D}^{{\prime 2}} }}} \right)} & 0 & 0 & {{\mathrm{l}}^{2} \left( {\frac{{\mathrm{q}_{0}^{{\upxi \mathrm{r}}} + \mathrm{q}_{1}^{{\upxi \mathrm{r}}} \mathrm{D}^{\prime } + \mathrm{q}_{2}^{{\upxi r}} \mathrm{D}^{{\prime 2}} }}{{\mathrm{r}_{0}^{{\upxi \mathrm{r}}} + \mathrm{r}_{1}^{{\upxi \mathrm{r}}} \mathrm{D}^{\prime } + \mathrm{r}_{2}^{{\upxi r}} \mathrm{D}^{{\prime 2}} }}} \right)} \\ \end{array}  \right] \right)\\\times\left\{ {\begin{array}{*{20}c} {\begin{array}{*{20}c} \upxi \\ \upeta \\ \end{array} } \\ \upalpha \\ \upbeta \\ \end{array} } \right\} = \left\{ {\begin{array}{*{20}c} {({\mathrm{F}}_{\upxi }^{{{\mathrm{ma}}}} + {\mathrm{F}}_{\upxi }^{{{\mathrm{un}}}} + {\mathrm{F}}_{\upxi }^{{\mathrm{g}}} )} \\ {({\mathrm{F}}_{\upeta }^{{{\mathrm{ma}}}} + {\mathrm{F}}_{\upeta }^{{{\mathrm{un}}}} + {\mathrm{F}}_{\upeta }^{{\mathrm{g}}} )} \\ {{\mathrm{(M}}_{\upxi }^{{{\mathrm{ma}}}} + {\mathrm{M}}_{\upxi }^{{\mathrm{T}}} )} \\ {{\mathrm{(M}}_{\upeta }^{{{\mathrm{ma}}}} + {\mathrm{M}}_{\upeta }^{{\mathrm{T}}} )} \\ \end{array} } \right\} \hfill\\ \end{gathered}$$

After operating on the above equations using the net denominator polynomial on the left-hand side for each row in the matrix, it can be considered as

$${\mathrm{D}}_{1} = ({\mathrm{r}}_{0}^{{\upxi {\mathrm{t}}}} + {\mathrm{r}}_{1}^{{\upxi {\mathrm{t}}}} {\mathrm{D}}^{\prime } {\text{ + r}}_{{2}}^{{\upxi {\mathrm{t}}}} {\text{ D}}^{\prime } )({\mathrm{r}}_{{0}}^{{\upxi {\mathrm{c}}}} {\text{ + r}}_{{1}}^{{\upxi {\mathrm{c}}}} {\mathrm{D}}^{\prime } {\text{ + r}}_{{2}}^{{\upxi {\mathrm{c}}}} {\mathrm{D}}^{\prime } );$$

$${\mathrm{D}}_{2} = \left( {{\mathrm{r}}_{0}^{{\upeta {\mathrm{t}}}} + {\mathrm{r}}_{1}^{{\upeta {\mathrm{t}}}} {\text{ D}}^{\prime } + {\mathrm{r}}_{2}^{{\upeta {\mathrm{t}}}} {\text{ D}}^{\prime 2} } \right)\left( {{\mathrm{r}}_{0}^{{\upeta {\mathrm{c}}}} + {\mathrm{r}}_{1}^{{\upeta {\mathrm{c}}}} {\text{ D}}^{\prime } + {\mathrm{r}}_{2}^{{\upeta {\mathrm{c}}}} {\text{ D}}^{\prime 2} } \right);$$

$${\mathrm{D}}_{3} = \left( {{\mathrm{r}}_{0}^{{\upeta {\mathrm{c}}}} + {\mathrm{r}}_{1}^{{\upeta {\mathrm{c}}}} {\text{ D}}^{\prime } + {\mathrm{r}}_{2}^{{\upeta {\mathrm{c}}}} {\text{ D}}^{\prime 2} } \right)\left( {{\mathrm{r}}_{0}^{{\upeta {\mathrm{r}}}} + {\mathrm{r}}_{1}^{{\upeta {\mathrm{r}}}} {\text{ D}}^{\prime } + {\mathrm{r}}_{2}^{{\upeta {\mathrm{r}}}} {\text{ D}}^{\prime 2} } \right);$$

$${\mathrm{D}}_{4} = \left( {{\text{ r}}_{0}^{{{\upxi \mathrm{r}}}} + {\mathrm{r}}_{1}^{{{\upxi \mathrm{r}}}} { } + {\mathrm{r}}_{2}^{{{\upxi \mathrm{r}}}} {\text{ D}}^{\prime 2} } \right)\left( {{\mathrm{r}}_{0}^{{{\upxi \mathrm{r}}}} + {\mathrm{r}}_{1}^{{{\upxi \mathrm{r}}}} {\text{ D}}^{\prime } + {\mathrm{r}}_{2}^{{{\upxi \mathrm{r}}}} {\text{ D}}^{\prime 2} } \right);$$

50 $$\begin{gathered} \left[ {\begin{array}{*{20}c} {\begin{array}{*{20}c} {\begin{array}{*{20}c} {{\mathrm{mD}}_{1} } \\ 0 \\ \end{array} } \\ 0 \\ 0 \\ \end{array} } & {\begin{array}{*{20}c} {\begin{array}{*{20}c} 0 \\ {{\mathrm{mD}}_{2} } \\ \end{array} } \\ 0 \\ 0 \\ \end{array} } & {\begin{array}{*{20}c} {\begin{array}{*{20}c} {\begin{array}{*{20}c} 0 \\ 0 \\ \end{array} } \\ {{\mathrm{I}}_{{\mathrm{d}}} {\mathrm{D}}_{3} } \\ 0 \\ \end{array} } & {\begin{array}{*{20}c} {\begin{array}{*{20}c} 0 \\ 0 \\ \end{array} } \\ 0 \\ {{\mathrm{I}}_{{\mathrm{d}}} {\mathrm{D}}_{4} } \\ \end{array} } \\ \end{array} } \\ \end{array} } \right]\left\{ {\begin{array}{*{20}c} {\begin{array}{*{20}c} {\ddot{\xi }} \\ {\ddot{\eta }} \\ \end{array} } \\ {\ddot{\alpha }} \\ {\ddot{\beta }} \\ \end{array} } \right\} \hfill \\ + \left( {\left[ {\begin{array}{*{20}c} 0 & { - 2{\mathrm{m}}\Omega {\mathrm{D}}_{{\mathrm{1}}} } & 0 & 0 \\ { - 2{\mathrm{m}}\Omega {\mathrm{D}}_{{\mathrm{2}}} } & 0 & 0 & 0 \\ 0 & 0 & 0 & {\Omega ( - 2{\mathrm{I}}_{{\mathrm{d}}} + {\mathrm{I}}_{{\mathrm{p}}} ){\mathrm{D}}_{3} } \\ 0 & 0 & {\Omega (2{\mathrm{I}}_{{\mathrm{d}}} - {\mathrm{I}}_{{\mathrm{p}}} ){\mathrm{D}}_{4} } & 0 \\ \end{array} } \right]} \right. + \left. {\left[ {\begin{array}{*{20}c} {\begin{array}{*{20}c} {\begin{array}{*{20}c} {{\mathrm{c}}_{{{\mathrm{b}}_{\xi } }} {\mathrm{D}}_{1} } \\ 0 \\ \end{array} } \\ 0 \\ {{\mathrm{c}}_{{{\mathrm{b}}_{{\xi {\mathrm{c}}}} }} {\mathrm{D}}_{4} } \\ \end{array} } & {\begin{array}{*{20}c} {\begin{array}{*{20}c} 0 \\ {{\mathrm{c}}_{{{\mathrm{b}}_{\eta } }} {\mathrm{D}}_{2} } \\ \end{array} } \\ { - {\mathrm{c}}_{{{\mathrm{b}}_{{\eta {\mathrm{c}}}} }} {\mathrm{D}}_{3} } \\ 0 \\ \end{array} } & {\begin{array}{*{20}c} {\begin{array}{*{20}c} {\begin{array}{*{20}c} 0 \\ { - {\mathrm{c}}_{{{\mathrm{b}}_{{\eta {\mathrm{c}}}} }} {\mathrm{D}}_{2} } \\ \end{array} } \\ {{\mathrm{c}}_{{{\mathrm{b}}_{\alpha } }} {\mathrm{D}}_{3} } \\ 0 \\ \end{array} } & {\begin{array}{*{20}c} {\begin{array}{*{20}c} {{\mathrm{c}}_{{{\mathrm{b}}_{{\xi {\mathrm{c}}}} }} {\mathrm{D}}_{1} } \\ 0 \\ \end{array} } \\ 0 \\ {{\mathrm{c}}_{{{\mathrm{b}}_{\beta } }} {\mathrm{D}}_{4} } \\ \end{array} } \\ \end{array} } \\ \end{array} } \right]} \right)\left\{ {\begin{array}{*{20}c} {\begin{array}{*{20}c} {\dot{\xi }} \\ {\dot{\eta }} \\ \end{array} } \\ {\dot{\alpha }} \\ {\dot{\beta }} \\ \end{array} } \right\} \hfill \\ + \left( {\left[ {\begin{array}{*{20}c} {\begin{array}{*{20}c} {\begin{array}{*{20}c} { - {\text{ m}}\Omega ^{2} {\mathrm{D}}_{1} } \\ 0 \\ \end{array} } \\ 0 \\ 0 \\ \end{array} } & {\begin{array}{*{20}c} {\begin{array}{*{20}c} 0 \\ { - {\text{ m}}\Omega ^{2} {\mathrm{D}}_{2} } \\ \end{array} } \\ 0 \\ 0 \\ \end{array} } & {\begin{array}{*{20}c} {\begin{array}{*{20}c} {\begin{array}{*{20}c} 0 \\ 0 \\ \end{array} } \\ { - \Omega ^{2} ({\mathrm{I}}_{{\mathrm{d}}} - {\mathrm{I}}_{{\mathrm{p}}} ){\mathrm{D}}_{3} } \\ 0 \\ \end{array} } & {\begin{array}{*{20}c} {\begin{array}{*{20}c} 0 \\ 0 \\ \end{array} } \\ 0 \\ { - \Omega ^{2} ({\mathrm{I}}_{{\mathrm{d}}} - {\mathrm{I}}_{{\mathrm{p}}} ){\mathrm{D}}_{4} } \\ \end{array} } \\ \end{array} } \\ \end{array} } \right]} \right. + \left[ {\begin{array}{*{20}c} {\begin{array}{*{20}c} {\begin{array}{*{20}c} {{\mathrm{k}}_{{{\mathrm{b}}_{\xi } }} {\mathrm{D}}_{1} } \\ 0 \\ \end{array} } \\ 0 \\ {{\mathrm{k}}_{{{\mathrm{b}}_{{\xi {\mathrm{c}}}} }} {\mathrm{D}}_{4} } \\ \end{array} } & {\begin{array}{*{20}c} {\begin{array}{*{20}c} 0 \\ {{\mathrm{k}}_{{{\mathrm{b}}_{\eta } }} {\mathrm{D}}_{2} } \\ \end{array} } \\ { - {\mathrm{k}}_{{{\mathrm{b}}_{{\eta {\mathrm{c}}}} }} {\mathrm{D}}_{3} } \\ 0 \\ \end{array} } & {\begin{array}{*{20}c} {\begin{array}{*{20}c} {\begin{array}{*{20}c} 0 \\ { - {\mathrm{k}}_{{{\mathrm{b}}_{{\eta {\mathrm{c}}}} }} {\mathrm{D}}_{2} } \\ \end{array} } \\ {{\mathrm{k}}_{{{\mathrm{b}}_{\alpha } }} {\mathrm{D}}_{3} } \\ 0 \\ \end{array} } & {\begin{array}{*{20}c} {\begin{array}{*{20}c} {{\mathrm{k}}_{{{\mathrm{b}}_{{\xi {\mathrm{c}}}} }} {\mathrm{D}}_{1} } \\ 0 \\ \end{array} } \\ 0 \\ {{\mathrm{k}}_{{{\mathrm{b}}_{\beta } }} {\mathrm{D}}_{4} } \\ \end{array} } \\ \end{array} } \\ \end{array} } \right] \hfill \\ + {\mathrm{K}}_{{\mathrm{S}}} \left. {\left[ {\begin{array}{*{20}c} {({\mathrm{q}}_{0}^{{\xi {\mathrm{t}}}} + {\mathrm{q}}_{1}^{{\xi {\mathrm{t}}}} {\mathrm{D}}^{\prime } {\text{ + q}}_{{\mathrm{2}}}^{{\xi {\mathrm{t}}}} {\mathrm{D}}^{{\prime 2}} )} \\ 0 \\ 0 \\ {{\mathrm{l}}\left( {{\mathrm{q}}_{0}^{{\xi {\mathrm{c}}}} + {\mathrm{q}}_{1}^{{\xi {\mathrm{c}}}} {\mathrm{D}}^{\prime } {\text{ + q}}_{{\mathrm{2}}}^{{\xi {\mathrm{c}}}} {\mathrm{D}}^{{\prime 2}} } \right)} \\ \end{array} \begin{array}{*{20}c} 0 \\ {({\mathrm{q}}_{0}^{{\eta {\mathrm{t}}}} + {\mathrm{q}}_{1}^{{\eta {\mathrm{t}}}} {\mathrm{D}}^{\prime } {\text{ + q}}_{{\mathrm{2}}}^{{\eta {\mathrm{t}}}} {\mathrm{D}}^{{\prime 2}} )} \\ 0 \\ {{\text{ - l}}\left( {{\mathrm{q}}_{0}^{{\eta {\mathrm{c}}}} + {\mathrm{q}}_{1}^{{\eta {\mathrm{c}}}} {\mathrm{D}}^{\prime } {\text{ + q}}_{{\mathrm{2}}}^{{\eta {\mathrm{c}}}} {\mathrm{D}}^{{\prime 2}} } \right)} \\ 0 \\ \end{array} \begin{array}{*{20}c} 0 \\ {{\text{ - l}}\left( {{\mathrm{q}}_{0}^{{\eta {\mathrm{c}}}} + {\mathrm{q}}_{1}^{{\eta {\mathrm{c}}}} {\mathrm{D}}^{\prime } {\text{ + q}}_{{\mathrm{2}}}^{{\eta {\mathrm{c}}}} {\mathrm{D}}^{{\prime 2}} } \right)} \\ {{\mathrm{l}}^{{\mathrm{2}}} \left( {{\mathrm{q}}_{0}^{{\eta {\mathrm{r}}}} + {\mathrm{q}}_{1}^{{\eta {\mathrm{r}}}} {\mathrm{D}}^{\prime } {\text{ + q}}_{{\mathrm{2}}}^{{\eta {\mathrm{r}}}} {\mathrm{D}}^{{\prime 2}} } \right)} \\ 0 \\ \end{array} \begin{array}{*{20}c} {{\mathrm{l}}\left( {{\mathrm{q}}_{0}^{{\xi {\mathrm{c}}}} + {\mathrm{q}}_{1}^{{\xi {\mathrm{c}}}} {\mathrm{D}}^{\prime } {\text{ + q}}_{{\mathrm{2}}}^{{\xi {\mathrm{c}}}} {\mathrm{D}}^{{\prime 2}} } \right)} \\ 0 \\ 0 \\ {{\mathrm{l}}^{{\mathrm{2}}} \left( {{\mathrm{q}}_{0}^{{\xi {\mathrm{r}}}} + {\mathrm{q}}_{1}^{{\xi {\mathrm{r}}}} {\mathrm{D}}^{\prime } {\text{ + q}}_{{\mathrm{2}}}^{{\xi {\mathrm{r}}}} {\mathrm{D}}^{{\prime 2}} } \right)} \\ \end{array} } \right]} \right)\left\{ {\begin{array}{*{20}c} \xi \\ \eta \\ \alpha \\ \beta \\ \end{array} } \right\} \hfill \\ = \left\{ {\begin{array}{*{20}c} {\begin{array}{*{20}c} {{\mathrm{D}}_{1} \left( {{\mathrm{F}}_{\xi }^{{{\mathrm{ma}}}} + {\mathrm{F}}_{\xi }^{{{\mathrm{un}}}} + {\mathrm{F}}_{\xi }^{{\mathrm{g}}} } \right)} \\ {{\mathrm{D}}_{2} \left( {{\mathrm{F}}_{\eta }^{{{\mathrm{ma}}}} + {\mathrm{F}}_{\eta }^{{{\mathrm{un}}}} + {\mathrm{F}}_{\eta }^{{\mathrm{g}}} } \right)} \\ \end{array} } \\ {{\mathrm{D}}_{3} \left( {{\mathrm{M}}_{\xi }^{{{\mathrm{ma}}}} + {\mathrm{M}}_{\xi }^{{\mathrm{T}}} } \right)} \\ {{\mathrm{D}}_{4} \left( {{\mathrm{M}}_{\eta }^{{{\mathrm{ma}}}} + {\mathrm{M}}_{\eta }^{{\mathrm{T}}} } \right)} \\ \end{array} } \right\} \hfill \\ \hfill \\ \end{gathered}$$

Stability of the system is obtained by setting all forcing terms to zero; therefore, from the above equation, the right-hand term will be removed, and the equation can be shown as

51 $$\begin{gathered} \left[ {\begin{array}{*{20}c} {\begin{array}{*{20}c} {{\mathrm{mD}}_{1} } \\ 0 \\ {\begin{array}{*{20}c} 0 \\ 0 \\ \end{array} } \\ \end{array} } & {\begin{array}{*{20}c} {\begin{array}{*{20}c} 0 \\ {{\mathrm{mD}}_{2} } \\ \end{array} } \\ 0 \\ 0 \\ \end{array} } & {\begin{array}{*{20}c} {\begin{array}{*{20}c} {\begin{array}{*{20}c} 0 \\ 0 \\ \end{array} } \\ {{\mathrm{I}}_{{\mathrm{d}}} {\mathrm{D}}_{3} } \\ 0 \\ \end{array} } & {\begin{array}{*{20}c} {\begin{array}{*{20}c} 0 \\ 0 \\ \end{array} } \\ 0 \\ {{\mathrm{I}}_{{\mathrm{d}}} {\mathrm{D}}_{4} } \\ \end{array} } \\ \end{array} } \\ \end{array} } \right]\left\{ {\begin{array}{*{20}c} {\begin{array}{*{20}c} {\ddot{\upxi }} \\ {\ddot{\upeta }} \\ \end{array} } \\ {\ddot{\upalpha }} \\ {\ddot{\upbeta }} \\ \end{array} } \right\} \hfill \\ + \left( {\left[ {\begin{array}{*{20}c} {\begin{array}{*{20}c} 0 \\ { - 2{\mathrm{m}}\Omega {\mathrm{D}}_{1} } \\ {\begin{array}{*{20}c} 0 \\ 0 \\ \end{array} } \\ \end{array} } & {\begin{array}{*{20}c} {\begin{array}{*{20}c} { - 2{\mathrm{m}}\Omega {\mathrm{D}}_{2} } \\ 0 \\ \end{array} } \\ 0 \\ 0 \\ \end{array} } & {\begin{array}{*{20}c} {\begin{array}{*{20}c} {\begin{array}{*{20}c} 0 \\ 0 \\ \end{array} } \\ 0 \\ {\Omega (2{\mathrm{I}}_{{\mathrm{d}}} - {\mathrm{I}}_{{\mathrm{p}}} ){\mathrm{D}}_{3} } \\ \end{array} } & {\begin{array}{*{20}c} {\begin{array}{*{20}c} 0 \\ 0 \\ \end{array} } \\ {\Omega ( - 2{\mathrm{I}}_{{\mathrm{d}}} + {\mathrm{I}}_{{\mathrm{p}}} ){\mathrm{D}}_{4} } \\ 0 \\ \end{array} } \\ \end{array} } \\ \end{array} } \right]} \right. \hfill \\ + \left. {\left[ {\begin{array}{*{20}c} {\begin{array}{*{20}c} {\begin{array}{*{20}c} {{\mathrm{c}}_{{{\mathrm{b}}_{\upxi } }} {\mathrm{D}}_{1} } \\ 0 \\ \end{array} } \\ 0 \\ {{\mathrm{c}}_{{{\mathrm{b}}_{{\upxi {\mathrm{c}}}} }} {\mathrm{D}}_{1} } \\ \end{array} } & {\begin{array}{*{20}c} {\begin{array}{*{20}c} 0 \\ {{\mathrm{c}}_{{{\mathrm{b}}_{\upeta } }} {\mathrm{D}}_{2} } \\ \end{array} } \\ { - {\mathrm{c}}_{{{\mathrm{b}}_{{\upeta {\mathrm{c}}}} }} {\mathrm{D}}_{2} } \\ 0 \\ \end{array} } & {\begin{array}{*{20}c} {\begin{array}{*{20}c} {\begin{array}{*{20}c} 0 \\ { - {\mathrm{c}}_{{{\mathrm{b}}_{{\upeta {\mathrm{c}}}} }} {\mathrm{D}}_{3} } \\ \end{array} } \\ {{\mathrm{c}}_{{{\mathrm{b}}_{\upalpha } }} {\mathrm{D}}_{3} } \\ 0 \\ \end{array} } & {\begin{array}{*{20}c} {\begin{array}{*{20}c} {{\mathrm{c}}_{{{\mathrm{b}}_{{\upxi {\mathrm{c}}}} }} {\mathrm{D}}_{4} } \\ 0 \\ \end{array} } \\ 0 \\ {{\mathrm{c}}_{{{\mathrm{b}}_{\upbeta } }} {\mathrm{D}}_{4} } \\ \end{array} } \\ \end{array} } \\ \end{array} } \right]} \right)\left\{ {\begin{array}{*{20}c} {\begin{array}{*{20}c} {\dot{\upxi }} \\ {\dot{\upeta }} \\ \end{array} } \\ {\dot{\upalpha }} \\ {\dot{\upbeta }} \\ \end{array} } \right\} \hfill \\ + \left( {\left[ {\begin{array}{*{20}c} { - {\text{ m}}\Omega ^{2} {\mathrm{D}}_{1} } & 0 & 0 & 0 \\ 0 & { - {\text{ m}}\Omega ^{2} {\mathrm{D}}_{2} } & 0 & 0 \\ 0 & 0 & { - \Omega ^{2} ({\mathrm{I}}_{{\mathrm{d}}} - {\mathrm{I}}_{{\mathrm{p}}} ){\mathrm{D}}_{3} } & 0 \\ 0 & 0 & 0 & { - \Omega ^{2} ({\mathrm{I}}_{{\mathrm{d}}} - {\mathrm{I}}_{{\mathrm{p}}} ){\mathrm{D}}_{4} } \\ \end{array} } \right]} \right. + \left[ {\begin{array}{*{20}c} {\begin{array}{*{20}c} {\begin{array}{*{20}c} {{\mathrm{k}}_{{{\mathrm{b}}_{\upxi } }} {\mathrm{D}}_{1} } \\ 0 \\ \end{array} } \\ 0 \\ {{\mathrm{k}}_{{{\mathrm{b}}_{{\upxi {\mathrm{c}}}} }} {\mathrm{D}}_{1} } \\ \end{array} } & {\begin{array}{*{20}c} {\begin{array}{*{20}c} 0 \\ {{\mathrm{k}}_{{{\mathrm{b}}_{\upeta } }} {\mathrm{D}}_{2} } \\ \end{array} } \\ { - {\mathrm{k}}_{{{\mathrm{b}}_{{\upeta {\mathrm{c}}}} }} {\mathrm{D}}_{2} } \\ 0 \\ \end{array} } & {\begin{array}{*{20}c} {\begin{array}{*{20}c} {\begin{array}{*{20}c} 0 \\ { - {\mathrm{k}}_{{{\mathrm{b}}_{{\upeta {\mathrm{c}}}} }} {\mathrm{D}}_{3} } \\ \end{array} } \\ {{\mathrm{k}}_{{{\mathrm{b}}_{\upalpha } }} {\mathrm{D}}_{3} } \\ 0 \\ \end{array} } & {\begin{array}{*{20}c} {\begin{array}{*{20}c} {{\mathrm{k}}_{{{\mathrm{b}}_{{\upxi {\mathrm{c}}}} }} {\mathrm{D}}_{4} } \\ 0 \\ \end{array} } \\ 0 \\ {{\mathrm{k}}_{{{\mathrm{b}}_{\upbeta } }} {\mathrm{D}}_{4} } \\ \end{array} } \\ \end{array} } \\ \end{array} } \right] \hfill \\ + {\mathrm{K}}_{{\mathrm{S}}} \left. {\left[ {\begin{array}{*{20}c} {\left( {{\mathrm{q}}_{0}^{{\upxi {\mathrm{t}}}} + {\mathrm{q}}_{1}^{{\upxi {\mathrm{t}}}} {\mathrm{D}}^{\prime } {\text{ + q}}_{2}^{{\upxi {\mathrm{t}}}} {\mathrm{D}}^{{\prime 2}} } \right)} \\ 0 \\ 0 \\ {{\mathrm{l}}\left( {{\mathrm{q}}_{0}^{{\upxi {\mathrm{c}}}} + {\mathrm{q}}_{1}^{{\upxi {\mathrm{c}}}} {\mathrm{D}}^{\prime } {\text{ + q}}_{2}^{{\upxi {\mathrm{c}}}} {\mathrm{D}}^{{\prime 2}} } \right)} \\ \end{array} \begin{array}{*{20}c} 0 \\ {\left( {{\mathrm{q}}_{0}^{{\eta {\mathrm{t}}}} + {\mathrm{q}}_{1}^{{\upeta {\mathrm{t}}}} {\mathrm{D}}^{\prime } {\text{ + q}}_{2}^{{\upeta {\mathrm{t}}}} {\mathrm{D}}^{{\prime 2}} } \right)} \\ 0 \\ {{\text{ - l}}\left( {{\mathrm{q}}_{0}^{{\upeta {\mathrm{c}}}} + {\mathrm{q}}_{1}^{{\upeta {\mathrm{c}}}} {\mathrm{D}}^{\prime } {\text{ + q}}_{2}^{{\upeta {\mathrm{c}}}} {\mathrm{D}}^{{\prime 2}} } \right)} \\ 0 \\ \end{array} \begin{array}{*{20}c} 0 \\ {{\text{ - l}}\left( {{\mathrm{q}}_{0}^{{\upeta {\mathrm{c}}}} + {\mathrm{q}}_{1}^{{\upeta {\mathrm{c}}}} {\mathrm{D}}^{\prime } {\text{ + q}}_{2}^{{\upeta {\mathrm{c}}}} {\mathrm{D}}^{{\prime 2}} } \right)} \\ {{\mathrm{l}}^{2} \left( {{\mathrm{q}}_{0}^{{\upeta {\mathrm{r}}}} + {\mathrm{q}}_{1}^{{\upeta {\mathrm{r}}}} {\mathrm{D}}^{\prime } {\text{ + q}}_{2}^{{\upeta {\mathrm{r}}}} {\mathrm{D}}^{{\prime 2}} } \right)} \\ 0 \\ \end{array} \begin{array}{*{20}c} {{\mathrm{l}}\left( {{\mathrm{q}}_{0}^{{\upxi {\mathrm{c}}}} + {\mathrm{q}}_{1}^{{\upxi {\mathrm{c}}}} {\mathrm{D}}^{\prime } {\text{ + q}}_{2}^{{\upxi {\mathrm{c}}}} {\mathrm{D}}^{{\prime 2}} } \right)} \\ 0 \\ 0 \\ {{\mathrm{l}}^{2} \left( {{\mathrm{q}}_{0}^{{\upxi {\mathrm{r}}}} + {\mathrm{q}}_{1}^{{\upxi {\mathrm{r}}}} {\mathrm{D}}^{\prime } {\text{ + q}}_{2}^{{\upxi {\mathrm{r}}}} {\mathrm{D}}^{{\prime 2}} } \right)} \\ \end{array} } \right]} \right)\left\{ {\begin{array}{*{20}c} \upxi \\ \upeta \\ \upalpha \\ \upbeta \\ \end{array} } \right\} \hfill \\ = 0 \hfill \\ \hfill \\ \end{gathered}$$

Operating the above equations by the net denominator polynomial in D’ for converting them to simultaneous differential equations, putting the solutions in the form $\upxi ={\mathrm{X}}_{1}{\mathrm{e}}^{\mathrm{iwt}}$, ${\upeta =\mathrm{X}}_{2}{\mathrm{e}}^{\mathrm{iwt}}$, $\alpha = {\mathrm{X}}_{3} {\mathrm{e}}^{{{\mathrm{iwt}}}}$ and $\upbeta ={\mathrm{X}}_{4}{\mathrm{e}}^{\mathrm{iwt}}$, and c, the equations are written again as in equations 52-55. These may be put using the matrix notation as in equation 52 shown below

52 $$\left[ {\begin{array}{*{20}c} {{\mathrm{B}}_{1} } & {{\mathrm{B}}_{2} } & 0 & {{\mathrm{B}}_{3} } \\ {{\mathrm{B}}_{4} } & {{\mathrm{B}}_{5} } & {{\mathrm{B}}_{6} } & 0 \\ 0 & {{\mathrm{B}}_{7} } & {{\mathrm{B}}_{8} } & {{\mathrm{B}}_{9} } \\ {{\mathrm{B}}_{10} } & 0 & {{\mathrm{B}}_{11} } & {{\mathrm{B}}_{12} } \\ \end{array} } \right]\left[ {\begin{array}{*{20}c} {{\mathrm{X}}_{1} } \\ {{\mathrm{X}}_{2} } \\ {{\mathrm{X}}_{3}^{\prime} } \\ {{\mathrm{X}}_{4}^{\prime} } \\ \end{array} } \right] = 0$$

Substituting ${\mathrm{X}}_{3}$’ = l X_3_ and X_4_’ = l X_4_ and separating coefficients of X_1_, X_2_, X_3_’ and X_4_’

Where,

$$\begin{gathered} {\mathrm{B}}_{1} = \left( {{\mathrm{q}}_{0}^{{\upxi {\mathrm{t}}}} + \updelta \left( {{\mathrm{iq}}_{1}^{{\upxi {\mathrm{t}}}} - {\mathrm{q}}_{2}^{{\upxi {\mathrm{t}}}} \updelta } \right)} \right)\left( {{\mathrm{r}}_{0}^{{\upxi {\mathrm{c}}}} + \updelta \left( {{\mathrm{ir}}_{1}^{{\upxi {\mathrm{c}}}} - {\mathrm{r}}_{2}^{{\upxi {\mathrm{c}}}} \updelta } \right)} \right) \hfill \\ + \left( {{\mathrm{r}}_{0}^{{\upxi {\mathrm{t}}}} + \updelta \left( {{\mathrm{ir}}_{1}^{{\upxi {\mathrm{t}}}} - {\mathrm{r}}_{2}^{{\upxi {\mathrm{t}}}} \updelta } \right)} \right)\left( { - {\mathrm{r}}_{0}^{{\upxi {\mathrm{c}}}} + \updelta \left( { - {\mathrm{ir}}_{1}^{{\upxi {\mathrm{c}}}} + {\mathrm{r}}_{2}^{{\upxi {\mathrm{c}}}} \updelta } \right)} \right)\left( {\left( {\updelta ^{2} + \updelta _{{\mathrm{r}}}^{2} } \right)} \right); \hfill \\ \end{gathered}$$

$${\mathrm{B}}_{2} = \left( {{\mathrm{r}}_{0}^{{\upxi {\mathrm{t}}}} + \updelta \left( {{\mathrm{ir}}_{1}^{{\upxi {\mathrm{t}}}} - {\mathrm{r}}_{2}^{{\upxi {\mathrm{t}}}} \updelta } \right)} \right)\left( { - {\mathrm{r}}_{0}^{{\upxi {\mathrm{c}}}} + \updelta \left( { - {\mathrm{ir}}_{1}^{{\upxi {\mathrm{c}}}} + {\mathrm{r}}_{2}^{{\upxi {\mathrm{c}}}} \updelta } \right)} \right)\left( {2{\mathrm{i}}\updelta \updelta_{{\mathrm{r}}} } \right);$$

$${\mathrm{B}}_{3} = \left( {{\mathrm{q}}_{0}^{{\upxi {\mathrm{c}}}} + \updelta \left( {{\mathrm{iq}}_{1}^{{\upxi {\mathrm{c}}}} - {\mathrm{q}}_{2}^{{\upxi {\mathrm{c}}}} \updelta } \right)} \right)\left( { - {\mathrm{r}}_{0}^{{\upxi {\mathrm{t}}}} \updelta \left( { - {\mathrm{ir}}_{1}^{{\upxi {\mathrm{t}}}} + {\mathrm{r}}_{2}^{{\upxi {\mathrm{t}}}} \updelta } \right)} \right);$$

$${\mathrm{B}}_{4} = \left( {{\mathrm{r}}_{0}^{{\upeta {\mathrm{t}}}} + \updelta \left( {{\mathrm{ir}}_{1}^{{\upeta {\mathrm{t}}}} - {\mathrm{r}}_{2}^{{\upeta {\mathrm{t}}}} \updelta } \right)} \right)\left( { - {\mathrm{r}}_{0}^{{\upeta {\mathrm{c}}}} + \updelta \left( { - {\mathrm{ir}}_{1}^{{\upeta {\mathrm{c}}}} + {\mathrm{r}}_{2}^{{\upeta {\mathrm{c}}}} \updelta } \right)} \right)\left( { - 2{\mathrm{i}}\updelta \updelta_{{\mathrm{r}}} } \right);$$

$$\begin{gathered} {\mathrm{B}}_{5} = \left( {{\mathrm{q}}_{0}^{{\upeta {\mathrm{t}}}} + \updelta \left( {{\mathrm{iq}}_{1}^{{\upeta {\mathrm{t}}}} - {\mathrm{q}}_{2}^{{\upeta {\mathrm{t}}}} \updelta } \right)} \right)\left( {{\mathrm{r}}_{0}^{{\upeta {\mathrm{c}}}} + \updelta \left( {{\mathrm{ir}}_{1}^{{\upeta {\mathrm{c}}}} - {\mathrm{r}}_{2}^{{\upeta {\mathrm{c}}}} \updelta } \right)} \right) \hfill \\ + ({\mathrm{r}}_{0}^{{\upeta {\mathrm{t}}}} + \updelta ({\mathrm{ir}}_{1}^{{\upeta {\mathrm{t}}}} - {\mathrm{r}}_{2}^{{\upeta {\mathrm{t}}}} \updelta ))( - {\mathrm{r}}_{0}^{{\upeta {\mathrm{c}}}} + \updelta ( - {\mathrm{ir}}_{1}^{{\upeta {\mathrm{c}}}} + {\mathrm{r}}_{2}^{{\upeta {\mathrm{c}}}} \updelta ))(\left( {\updelta ^{2} + \updelta _{{\mathrm{r}}}^{2} } \right)); \hfill \\ \end{gathered}$$

$${\mathrm{B}}_{6} = \left( {{\mathrm{q}}_{0}^{{\upeta {\mathrm{c}}}} + \updelta \left( {{\mathrm{iq}}_{1}^{{\upeta {\mathrm{c}}}} - {\mathrm{q}}_{2}^{{\upeta {\mathrm{c}}}} \updelta } \right)} \right)\left( {{\mathrm{r}}_{0}^{{\upeta {\mathrm{t}}}} + \updelta \left( {{\mathrm{ir}}_{1}^{{\upeta {\mathrm{t}}}} - {\mathrm{r}}_{2}^{{\upeta {\mathrm{t}}}} \updelta } \right)} \right);$$

$${\mathrm{B}}_{7} = ({\mathrm{q}}_{0}^{{\upeta {\mathrm{c}}}} + \updelta ({\mathrm{iq}}_{1}^{{\upeta {\mathrm{c}}}} - {\mathrm{q}}_{2}^{{\upeta {\mathrm{c}}}} \updelta ))({\mathrm{r}}_{0}^{{\upeta {\mathrm{r}}}} + \updelta ({\mathrm{ir}}_{1}^{{\upeta {\mathrm{r}}}} - {\mathrm{r}}_{2}^{{\upeta {\mathrm{r}}}} \updelta ));$$

$$\begin{gathered} {\mathrm{B}}_{8} = ({\mathrm{q}}_{0}^{{\upeta {\mathrm{r}}}} + \updelta ({\mathrm{iq}}_{1}^{{\upeta {\mathrm{r}}}} - {\mathrm{q}}_{2}^{{\upeta {\mathrm{r}}}} \updelta ))({\mathrm{r}}_{0}^{{\upeta {\mathrm{c}}}} + \updelta ({\mathrm{ir}}_{1}^{{\upeta {\mathrm{c}}}} - {\mathrm{r}}_{2}^{{\upeta {\mathrm{c}}}} \updelta )) + \frac{1}{{\mathrm{R}}}{\mathrm{G}}_{0}^{2} ({\mathrm{r}}_{0}^{{\upeta {\mathrm{c}}}} \hfill \\ + \updelta ({\mathrm{ir}}_{1}^{{\upeta {\mathrm{c}}}} - {\mathrm{r}}_{2}^{{\upeta {\mathrm{c}}}} \updelta ))( - {\mathrm{r}}_{0}^{{\upeta {\mathrm{r}}}} + \updelta ( - {\mathrm{ir}}_{1}^{{\upeta {\mathrm{r}}}} + {\mathrm{r}}_{2}^{{\upeta {\mathrm{r}}}} \updelta ))((\updelta ^{2} - ( - 1 + {\mathrm{R}})\updelta _{{\mathrm{r}}}^{2} )); \hfill \\ \end{gathered}$$

$${\mathrm{B}}_{9} = \frac{1}{{\mathrm{R}}}{\mathrm{G}}_{0}^{2} ({\mathrm{r}}_{0}^{{\upeta {\mathrm{c}}}} + \updelta ({\mathrm{ir}}_{1}^{{\upeta {\mathrm{c}}}} - {\mathrm{r}}_{2}^{{\upeta {\mathrm{c}}}} \updelta ))( - {\mathrm{r}}_{0}^{{\upeta {\mathrm{r}}}} + \updelta ( - {\mathrm{ir}}_{1}^{{\upeta {\mathrm{r}}}} + {\mathrm{r}}_{2}^{{\upeta {\mathrm{r}}}} \updelta ))( - {\mathrm{i}}( - 2 + {\mathrm{R}})\updelta \updelta_{{\mathrm{r}}} );$$

$${\mathrm{B}}_{10} = \left( {{\mathrm{q}}_{0}^{{\upxi {\mathrm{c}}}} + \updelta \left( {{\mathrm{iq}}_{1}^{{\upxi {\mathrm{c}}}} - {\mathrm{q}}_{2}^{{\upxi {\mathrm{c}}}} \updelta } \right)} \right)\left( { - {\mathrm{r}}_{0}^{{\upxi {\mathrm{r}}}} + \updelta \left( { - {\mathrm{ir}}_{1}^{{\upxi {\mathrm{r}}}} + {\mathrm{r}}_{2}^{{\upxi {\mathrm{r}}}} \updelta } \right)} \right);$$

$${\mathrm{B}}_{11} = \frac{1}{{\mathrm{R}}}{\mathrm{G}}_{0}^{2} ({\mathrm{r}}_{0}^{{\upxi {\mathrm{c}}}} + \updelta ({\mathrm{ir}}_{1}^{{\upxi {\mathrm{c}}}} - {\mathrm{r}}_{2}^{{\upxi {\mathrm{c}}}} \updelta ))( - {\mathrm{r}}_{0}^{{\upxi {\mathrm{r}}}} + \updelta ( - {\mathrm{ir}}_{1}^{{\upxi {\mathrm{r}}}} + {\mathrm{r}}_{2}^{{\upxi {\mathrm{r}}}} \updelta ))({\mathrm{i}}\left( { - 2 + {\mathrm{R}}} \right)\updelta \updelta_{{\mathrm{r}}} );$$

$$\begin{gathered} {\mathrm{B}}_{{12}} = \left( {{\mathrm{q}}_{0}^{{\upxi {\mathrm{r}}}} + \updelta \left( {{\mathrm{iq}}_{1}^{{\upxi {\mathrm{r}}}} - {\mathrm{q}}_{2}^{{\upxi {\mathrm{r}}}} \updelta } \right)} \right)\left( {{\mathrm{r}}_{0}^{{\upxi {\mathrm{c}}}} + \updelta \left( {{\mathrm{ir}}_{1}^{{\upxi {\mathrm{c}}}} - {\mathrm{r}}_{2}^{{\upxi {\mathrm{c}}}} \updelta } \right)} \right) + \frac{1}{{\mathrm{R}}}{\mathrm{G}}_{0}^{2} ({\mathrm{r}}_{0}^{{\upxi {\mathrm{c}}}} + \updelta ({\mathrm{ir}}_{1}^{{\upxi {\mathrm{c}}}} - {\mathrm{r}}_{2}^{{\upxi {\mathrm{c}}}} \updelta ))( - {\mathrm{r}}_{0}^{{\upxi {\mathrm{r}}}} + \hfill \\ \updelta ( - {\mathrm{ir}}_{1}^{{\upxi {\mathrm{r}}}} + {\mathrm{r}}_{2}^{{\upxi {\mathrm{r}}}} \updelta ))(\left( {\updelta ^{2} - \left( { - 1 + {\mathrm{R}}} \right)\updelta _{{\mathrm{r}}}^{2} } \right)); \hfill \\ \end{gathered}$$

The characteristic equation is obtained by finding the determinant of $\left[\mathrm{B}\right]$ matrix and equating it to zero. Roots (w, in this case) of the equation are obtained, which are in the form $(\mathrm{a}+\mathrm{ib})$. Since the solution assumed was in the form of ${\mathrm{Xe}}^{\mathrm{iwt}}$, speed at which the imaginary part of the root becomes negative, the system becomes unstable and gives the value of the stability limit speed.

### Response

The response to forces and moments is calculated using the equation of motion in Eq. (48).

#### Gravity

The force due to gravity is static in the stationary frame and appears to be rotating in the spin-synchronized rotary frame of the rotor.

53 $${\mathrm{F}}_{{{\upxi }}}^{{\mathrm{g}}} = - {\mathrm{mg}}\sin \left( {{\Omega \mathrm{t}}} \right)\,{\mathrm{and}}\,{\mathrm{F}}_{{{\upeta }}}^{{\mathrm{g}}} = - {\mathrm{mg}}\cos \left( {{\Omega \mathrm{t}}} \right)$$

#### Unbalance

**Fig. 13:**
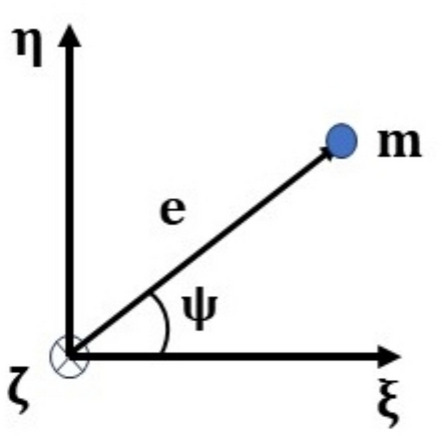
Unbalanced location in ξ-η plane.

Unbalanced force rotates with the rotor at its spin speed; therefore, it appears as a static force in the rotary frame of the rotor. Let the unbalance be located at a distance e and at an angle ψ as shown in Fig. 13, then

54 $${\mathrm{F}}_{\upxi }^{{{\mathrm{un}}}} = {\mathrm{me}}\Omega^{2} \cos \left( \uppsi \right){\kern 1pt} {\kern 1pt} {\mathrm{and}}{\kern 1pt} {\kern 1pt} {\mathrm{F}}_{\upeta }^{{{\mathrm{un}}}} = {\mathrm{me}}\Omega^{2} \sin \left( \uppsi \right)$$

#### Misalignment

Misalignment present between the two ends of the shaft (to be joined by the coupling) is constant in the stationary frame, but the coupling stiffness matrix obtained in the above analysis is rotating with the spin synchronized rotary frame. Thus, the components of deflection in the stationary frame due to misalignment are first converted into components in the rotary frame, and the forces and moments due to misalignment are computed at the coupling end. Finally, these forces and moments are transferred to the disc end, and the response is calculated.

Let ${\mathrm{v}}_{\mathrm{x}}$ and ${\mathrm{v}}_{\mathrm{y}}$ be the amount of parallel misalignment along and $\upalpha_{{\mathrm{x}}}$ and $\upalpha_{{\mathrm{y}}}$ be the amount of angular misalignment about the stationary x and y axes, respectively. Then the corresponding values of misalignments in the rotating frame are given as

55 $$\left[ {\begin{array}{*{20}c} {{\mathrm{v}}_{\upxi } } \\ {{\mathrm{v}}_{\upeta } } \\ {\upalpha_{\upxi } } \\ {\upalpha_{\upeta } } \\ \end{array} } \right] = {\mathrm{T}}_{1} \left[ {\begin{array}{*{20}c} {{\mathrm{v}}_{{\mathrm{x}}} } \\ {{\mathrm{v}}_{{\mathrm{y}}} } \\ {\upalpha_{{\mathrm{x}}} } \\ {\upalpha_{{\mathrm{y}}} } \\ \end{array} } \right],{\kern 1pt} \;{\mathrm{here}}\;{\mathrm{T}}_{1} = \left[ {\begin{array}{*{20}c} {\mathrm{R}} & 0 \\ 0 & {\mathrm{R}} \\ \end{array} } \right]$$

And, the rotation matrix,$$\mathrm{R} = \left[ {\begin{array}{*{20}c} {\cos \left( {\Omega \mathrm{t}} \right)} & { - \sin \left( {\Omega \mathrm{t}} \right)} \\ {\sin \left( {\Omega \mathrm{t}} \right)} & {\cos \left( {\Omega \mathrm{t}} \right)} \\ \end{array} } \right]$$

In the above equation, the misalignment vector has components ${\mathrm{v}}_{\upxi }$, ${\mathrm{v}}_{\upeta }$ for parallel and $\upalpha_{\upxi }$, $\upalpha_{\upeta }$ for angular misalignment, respectively. Forces and moments due to misalignment, as derived in Section "Calculation of coupling stiffness and damping", are written at the disc end as

56 $${\mathrm{F}}_{\upxi }^{{{\mathrm{ma}}}} = {\mathrm{kv}}_{\upxi } {\kern 1pt} {\mathrm{and}}\;{\mathrm{F}}_{\upeta }^{{{\mathrm{ma}}}} = {\mathrm{kv}}_{\upeta }$$

57 $${\mathrm{M}}_{\upxi }^{{{\mathrm{ma}}}} = {\text{ k}}_{{\mathrm{b}}} \upalpha_{\upxi } + {\text{ kv}}_{\upeta } \left( {{\mathrm{a}} + {\mathrm{a}}_{1} } \right){\text{ and M}}_{\upeta }^{{{\mathrm{ma}}}} = {\text{ k}}_{{\mathrm{b}}} \upalpha_{\upeta } - {\mathrm{kv}}_{\upxi } \left( {{\mathrm{a}} + {\mathrm{a}}_{1} } \right)$$

Here, a+a_1_ is the distance of the coupling end from the disc centre (Fig. 9).

#### Torque

Let $\mathrm{T}$ be the torque transmitted in the above misaligned condition. The moments produced due to this torque, as calculated in equations (35-43), are represented as

58 $${\mathrm{M}}_{\upxi }^{{\mathrm{T}}} = \frac{{\mathrm{T}}}{{{\mathrm{l}}_{{\mathrm{h}}} }}{\mathrm{v}}_{\upxi } - {\mathrm{T}}\upalpha_{\upxi } \;{\mathrm{and}}\;{\mathrm{M}}_{\upeta }^{{\mathrm{T}}} = \frac{{\mathrm{T}}}{{{\mathrm{l}}_{{\mathrm{h}}} }}{\mathrm{v}}_{\upxi } + {\mathrm{T}}\upalpha_{\upxi }$$

The left-hand side of the equations of motion is the same as in (44-47). Operating the net denominator term (obtained from the left-hand side of equations) on the right-side force terms, forcing terms are obtained as:

59 $$\begin{gathered} \left[ {\begin{array}{*{20}c} {{\mathrm{mD}}_{{\mathrm{1}}} } & 0 & 0 & 0 \\ 0 & {{\mathrm{mD}}_{2} } & 0 & 0 \\ 0 & 0 & {{\mathrm{I}}_{{\mathrm{d}}} {\mathrm{D}}_{{\mathrm{3}}} } & 0 \\ 0 & 0 & 0 & {{\mathrm{I}}_{{\mathrm{d}}} {\mathrm{D}}_{{\mathrm{4}}} } \\ \end{array} } \right]\left\{ {\begin{array}{*{20}c} {\ddot{\upxi }} \\ {\ddot{\upeta }} \\ {\ddot{\upalpha }} \\ {\ddot{\upbeta }} \\ \end{array} } \right\} \hfill \\ + \left( {\left[ {\begin{array}{*{20}c} 0 & { - 2{\mathrm{m}}\Omega {\mathrm{D}}_{{\mathrm{1}}} } & 0 & 0 \\ { - 2{\mathrm{m}}\Omega {\mathrm{D}}_{{\mathrm{2}}} } & 0 & 0 & 0 \\ 0 & 0 & 0 & {\Omega (2{\mathrm{I}}_{{\mathrm{d}}} {\text{ + I}}_{{\mathrm{p}}} ){\mathrm{D}}_{3} } \\ 0 & 0 & {\Omega (2{\mathrm{I}}_{{\mathrm{d}}} - {\mathrm{I}}_{{\mathrm{p}}} ){\mathrm{D}}_{4} } & 0 \\ \end{array} } \right] + \left[ {\begin{array}{*{20}c} {{\mathrm{c}}_{{{\mathrm{b}}_{\upxi } }} {\mathrm{D}}_{1} } & 0 & 0 & {{\mathrm{c}}_{{{\mathrm{b}}_{{\upxi {\mathrm{c}}}} }} {\mathrm{D}}_{1} } \\ 0 & {{\mathrm{c}}_{{{\mathrm{b}}_{\upeta } }} {\mathrm{D}}_{2} } & { - {\mathrm{c}}_{{{\mathrm{b}}_{{\upeta {\mathrm{c}}}} }} {\mathrm{D}}_{2} } & 0 \\ 0 & { - {\mathrm{c}}_{{{\mathrm{b}}_{{\upeta {\mathrm{c}}}} }} {\mathrm{D}}_{3} } & {{\mathrm{c}}_{{{\mathrm{b}}_{\upalpha } }} {\mathrm{D}}_{3} } & 0 \\ {{\mathrm{c}}_{{{\mathrm{b}}_{{\upxi {\mathrm{c}}}} }} {\mathrm{D}}_{4} } & 0 & 0 & {{\mathrm{c}}_{{{\mathrm{b}}_{\upbeta } }} {\mathrm{D}}_{4} } \\ \end{array} } \right]} \right)\left\{ {\begin{array}{*{20}c} {\dot{\upxi }} \\ {\dot{\upeta }} \\ {\dot{\upalpha }} \\ {\dot{\upbeta }} \\ \end{array} } \right\} \hfill \\ + \left( {\left[ {\begin{array}{*{20}c} { - {\text{ m}}\Omega ^{2} {\mathrm{D}}_{1} } & 0 & 0 & 0 \\ 0 & { - {\text{ m}}\Omega ^{2} {\mathrm{D}}_{2} } & 0 & 0 \\ 0 & 0 & { - \Omega ^{2} ({\mathrm{I}}_{{\mathrm{d}}} - {\mathrm{I}}_{{\mathrm{p}}} ){\mathrm{D}}_{3} } & 0 \\ 0 & 0 & 0 & { - \Omega ^{2} ({\mathrm{I}}_{{\mathrm{d}}} - {\mathrm{I}}_{{\mathrm{p}}} ){\mathrm{D}}_{4} } \\ \end{array} } \right]} \right. + \left[ {\begin{array}{*{20}c} {{\mathrm{k}}_{{{\mathrm{b}}_{\upxi } }} {\mathrm{D}}_{1} } & 0 & 0 & {{\mathrm{k}}_{{{\mathrm{b}}_{{\upxi {\mathrm{c}}}} }} {\mathrm{D}}_{1} } \\ 0 & {{\mathrm{k}}_{{{\mathrm{b}}_{\upeta } }} {\mathrm{D}}_{2} } & { - {\mathrm{k}}_{{{\mathrm{b}}_{{\upeta {\mathrm{c}}}} }} {\mathrm{D}}_{2} } & 0 \\ 0 & { - {\mathrm{k}}_{{{\mathrm{b}}_{{\upeta {\mathrm{c}}}} }} {\mathrm{D}}_{3} } & {{\mathrm{k}}_{{{\mathrm{b}}_{\upalpha } }} {\mathrm{D}}_{3} } & 0 \\ {{\mathrm{k}}_{{{\mathrm{b}}_{{\upxi {\mathrm{c}}}} }} {\mathrm{D}}_{4} } & 0 & 0 & {{\mathrm{k}}_{{{\mathrm{b}}_{\upbeta } }} {\mathrm{D}}_{4} } \\ \end{array} } \right] \hfill \\ + {\mathrm{K}}_{{\mathrm{S}}} \left[ {\begin{array}{*{20}c} {{\mathrm{(q}}_{0}^{{\upxi {\mathrm{t}}}} + {\mathrm{q}}_{1}^{{\upxi {\mathrm{t}}}} {\mathrm{D}}^{\prime } {\text{ + q}}_{{\mathrm{2}}}^{{\upxi {\mathrm{t}}}} {\mathrm{D}}^{{\prime 2}} )} \\ 0 \\ 0 \\ {{\mathrm{l}}\left( {{\mathrm{q}}_{0}^{{\upxi {\mathrm{c}}}} + {\mathrm{q}}_{1}^{{\upxi {\mathrm{c}}}} {\mathrm{D}}^{\prime } {\text{ + q}}_{{\mathrm{2}}}^{{\upxi {\mathrm{c}}}} {\mathrm{D}}^{{\prime 2}} } \right)} \\ \end{array} \begin{array}{*{20}c} 0 \\ {({\mathrm{q}}_{0}^{{\upeta {\mathrm{t}}}} + {\mathrm{q}}_{1}^{{\upeta {\mathrm{t}}}} {\mathrm{D}}^{\prime } {\text{ + q}}_{{\mathrm{2}}}^{{\upeta {\mathrm{t}}}} {\mathrm{D}}^{{\prime 2}} )} \\ 0 \\ {{\text{ - l}}\left( {{\mathrm{q}}_{0}^{{\upeta {\mathrm{c}}}} + {\mathrm{q}}_{1}^{{\upeta {\mathrm{c}}}} {\mathrm{D}}^{\prime } {\text{ + q}}_{{\mathrm{2}}}^{{\upeta {\mathrm{c}}}} {\mathrm{D}}^{{\prime 2}} } \right)} \\ 0 \\ \end{array} \begin{array}{*{20}c} 0 \\ {{\text{ - l}}\left( {{\mathrm{q}}_{0}^{{\upeta {\mathrm{c}}}} + {\mathrm{q}}_{1}^{{\upeta {\mathrm{c}}}} {\mathrm{D}}^{\prime } {\text{ + q}}_{{\mathrm{2}}}^{{\upeta {\mathrm{c}}}} {\mathrm{D}}^{{\prime 2}} } \right)} \\ {{\mathrm{l}}^{2} \left( {{\mathrm{q}}_{0}^{{\upeta {\mathrm{r}}}} + {\mathrm{q}}_{1}^{{\upeta {\mathrm{r}}}} {\mathrm{D}}^{\prime } {\text{ + q}}_{{\mathrm{2}}}^{{\upeta {\mathrm{r}}}} {\mathrm{D}}^{{\prime 2}} } \right)} \\ 0 \\ \end{array} \begin{array}{*{20}c} {{\mathrm{l}}\left( {{\mathrm{q}}_{0}^{{\upxi {\mathrm{c}}}} + {\mathrm{q}}_{1}^{{\upxi {\mathrm{c}}}} {\mathrm{D}}^{\prime } {\text{ + q}}_{{\mathrm{2}}}^{{\upxi {\mathrm{c}}}} {\mathrm{D}}^{{\prime 2}} } \right)} \\ 0 \\ 0 \\ {{\mathrm{l}}^{2} \left( {{\mathrm{q}}_{0}^{{\upxi {\mathrm{r}}}} + {\mathrm{q}}_{1}^{{\upxi {\mathrm{r}}}} {\mathrm{D}}^{\prime } {\text{ + q}}_{{\mathrm{2}}}^{{\upxi {\mathrm{r}}}} {\mathrm{D}}^{{\prime 2}} } \right)} \\ \end{array} } \right]\left\{ {\begin{array}{*{20}c} \upxi \\ \upeta \\ \upalpha \\ \upbeta \\ \end{array} } \right\} \hfill \\ \left\{ {\begin{array}{*{20}c} {{\mathrm{D}}_{1} \left( {{\mathrm{kv}}_{\upxi } + {\text{ me}}\Omega ^{2} {\mathrm{cos}}\left( \uppsi \right) - {\mathrm{mgsin}}\left( {\Omega {\mathrm{t}}} \right)} \right)} \\ {{\mathrm{D}}_{2} \left( {{\text{k v}}_{\upeta } + {\mathrm{me}}\Omega ^{2} {\mathrm{sin}}\left( \uppsi \right) - {\mathrm{mgcos}}\left( {\Omega {\mathrm{t}}} \right)} \right)} \\ {{\mathrm{D}}_{3} \left( {{\mathrm{k}}_{{\mathrm{b}}} \upalpha _{\upxi } + {\text{ k v}}_{\upeta } ({\mathrm{a}} + {\mathrm{a}}1) + \frac{{\mathrm{T}}}{{{\mathrm{l}}_{{\mathrm{h}}} }}{\mathrm{v}}_{\upxi } - {\text{ T }}\upalpha _{\upeta } } \right)} \\ {{\mathrm{D}}_{4} \left( {{\mathrm{k}}_{{\mathrm{b}}} \upalpha _{\upeta } - {\text{ k v}}_{\upxi } ({\mathrm{a}} + {\mathrm{a}}1) + \frac{{\mathrm{T}}}{{{\mathrm{l}}_{{\mathrm{h}}} }}{\mathrm{v}}_{\upeta } + {\text{ T}}\upalpha _{\upxi } } \right)} \\ \end{array} } \right\} \hfill \\ \end{gathered}$$

The forces in the above equations are both static and time-varying; thus, the responses from each are calculated separately and then added to obtain the total response.

#### Time-varying forces

These forces vary with the frequency of the spin speed of the rotor, as derived above, so the solution of the form $\upxi = {\mathrm{Re}} \left[ {{\mathrm{X}}_{1} {\mathrm{e}}^{{{\mathrm{i}}\Omega {\mathrm{t}}}} } \right],$ $\upeta = {\mathrm{Re}} \left[ {{\mathrm{X}}_{2} {\mathrm{e}}^{{{\mathrm{i}}\Omega {\mathrm{t}}}} } \right],$ $\upalpha = {\mathrm{Re}} \left[ {{\mathrm{X}}_{3} {\mathrm{e}}^{{{\mathrm{i}}\Omega {\mathrm{t}}}} } \right]$ and $\upbeta = {\mathrm{Re}} \left[ {{\mathrm{X}}_{4} {\mathrm{e}}^{{{\mathrm{i}}\Omega {\mathrm{t}}}} } \right]$ and using the non-dimensional parameters shown in given in the supplementary material (Appendix 1 (b)), substituting $\mathrm{X}_{3}^{\prime} = \mathrm{lX}_{3}$ and $\mathrm{X}_{4}^{\prime} = \mathrm{lX}_{4}$ and separating the coefficients of ${\mathrm{X}}_{1},{\mathrm{X}}_{2}$ $\mathrm{X}^{\prime}_{3}$ and $\mathrm{X}^{\prime}_{4}$ the equations are written again as

60 $$\left[ {\begin{array}{*{20}c} {{\mathrm{B}}_{1} } & {{\mathrm{B}}_{2} } & 0 & {{\mathrm{B}}_{3} } \\ {{\mathrm{B}}_{4} } & {{\mathrm{B}}_{5} } & {{\mathrm{B}}_{6} } & 0 \\ 0 & {{\mathrm{B}}_{7} } & {{\mathrm{B}}_{8} } & {{\mathrm{B}}_{9} } \\ {{\mathrm{B}}_{{10}} } & 0 & {{\mathrm{B}}_{{11}} } & {{\mathrm{B}}_{{12}} } \\ \end{array} } \right] = \left[ {\begin{array}{*{20}c} {{\mathrm{X}}_{1} } \\ {{\mathrm{X}}_{2} } \\ {{\mathrm{X}}^{\prime } _{3} } \\ {{\mathrm{X}}^{\prime } _{4} } \\ \end{array} } \right]\left\{ {\left[ {\begin{array}{*{20}c} {{\mathrm{F}}_{1} } \\ {{\mathrm{F}}_{2} } \\ {{\mathrm{F}}_{3} } \\ {{\mathrm{F}}_{4} } \\ \end{array} } \right]} \right\}$$

Where, $\left[\mathrm{B}\right]$ matrix is taken from

$$\begin{gathered} {\mathrm{F}}_{1} = \left( {{\mathrm{r}}_{0}^{{\upxi {\mathrm{t}}}} {\mathrm{r}}_{0}^{{\upxi {\mathrm{c}}}} + \left( {{\mathrm{ir}}_{1}^{{\upxi {\mathrm{t}}}} {\mathrm{r}}_{0}^{{\upxi {\mathrm{c}}}} + {\mathrm{ir}}_{0}^{{\upxi {\mathrm{t}}}} {\mathrm{r}}_{1}^{{\upxi {\mathrm{c}}}} } \right)\Omega + \left( { - {\mathrm{r}}_{2}^{{\upxi {\mathrm{t}}}} {\mathrm{r}}_{0}^{{\upxi {\mathrm{c}}}} - {\mathrm{r}}_{1}^{{\upxi {\mathrm{t}}}} {\mathrm{r}}_{1}^{{\upxi {\mathrm{c}}}} - {\mathrm{r}}_{0}^{{\upxi {\mathrm{t}}}} {\mathrm{r}}_{2}^{{\upxi {\mathrm{c}}}} } \right)\Omega ^{2} + \left( { - {\mathrm{ir}}_{2}^{{\upxi {\mathrm{t}}}} {\mathrm{r}}_{1}^{{\upxi {\mathrm{c}}}} - {\mathrm{ir}}_{1}^{{\upxi {\mathrm{t}}}} {\mathrm{r}}_{2}^{{\upxi {\mathrm{c}}}} } \right)\Omega ^{3} } \right. \hfill \\ + \left. {{\mathrm{r}}_{2}^{{\upxi {\mathrm{t}}}} {\mathrm{r}}_{2}^{{\upxi {\mathrm{c}}}} \Omega ^{4} } \right)\left( {{\mathrm{k}}_{0} \left( {{\mathrm{v}}_{{\mathrm{x}}} - {\mathrm{iv}}_{{\mathrm{y}}} } \right) + {\mathrm{i}}\updelta _{{{\mathrm{st}}}} } \right); \hfill \\ \end{gathered}$$

$$\begin{gathered} {\mathrm{F}}_{2} = \left( {{\mathrm{r}}_{0}^{{\upeta {\mathrm{t}}}} {\mathrm{r}}_{0}^{{\upeta {\mathrm{c}}}} + \left( {{\mathrm{ir}}_{1}^{{\upeta {\mathrm{t}}}} {\mathrm{r}}_{0}^{{\upeta {\mathrm{c}}}} + {\mathrm{ir}}_{0}^{{\upeta {\mathrm{t}}}} {\mathrm{r}}_{1}^{{\upeta {\mathrm{c}}}} } \right)\Omega } \right. + \left( { - {\mathrm{r}}_{2}^{{\upeta {\mathrm{t}}}} {\mathrm{r}}_{0}^{{\upeta {\mathrm{c}}}} - {\mathrm{r}}_{1}^{{\upeta {\mathrm{t}}}} {\mathrm{r}}_{1}^{{\upxi {\mathrm{c}}}} - {\mathrm{r}}_{0}^{{\upeta {\mathrm{t}}}} {\mathrm{r}}_{2}^{{\upeta {\mathrm{c}}}} } \right)\Omega ^{2} + \left( { - {\mathrm{ir}}_{2}^{{\upeta {\mathrm{t}}}} {\mathrm{r}}_{1}^{{\upeta {\mathrm{c}}}} - {\mathrm{ir}}_{1}^{{\upeta {\mathrm{t}}}} {\mathrm{r}}_{2}^{{\upeta {\mathrm{c}}}} } \right)\Omega ^{3} \hfill \\ + {\mathrm{r}}_{2}^{{\upeta {\mathrm{t}}}} {\mathrm{r}}_{2}^{{\upeta {\mathrm{c}}}} \Omega ^{4} )({\mathrm{k}}_{0} ({\mathrm{v}}_{{\mathrm{y}}} + {\text{ iv}}_{{\mathrm{x}}} ) + {\text{ i }}\updelta _{{{\mathrm{st}}}} ); \hfill \\ \end{gathered}$$

$$\begin{gathered} {\mathrm{F}}_{3} = \left( {{\mathrm{r}}_{0}^{{\upeta {\mathrm{r}}}} {\mathrm{r}}_{0}^{{\upeta {\mathrm{c}}}} + \left( {{\mathrm{ir}}_{1}^{{\upeta {\mathrm{r}}}} {\mathrm{r}}_{0}^{{\upeta {\mathrm{c}}}} + {\mathrm{ir}}_{0}^{{\upeta {\mathrm{r}}}} {\mathrm{r}}_{1}^{{\upeta {\mathrm{c}}}} } \right)\Omega } \right. + \left( { - {\mathrm{r}}_{2}^{{\upeta {\mathrm{r}}}} {\mathrm{r}}_{0}^{{\upeta {\mathrm{c}}}} - {\mathrm{r}}_{1}^{{\upeta {\mathrm{r}}}} {\mathrm{r}}_{1}^{{\upxi {\mathrm{c}}}} - {\mathrm{r}}_{0}^{{\upeta {\mathrm{r}}}} {\mathrm{r}}_{2}^{{\upeta {\mathrm{c}}}} } \right)\Omega ^{2} + \left( { - {\mathrm{ir}}_{2}^{{\upeta {\mathrm{r}}}} {\mathrm{r}}_{1}^{{\upeta {\mathrm{c}}}} - {\mathrm{ir}}_{1}^{{\upeta {\mathrm{r}}}} {\mathrm{r}}_{2}^{{\upeta {\mathrm{c}}}} } \right)\Omega ^{3} \hfill \\ + {\mathrm{r}}_{2}^{{\upeta {\mathrm{r}}}} {\mathrm{r}}_{2}^{{\upeta {\mathrm{c}}}} \Omega ^{4} )({\mathrm{k}}_{{{\mathrm{b}}_{0} }} (\upalpha _{{\mathrm{x}}} - {\mathrm{i}}\upalpha _{{\mathrm{y}}} ) + {\mathrm{k}}_{0} ({\mathrm{v}}_{{\mathrm{y}}} + {\text{ iv}}_{{\mathrm{x}}} )({\mathrm{a}}_0 + {\mathrm{a}}_{10}) + \frac{{{\mathrm{T}}_{0} }}{{{\mathrm{l}}_{{{\mathrm{h}}0}} }}({\mathrm{v}}_{{\mathrm{x}}} - {\text{ iv}}_{{\mathrm{y}}} ) \hfill \\ \left. { - {\mathrm{T}}_{0} (\upalpha _{{\mathrm{y}}} + {\mathrm{i}}\upalpha _{{\mathrm{x}}} )} \right); \hfill \\ \end{gathered}$$

$$\begin{gathered} {\mathrm{F}}_{4} = \left( {{\mathrm{r}}_{0}^{{\upxi {\mathrm{r}}}} {\mathrm{r}}_{0}^{{\upxi {\mathrm{c}}}} + \left( {{\mathrm{ir}}_{1}^{{\upxi {\mathrm{r}}}} {\mathrm{r}}_{0}^{{\upxi {\mathrm{c}}}} + {\mathrm{ir}}_{0}^{{\upxi {\mathrm{r}}}} {\mathrm{r}}_{1}^{{\upxi {\mathrm{c}}}} } \right)\Omega + \left( { - {\mathrm{r}}_{2}^{{\upxi {\mathrm{r}}}} {\mathrm{r}}_{0}^{{\upxi {\mathrm{c}}}} - {\mathrm{r}}_{1}^{{\upxi {\mathrm{r}}}} {\mathrm{r}}_{1}^{{\upxi {\mathrm{c}}}} - {\mathrm{r}}_{0}^{{\upxi {\mathrm{r}}}} {\mathrm{r}}_{2}^{{\upxi {\mathrm{c}}}} } \right)\Omega ^{2} + \left( { - {\mathrm{ir}}_{2}^{{\upxi {\mathrm{r}}}} {\mathrm{r}}_{1}^{{\upxi {\mathrm{c}}}} - {\mathrm{ir}}_{1}^{{\upxi {\mathrm{r}}}} {\mathrm{r}}_{2}^{{\upxi {\mathrm{c}}}} } \right)\Omega ^{3} } \right. \hfill \\ + {\mathrm{r}}_{2}^{{\upxi {\mathrm{r}}}} {\mathrm{r}}_{2}^{{\upxi {\mathrm{c}}}} \Omega ^{4} )({\mathrm{k}}_{{{\mathrm{b}}_{0} }} (\upalpha _{{\mathrm{y}}} + {\mathrm{i}}\upalpha _{{\mathrm{x}}} ) - {\mathrm{k}}_{0} ({\mathrm{v}}_{{\mathrm{x}}} - {\text{ iv}}_{{\mathrm{y}}} )({\mathrm{a}} + {\mathrm{a}}_1) + \frac{{{\mathrm{T}}_{0} }}{{{\mathrm{l}}_{{{\mathrm{h}}0}} }}({\mathrm{v}}_{{\mathrm{y}}} + {\text{ iv}}_{{\mathrm{x}}} ) \hfill \\ + \left. {{\mathrm{T}}_{0} (\upalpha _{{\mathrm{x}}} - {\mathrm{i}}\upalpha _{{\mathrm{y}}} )} \right); \hfill \\ \end{gathered}$$

The response due to the static force is found by putting all the differential terms to zero, thus

61 $$\begin{gathered} \left[ {\begin{array}{*{20}c} {({\mathrm{q}}_{0}^{{\upxi {\mathrm{t}}}} {\mathrm{r}}_{0}^{{\upxi {\mathrm{c}}}} - {\mathrm{r}}_{0}^{{\upxi {\mathrm{t}}}} {\mathrm{r}}_{0}^{{\upxi {\mathrm{c}}}} \updelta _{{\mathrm{r}}}^{2} )} & 0 & 0 & { - {\mathrm{q}}_{0}^{{\upxi {\mathrm{c}}}} {\mathrm{r}}_{0}^{{\upxi {\mathrm{t}}}} } \\ 0 & {({\mathrm{q}}_{0}^{{\upeta {\mathrm{t}}}} {\mathrm{r}}_{0}^{{\upeta {\mathrm{c}}}} - {\mathrm{r}}_{0}^{{\upeta {\mathrm{t}}}} {\mathrm{r}}_{0}^{{\upeta {\mathrm{c}}}} \updelta _{{\mathrm{r}}}^{2} )} & { - {\mathrm{q}}_{0}^{{\upeta {\mathrm{c}}}} {\mathrm{r}}_{0}^{{\upeta {\mathrm{t}}}} } & 0 \\ 0 & {{\mathrm{q}}_{0}^{{\upeta {\mathrm{c}}}} {\mathrm{r}}_{0}^{{\upeta {\mathrm{r}}}} } & {( - {\mathrm{r}}_{0}^{{\upeta {\mathrm{t}}}} {\mathrm{r}}_{0}^{{\upeta {\mathrm{c}}}} + \frac{1}{{\mathrm{R}}}{\mathrm{G}}0^{2} {\mathrm{r}}_{0}^{{\upeta {\mathrm{c}}}} {\mathrm{r}}_{0}^{{\upeta {\mathrm{r}}}} ( - 1 + {\mathrm{R}})\updelta _{{\mathrm{r}}}^{2} )} & 0 \\ {{\mathrm{q}}_{0}^{{\upxi {\mathrm{c}}}} {\mathrm{r}}_{0}^{{\upxi {\mathrm{r}}}} } & 0 & 0 & {( - {\mathrm{r}}_{0}^{{\upxi {\mathrm{t}}}} {\mathrm{r}}_{0}^{{\upxi {\mathrm{c}}}} + \frac{1}{{\mathrm{R}}}{\mathrm{G}}0^{2} {\mathrm{r}}_{0}^{{\upxi {\mathrm{c}}}} {\mathrm{r}}_{0}^{{\upxi {\mathrm{r}}}} ( - 1 + {\mathrm{R}})\updelta _{{\mathrm{r}}}^{2} )} \\ \end{array} } \right]\left[ {\begin{array}{*{20}c} {{\mathrm{X}}_{{\mathrm{s}_{1} }} } \\ {{\mathrm{X}}_{{\mathrm{s}_{2} }} } \\ {\mathrm{X}^{\prime } _{{\mathrm{s}_{3} }} } \\ {\mathrm{X}^{\prime } _{{\mathrm{s}_{4} }} } \\ \end{array} } \right] \hfill \\ = \left\{ {\begin{array}{*{20}c} {{\mathrm{e}}\updelta _{{\mathrm{r}}}^{2} {\text{ cos}}(\uppsi )} \\ {{\text{ e}}\updelta _{{\mathrm{r}}}^{2} {\text{ sin}}(\uppsi )} \\ 0 \\ 0 \\ \end{array} } \right\} \hfill \\ \end{gathered}$$

X_s1_, ${\mathrm{X}}_{{\mathrm{s}}_{2}}$, ${\mathrm{X}}_{{\mathrm{s}}_{3}}^{\prime}$ and ${\mathrm{X}}_{{\mathrm{s}}_{4}}^{\prime}$ are the static deflections.

### Total forced solution

62 $$\upxi \left(\mathrm{t} \right) = {\mathrm{Re}} \left[ {{\mathrm{X}}_{1} e^{i\Omega \mathrm{t}} } \right] + {\mathrm{X}}_{{\mathrm{s}_{1} }} ,\upeta \left( \mathrm{t} \right) = {\mathrm{Re}} \left[ {{\mathrm{X}}_{2} e^{i\Omega \mathrm{t}} } \right] + {\mathrm{X}}_{{\mathrm{s}_{2} }} ,\upalpha \left( \mathrm{t} \right)l = {\mathrm{Re}} \left[ {{\mathrm{X}}_{3} e^{i\Omega t} } \right] + {\mathrm{X}}_{{\mathrm{s}_{3} }}^{\prime} \,{\mathrm{and}}\,\upbeta \left( t \right) l = {\mathrm{Re}} \left[ {{\mathrm{X}}_{4} e^{i\Omega \mathrm{t}} } \right] + {\mathrm{X}}_{{\mathrm{s}_{4} }}^{\prime}$$

This solution is valid if the above equation is stable, i.e. within the stability limit of the spin speed in this case.

## Result and discussion

### System details

The system considered in the analysis is the same as shown in Fig. 8. Table 3-5 list the values of various system parameters used to study its stability and response. Parameters used for the shaft are presented in the Table 3.

**Table 3:** Parameters for the rotor shaft.

Diameter of the shaft	38 mm
Density of the shaft	7810 kg/m^3^
Flexure Rigidity in ξ direction (EI_ξ_)	21600 Nm^2^
Flexure Rigidity in η direction (EI_η_)	0.8 EI_ξ_
Length of shaft between bearing B and C (a_1_+ a_2_)	1.1 m
Length between coupling and bearing B (a)	0.1 m
Disc location from bearing B (a_1_)	0.3 m
Disk diameter	650 mm
Disk thickness	100 mm
Mass of disc	259.2 kg

Internal Damping of the shaft has not been taken into this work primarily to show the effect of coupling damping. Again, this assumption appears justified according to Kramer^26^, who states that damping in the coupling is more prominent than material damping. Details of the coupling are presented in Table 4. Misalignment and Unbalanced data used in the present analysis are presented in Table 5.

**Table 4:** Details of the coupling.

No. of links	6
Length of link (l_c_)	50 mm
Thickness of link (t)	1 mm
Width of link (w)	26 mm

**Table 5:** Misalignment and unbalance data.

Unbalanced distance (e)	1 mm
Unbalanced angle (ψ)	60 degrees
Parallel misalignment in the x direction	2 mm
Parallel misalignment in y direction	1 mm
Angular misalignment about the x-axis	2 degrees
Angular misalignment about y-axis	3 degrees

A small experiment is reported below to calculate link material properties in given in the supplementary material (Appendix- 2). The material properties are calculated as ${\mathrm{E}}_{\mathrm{c}}$ = 9.855 GPa and ${\upmu }_{\mathrm{c}}$= 15.19 x 106 Pas.

System stiffness coefficients are calculated for the above 6-link coupling and rotor system as

63 $$\begin{gathered} {\mathrm{k}}_{{\upxi {\mathrm{t}}}} = \frac{{3.88 \times 10^{17} + 4.45 \times 10^{14} {\mathrm{D}} + 7.92 \times 10^{6} {\mathrm{D}}^{2} }}{{7.77 \times 10^{10} + 5.92 \times 10^{7} {\mathrm{D}} + {\mathrm{D}}^{2} }}; \hfill \\ {\mathrm{k}}_{{\upxi {\mathrm{c}}}} = \frac{{ - 6.72 \times 10^{16} - 6.62 \times 10^{13} {\mathrm{D}} - 1.16 \times 10^{6} {\mathrm{D}}^{2} }}{{7.77 \times 10^{10} + 5.92 \times 10^{7} {\mathrm{D}} + {\mathrm{D}}^{2} }}; \hfill \\ {\mathrm{k}}_{{\upxi {\mathrm{r}}}} = \frac{{2.5 \times 10^{16} + 2.05 \times 10^{13} {\mathrm{D}} + 3.5 \times 10^{5} {\mathrm{D}}^{2} }}{{7.77 \times 10^{10} + 5.92 \times 10^{7} {\mathrm{D}} + {\mathrm{D}}^{2} }}; \hfill \\ {\mathrm{k}}_{{\upeta {\mathrm{t}}}} = \frac{{1.45 \times 10^{16} + 1.31 \times 10^{13} {\mathrm{D}} + 2.8 \times 10^{5} {\mathrm{D}}^{2} }}{{5.59 \times 10^{10} + 4.74 \times 10^{7} {\mathrm{D}} + {\mathrm{D}}^{2} }}; \hfill \\ {\mathrm{k}}_{{\upeta {\mathrm{c}}}} = \frac{{ - 3.99 \times 10^{16} - 4.23 \times 10^{13} {\mathrm{D}} - 9.27 \times 10^{5} {\mathrm{D}}^{2} }}{{5.59 \times 10^{10} + 4.74 \times 10^{7} {\mathrm{D}} + 1.{\mathrm{D}}^{2} }}; \hfill \\ {\mathrm{k}}_{{\upeta {\mathrm{r}}}} = \frac{{1.45 \times 10^{16} + 1.31 \times 10^{13} {\mathrm{D}} + 2.8 \times 10^{5} {\mathrm{D}}^{2} }}{{5.59 \times 10^{10} + 4.74 \times 10^{7} {\mathrm{D}} + {\mathrm{D}}^{2} }}; \hfill \\ \end{gathered}$$

### Variation of coupling stiffness

The coupling stiffness obtained in Tables 1 and 2 is represented by k (lateral stiffness) and k_b_ (bending stiffness). The values of this stiffness depend on the material, dimension, and number of links (n) of the coupling. Using the non-dimensional parameters shown in given in the supplementary material (Appendix 1 (b)), coupling stiffness can be written as:

64 $${\mathrm{k}}_{0} =3.623\times {10}^{-7}\mathrm{n}(\frac{1.08405\times {10}^{10}\text{ t}0\mathrm{w}0}{{\mathrm{l}}_{{\mathrm{c}}_{0}}}+\frac{1.08405\times {10}^{10}\mathrm{t}0 {\mathrm{w}0}^{3}}{{\mathrm{l}}_{{\mathrm{c}}_{0}}^{3}})$$

65 $${\mathrm{k}}_{{{\mathrm{b}}_{0} }} = \frac{{653.483{\text{n t}}0^{3} {\text{ w}}0{\text{ Cos}}\left[ {\frac{\uppi }{{\mathrm{n}}}} \right]^{2} }}{{{\mathrm{l}}_{{{\mathrm{c}}_{0} }} }}$$

**Fig. 14:**
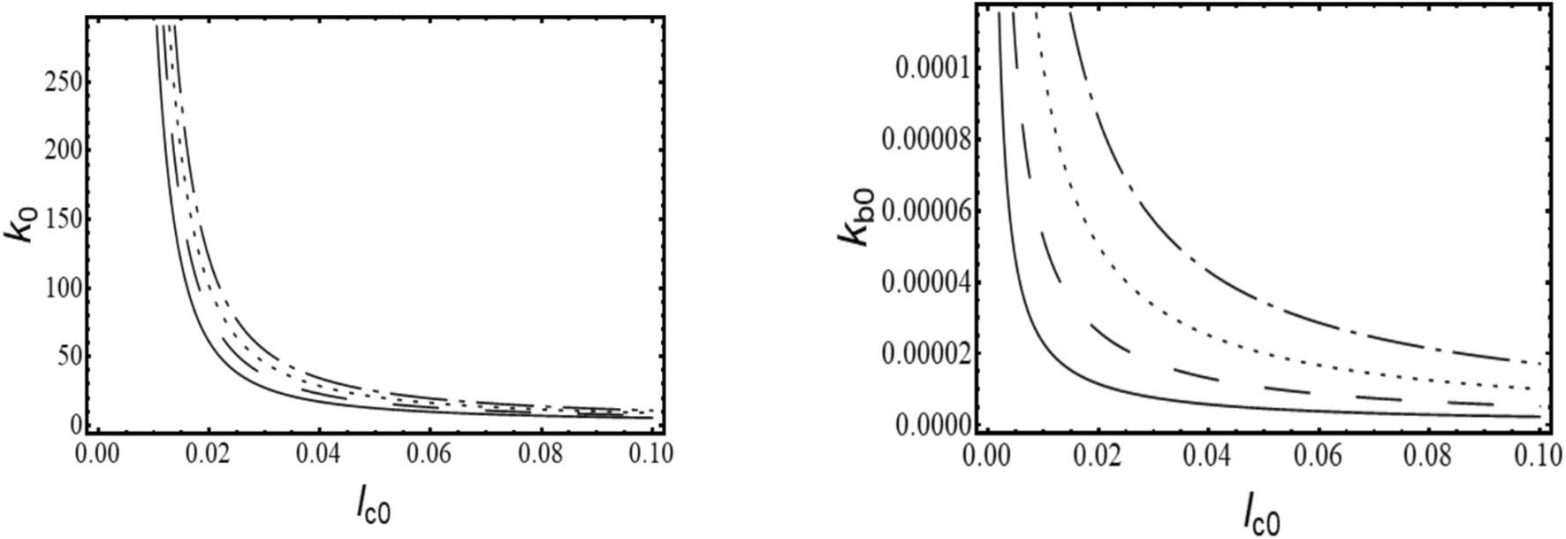
Effect of non-dimensionalised coupling length lc0 on the lateral and bending stiffness constant; w0 = 0.0236, t0 = 0.0009. Key to Fig.: no. of links (n) are represented as n = 6; n = 8; n = 10; n = 12.

**Fig. 15:**
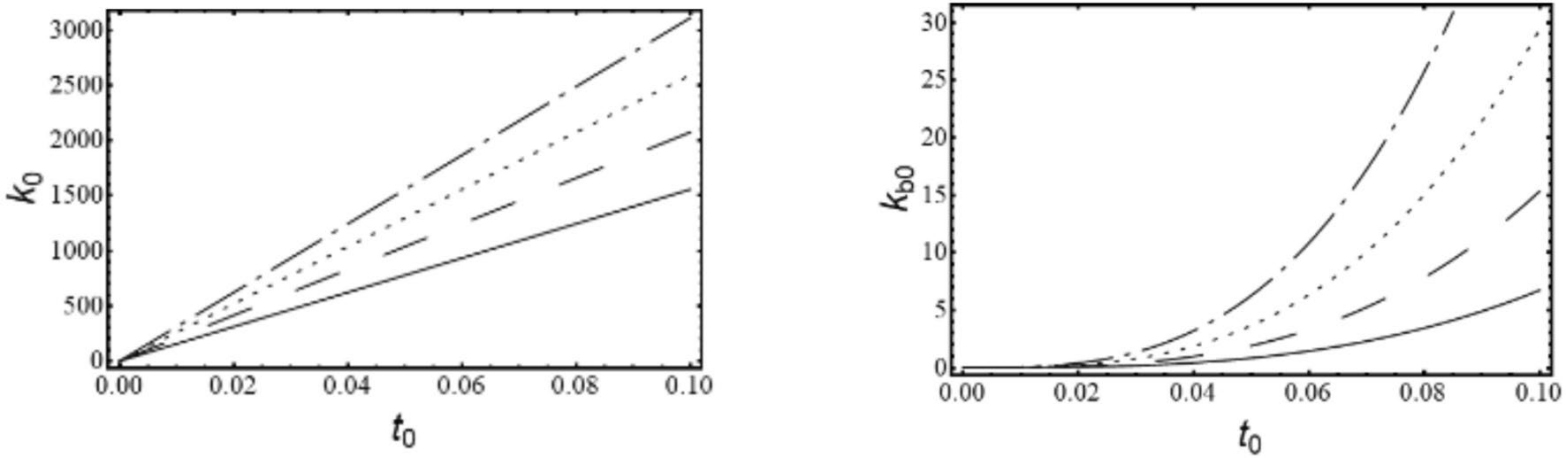
Effect of non-dimensionalised coupling thickness t0 on the lateral and bending stiffness constant; w0 = 0.0236, lc0 = 0.0454.

**Fig. 16:**
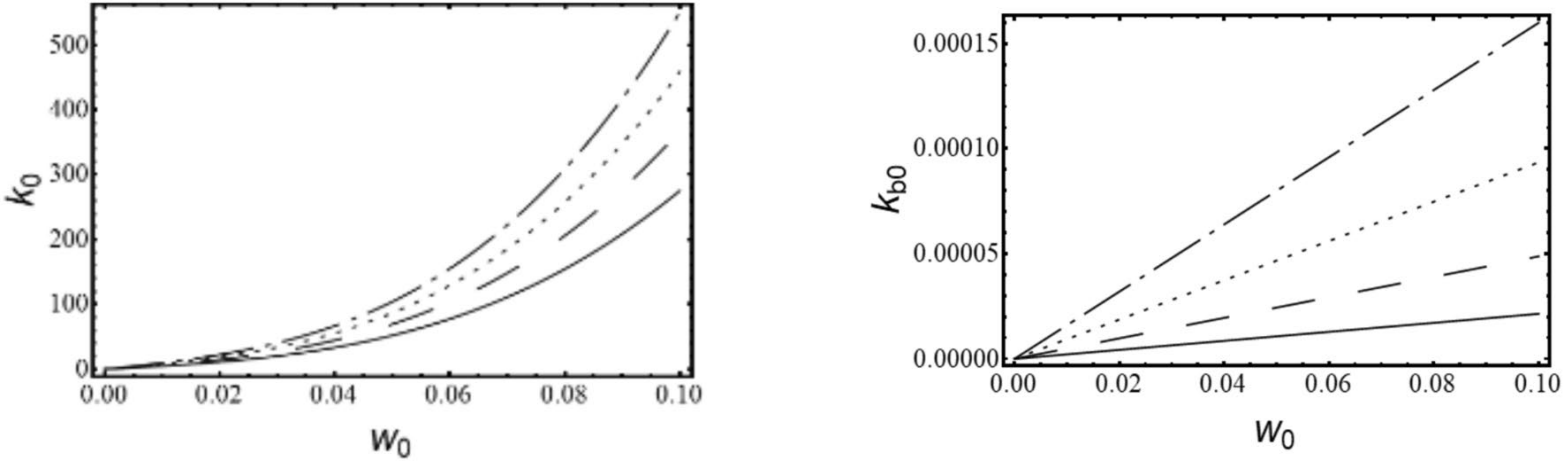
Effect of non-dimensionalized coupling width w0 on the lateral and bending stiffness constant; lc0 = 0.0454, t0 = 0.0009.

Fig.14-16 illustrates the impact of nondimensionalized coupling length ${\mathrm{l}}_{{\mathrm{c}}_{0}}$, width w0, and thickness t0 on the non-dimensionalised lateral stiffness (${\mathrm{k}}_{0}$) and bending stiffness (${\mathrm{k}}_{{\mathrm{b}}_{0}}$) of the coupling. In each diagram, the effect of varying the number of links (n) is also shown.

Examining the deflection function of a beam element, since deflection is inversely proportional to the cube of length and directly proportional to the area moment of Inertia, the lateral stiffness is directly proportional to t0, and the bending stiffness is proportional to t0^3^. This occurs because the neutral axis of the coupling differs for lateral and angular deflection. Similarly, ${\mathrm{k}}_{0}$ and ${\mathrm{k}}_{{\mathrm{b}}_{0}}$ have a different relation with respect to w0 as given in equations 64-65 . In each figure, obviously, as the no. of links increases, the coupling stiffness also increases.

### Stability

The stability limit speed of the system can be calculated by finding the roots of the characteristic equation, which is obtained by equating the determinant of the matrix [B] in equation (60) to zero.

For the solution of the form X e^iwt^, the spin speed (Ω) at which the imaginary part of the roots becomes negative, the system becomes unstable.

**Fig. 17:**
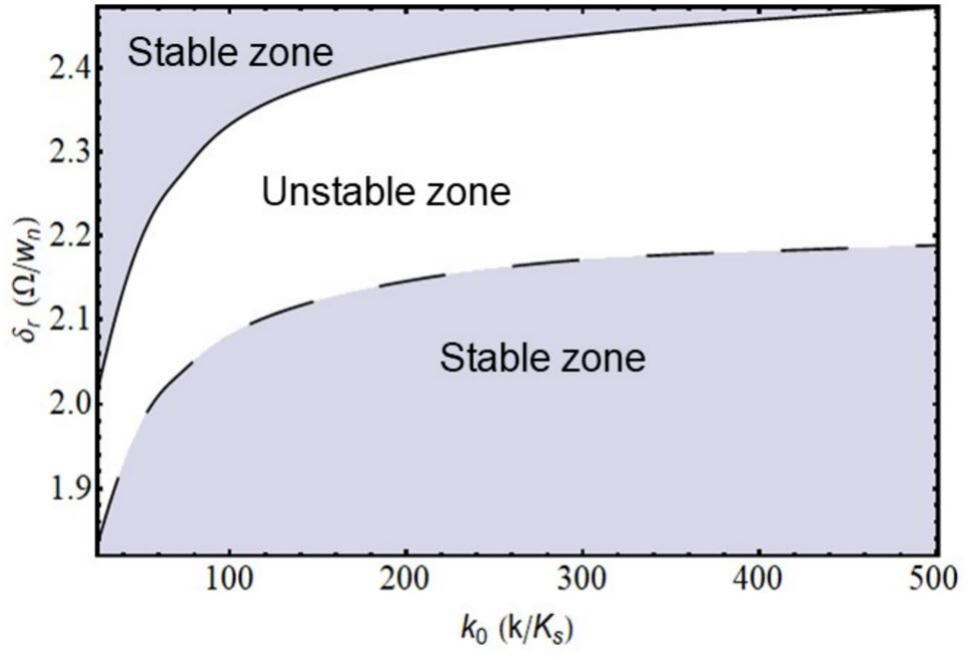
Variation of stability limit speed (δ_r_) with coupling stiffness k.

First, the stability limit of the rotor spin speed is obtained without considering damping and coupling stiffness to form the equivalent stiffness of the system or in other words the rotor is assumed to be continuous one without the presence of the coupling, in this case it may be found that as a result of the asymmetry in the rotor, the system is unstable between non-dimensionalized spin speed, δ_r_ = 1.15 to 1.35 for the example considered. Next, when the coupling stiffness is considered, the system’s equivalent stiffness increases, and it is unstable between δ_r_ = 1.83 and 2.01. Variation of the upper and lower bounds of the stability limit speed with increasing lateral coupling stiffness coefficient k_0_ is shown in Fig. 17; the unshaded region is the unstable region.

When damping is considered in the coupling, stability analysis shows that the system is unstable above all the values of lower speed limit given by dashed line in Fig. 17. With increment of k_0_, the width of the stable as well as unstable zone, both increase initially and almost saturate at k_0_ equal to 500, a situation where coupling may be a rigid one.

The result also concludes that the effect of rotational speed on the flexible coupling. As speed increases substantially on the rotor shaft with a flexible coupling system, the coupling stiffness acts like a rigid one, and, as a result, the cross-coupling stiffness and viscoelastic damping cause severe instability in the system, leading to catastrophic failure.

### Campbell diagram

The Natural Frequency Map of the system can be calculated by finding the roots of the characteristic equation, which is obtained by equating the determinant of the matrix [B] in equation (60) to zero.

**Fig. 18:**
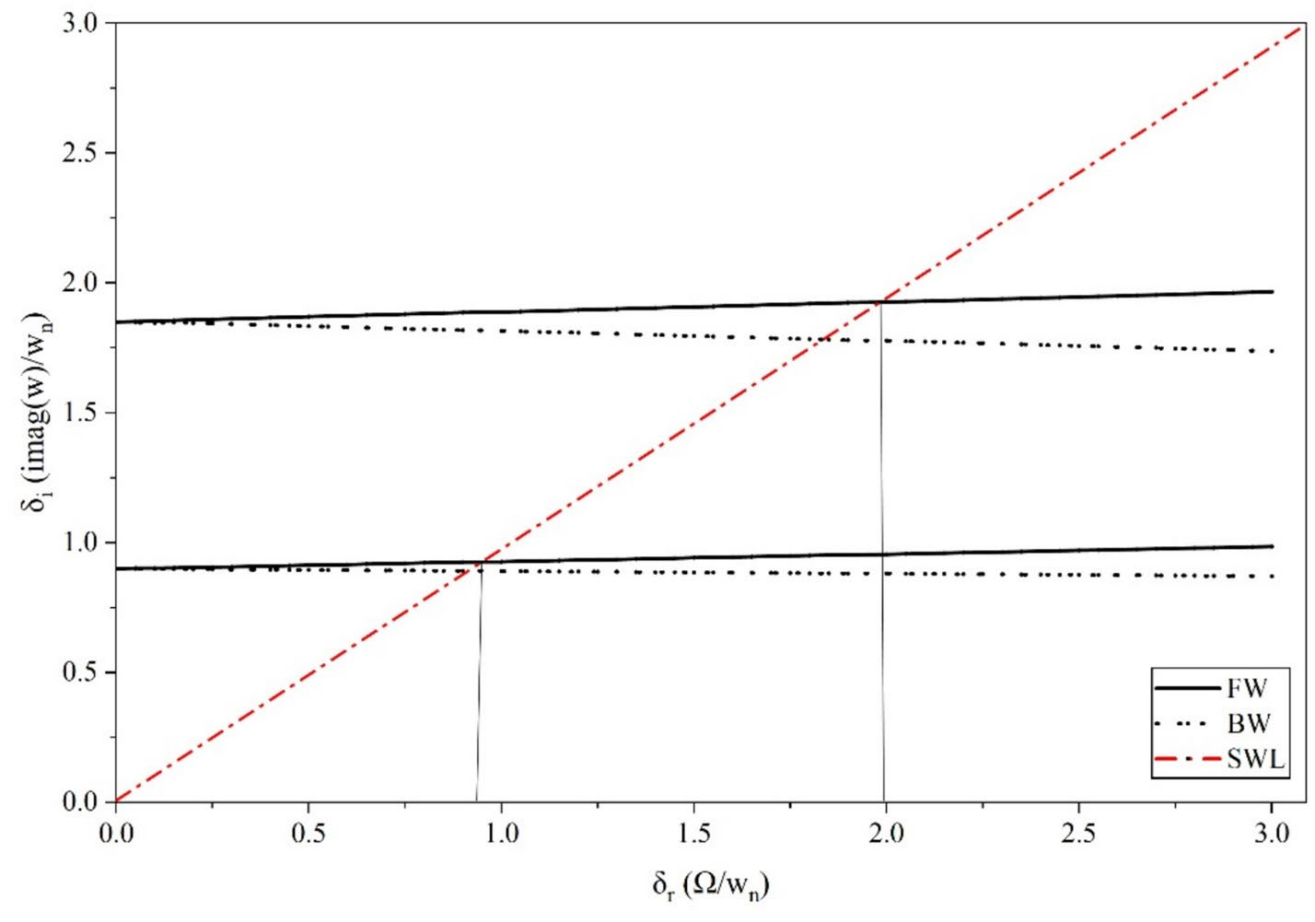
Campbell diagram.

For the solution of the form X e^iwt^, the spin speed (Ω) at which the imaginary part of the roots will be considered for analysis. Fig. 18 shows a nondimensional Campbell diagram for the above-mentioned rotor shaft system with a flexible coupling. The first four frequencies are shown, with the solid line representing the forward whirl (FW) and the dotted line representing the backward whirl (BW). The Synchronous Whirl Line (SWL) intersects the first and second FW lines; the point of intersection represents the system critical speed. The first and second critical speeds are 0.96 and 1.98, respectively.

### Frequency response

The non-dimensionalised amplitude of the steady state response $\text{A }=\frac{\sqrt{{\upxi }^{2}+{\upeta }^{2}}}{\mathrm{e}}$ is calculated for unbalance, gravity and misalignment separately, and then the overall response is added in the time domain.

#### Unbalance

**Fig. 19:**
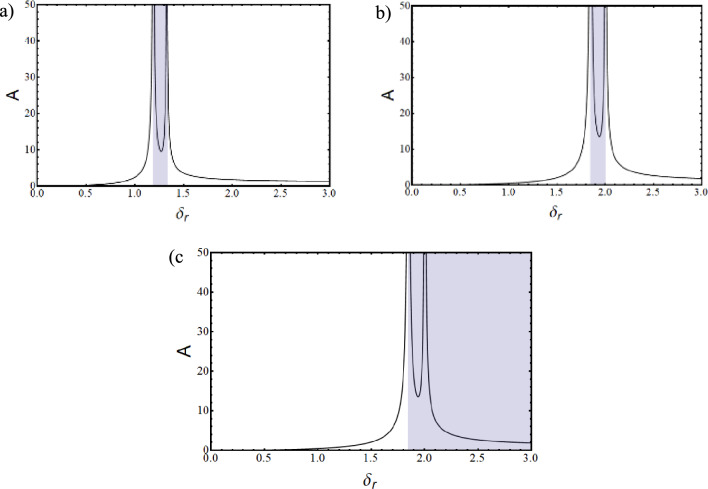
Unbalance frequency response **a**) without coupling stiffness **b**) with coupling stiffness c) with coupling stiffness and damping.

Fig. 19 a) to c) shows the effect of coupling stiffness and damping on the steady state unbalance response (A) of the disc with spin speed ratio (δ_r_). Without misalignment, the system conditions at the coupling end have also changed; thus, the system’s equivalent stiffness increases due to coupling, shifting the peak response to a higher value. Coupling damping does not affect the steady-state response magnitude due to unbalance; however, the system becomes unstable after the first critical point. In the figures below, the shaded portion represents the unstable region. In Fig. 19 a) and b), the instability is due to shaft asymmetry, while in 19 c), it is due to coupling damping.

#### Gravity

**Fig. 20:**
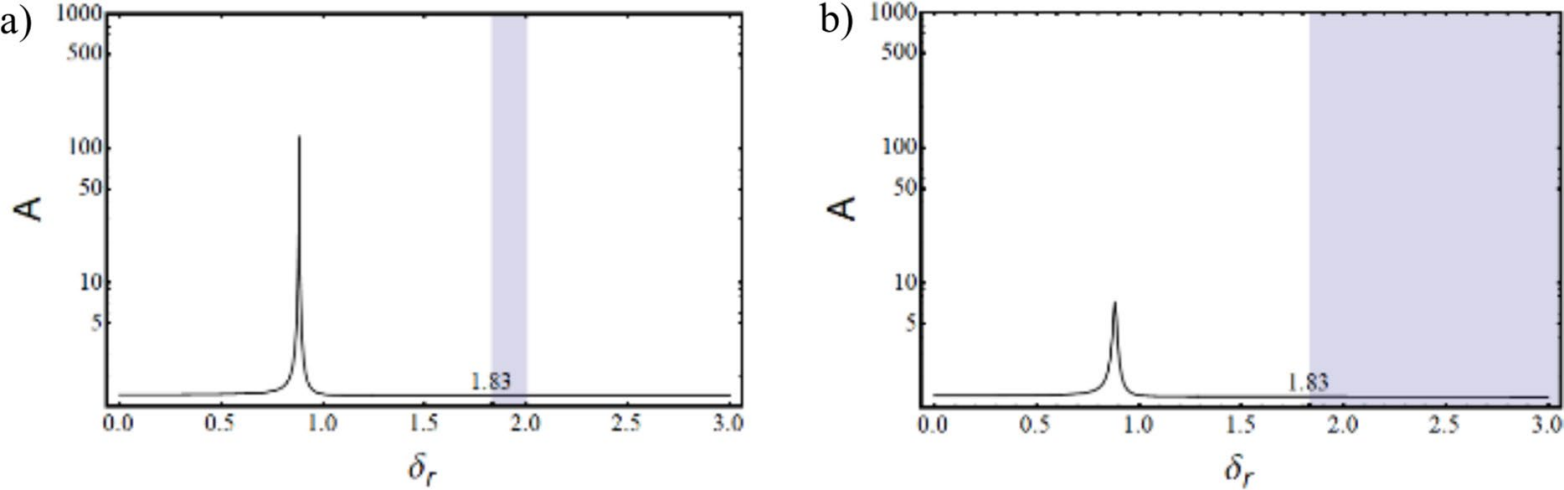
Steady state response due to gravity **a**) without damping **b**) with damping.

Gravity acts as a constant force in the stationary frame. This force leads to a peak response at half the system’s natural frequency when the system is asymmetrical. Damping, when introduced in the coupling, reduces the peak response magnitude at all frequencies, as shown in Fig. 20(b).

#### Misalignment

**Fig. 21:**
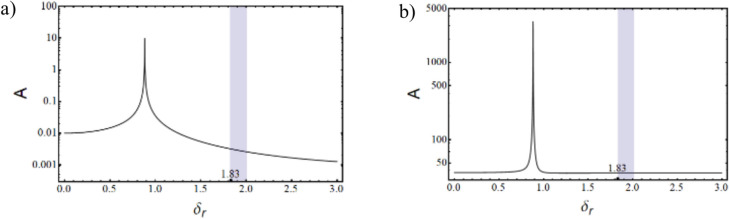
Steady state response without damping due to (**a**) angular misalignment (**b**) parallel misalignment.

**Fig. 22:**
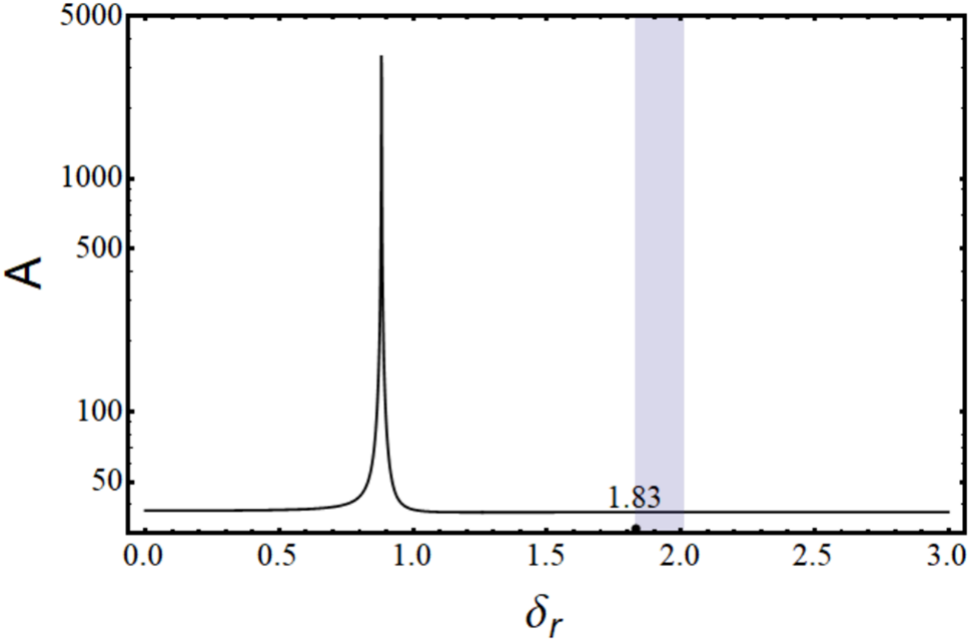
Steady state response due to combined misalignment without damping.

Just like gravity, misalignment also acts as a constant force in the stationary frame. Fig. 21(a) and (b) show the steady state response due to angular and parallel misalignment, respectively. In both cases, peak response occurs at half the natural frequency, but parallel misalignment has a stronger effect on rotor response than angular misalignment. This is to be true in the sense that it is easier to bend a single coupling link than to laterally deflect it, and thus the force produced in the parallel misalignment case is more than the one produced in the angular misalignment. Fig. 22 shows the steady-state response due to combined misalignment; the peak response is similar to that in Fig. 21.

**Fig. 23:**
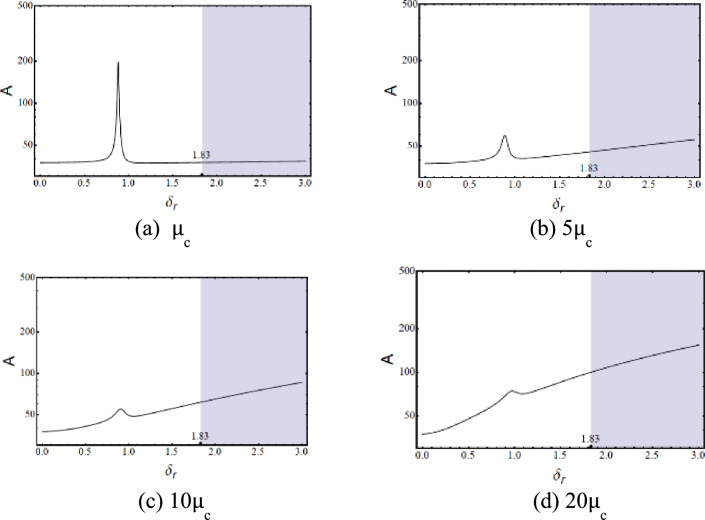
(**a**) to (**d**) shows the effect of increasing the coupling material damping coefficient on the steady state response.

Figs. 23 (a) to (d) show the effect of damping on the steady state response due to misalignment. Damping reduces the peak response for smaller values of the coupling material damping coefficient ${\mu }_{\mathrm{c}}$, but response amplitude increases after a certain value of ${\upmu }_{\mathrm{c}}$. Because damping is present in the coupling stiffness operator, the misalignment-induced force depends on it. Thus, an optimum value of the coupling material damping coefficient ${\upmu }_{\mathrm{c}}$ is desired to have a small-amplitude response over a wide speed range.

#### FFT analysis

**Fig. 24:**
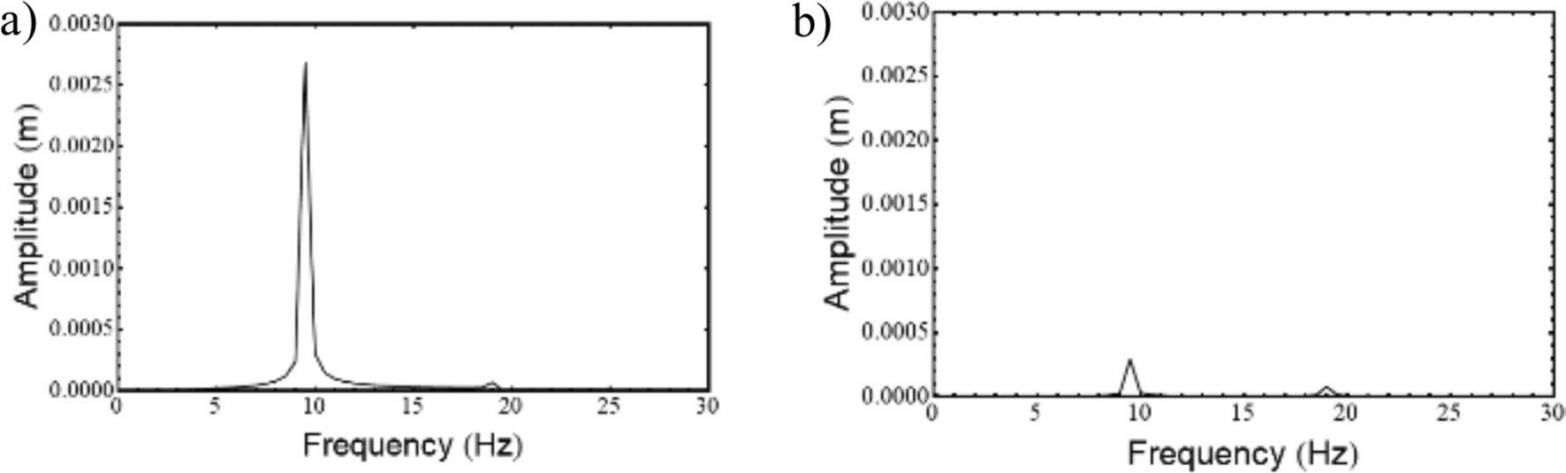
(**a**) FFT without misalignment and neglecting coupling stiffness and (**b**) FFT without misalignment with coupling stiffness.

**Fig. 25:**
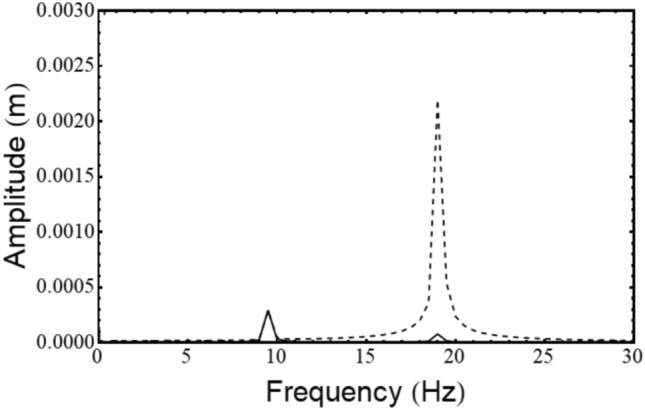
Overlaid FFT with misalignment (dotted) and without misalignment (solid).

The FFT of the time-domain data in the stationary frame is computed for the system running at w = 60 rad/s (9.54 Hz or δ_r_ = 1.095), which is within the stability limit speed. Fig. 24(a) and (b) show that, as the overall system stiffness increases due to the presence of the coupling. The amplitude of the 1X component of the FFT is mainly due to its first bending mode of the system. Once the misalignment is eliminated by using a flexible coupling, the 1X amplitude is drastically reduced in case (b) due to the enhanced coupling stiffness and viscoelastic material used in the links to counter lateral forces. The 2X component always arises due to gravity, even if misalignment is not accounted for in the system. But if both gravity and misalignment are present, as shown in Fig. 25, the 2X amplitude component will affect the system due to parallel and angular misalignment rather than gravity. In such a case, to avoid a 2X-amplitude peak caused by misalignment dominance (dotted line), the use of a flexible segmented disc coupling with a viscoelastic link helps reduce lateral vibration, as shown in Fig. 25 (with solid line).

## Conclusions

- This work provides a theoretical model to determine the characteristics of a seg-mented disc coupling using a viscoelastic model of the segment, and the net cou-pling stiffness and damping are obtained in terms of non-dimensionalised coupling parameters, making it useful irrespective of the coupling’s shape and size.

- The stiffness and damping of the coupling rotate with the spin-synchronized rotor frame and thus modify the equivalent stiffness operator matrix of the system. There-fore, the coupling influences the modal characteristics and response of the rotor shaft system. Most importantly, the damping present in the coupling also acts as an internal damping and may cause instability.

- The use of a viscoelastic-damped material in the links of a flexible segmented disc coupling reduces the peak response near ½ harmonic due to misalignment, but it in-creases the amplitude thereafter; thus, it is necessary to select appropriate damping to achieve a low amplitude across the entire frequency range.

- The dynamic behaviour of the coupled rotor shaft system, in terms of stability and response, is studied after considering the influence of coupling characteristics, misa-lignment (both parallel and angular), unbalance, gravity, and the transmitted torque. After comparing the effects, it is observed that parallel misalignment is more domi-nant than angular misalignment, resulting in higher peak amplitudes in the system at 2X frequency component.

- The FFT of the time-domain data suggests that coupling misalignment magnifies the 2X of the spin frequency component; however, parallel misalignment has a larger in-fluence on its increment than angular misalignment.

- The result also concludes that the effect of rotational speed on the flexible coupling. As speed increases substantially on the rotor shaft with a flexible coupling system, the coupling stiffness effectively becomes rigid, and, as a result, the cross-coupling stiffness and viscoelastic damping cause severe instability in the system, leading to catastrophic failure.

- If the stiffness ratio ${\mathrm{p}}{\kern 1pt} ({\mathrm{EI}}_{\upeta } {\mathrm{/EI}}_{\upxi } )$ is known, it is possible to design a suitable coupling asymmetry to nullify the rotor asymmetry, thereby increasing the stability limit for a non-axisymmetric rotor.

- The coupling stiffness matrix obtained in this work can also be used to model coupling in the finite element analysis of a coupled rotor-shaft system.

## Supplementary Information

Supplementary Information.

## Data Availability

All data generated or analyzed during this study are included in this published article.

## Abbreviation

$\mathrm{x}-\text{ y}-\text{ z}$: Axes of the stationary coordinate frame
$\upxi -\upeta -\upzeta$: Frame rotating with the shaft
$\upalpha ,\upbeta$: Angular deflections clockwise sense about $\upxi$ and $\upeta$
$${\Delta }_{\upxi }$$, $${\Delta }_{\upeta },$$$${\Delta }_{\upzeta }$$: Linear displacements along $$\upxi ,\upeta ,$$ and $$\upzeta$$
$${\Delta }_{\parallel }$$, $${\Delta }_{\perp },$$$${\Delta }_{\mathrm{o}}$$: Parallel, perpendicular and out of the plane misalignment
$\uplambda$: Angle made by the link with $\upxi$ axis
$\updelta$: Exterior angle between two consecutive links
R: Rotation matrix
$\mathrm{c}$: Damping in the coupling link
${\mathrm{l}}_{\mathrm{c}}$: Length of the link
${\mathrm{E}}_{\mathrm{c}}$: Modulus of elasticity of coupling
$\mathrm{A},\text{ w},\text{ t}$: Area of cross section, width and thickness of the link
$\mathrm{I},{\mathrm{I}}_{1},\mathrm{M}$: Area moment of inertia of the link, Area moment of inertia about the neutral axis and moment of inertia
${\mathrm{k}}_{1}, {\mathrm{k}}_{2}$,${\mathrm{k}}_{3}$: Stiffness of the link
${\mathrm{k}}_{\mathrm{b}}, {\mathrm{k}}_{\mathrm{bc}}$: Equivalent bending stiffness, cross-coupling bending stiffness
$\mathrm{k}$: Equivalent lateral stiffness of the coupling
${\mathrm{F}}_{\parallel },{\mathrm{F}}_{\perp }$: Forces parallel and perpendicular
$${\mathrm{F}}_{\upxi }^{\mathrm{t}},$$$${\mathrm{F}}_{\upeta }^{\mathrm{t}}$$: Total force acting on the hub in the $$\upxi$$ and $$\upeta$$ directions
${\mathrm{M}}_{\upxi \upalpha }$: Moments due to angular deflection *α* about $\upxi$ axis
${\mathrm{M}}_{\upeta \upbeta }$: Moments due to angular deflection $\upbeta$ about $\upeta$ axis
$${\mathrm{M}}_{\upxi }^{\mathrm{t}},$$$${\mathrm{M}}_{\upeta }^{\mathrm{t}}$$: Equivalent total moments in the $$\upxi$$ and $$\upeta$$ directions
$\upgamma$: Half interior angle
$\mathrm{E},\upmu$: Young’s modulus, viscosity of the material
${\mathrm{V}}_{\upeta \mathrm{d}}$, ${\upalpha }_{\upxi \mathrm{d}}$: Linear deflection along $\upeta$ direction, angular deflection about $\upxi$ axis at the location of the disc
${\mathrm{V}}_{\upeta \mathrm{c}}$, ${\upalpha }_{\upxi \mathrm{c}}$: Deflection along $\upeta$ direction, angular deflection about $\:{\mathrm{C}}_{\mathrm{i}\mathrm{j}}$ axis at coupling.
$${\mathrm{C}}_{\mathrm{ij}}$$: Influence coefficients
${\mathrm{F}}_{\upeta }$, ${\mathrm{M}}_{\upxi }$: Forces and moments at rotor location,
${\mathrm{F}}_{\upeta 1},{\mathrm{M}}_{\upxi 1}$: Force and moment at the coupling
$${\mathrm{R}}_{1},$$$${\mathrm{R}}_{2}$$: Reaction force
$$\mathrm{un}$$, $$\mathrm{g}$$: Unbalance, gravity
$$\mathrm{T},$$$$\mathrm{ma}$$: Torque transmitted and misalignment
$${\mathrm{I}}_{\mathrm{d}},$$$${\mathrm{I}}_{\mathrm{p}}$$: Diametral and polar mass moments of inertia of the disc
$\mathrm{m}, \Omega$: Mass of the disc and angular velocity of the shaft
${\mathrm{F}}_{\upxi }^{\mathrm{ma}}, {\mathrm{F}}_{\upxi }^{\mathrm{un}}, {\mathrm{F}}_{\upxi }^{\mathrm{g}}$: Forces due to misalignment, unbalance, gravity about $\upxi$ axis
${\mathrm{F}}_{\upeta }^{\mathrm{ma}}, {\mathrm{F}}_{ \upeta }^{\mathrm{un}} ,{\mathrm{F}}_{\upeta }^{\mathrm{g}}$: Forces due to misalignment, unbalance, gravity about $\upeta$ axis
${\mathrm{M}}_{\upxi }^{\mathrm{ma}}, {\mathrm{M}}_{\upxi }^{\mathrm{T}}$: Moments due to torque transmitted and misalignment about $\upxi$ axis
${\mathrm{M}}_{\upeta }^{\mathrm{ma}}, {\mathrm{M}}_{\upeta }^{\mathrm{T}}$: Moments due to torque transmitted and misalignment about $\upeta$ axis
$\mathrm{G},\mathrm{l}$: Radius of gyration, total length of the shaft
$$\mathrm{e}$$: Unbalance distance
$$\uppsi$$: Unbalance angle
${\mathrm{EI}}_{\upxi }$: Flexure Rigidity in $\upxi -$ direction
${\mathrm{EI}}_{\upeta }$: Flexure Rigidity in $\upeta -$ direction
$${\mathrm{k}}_{\mathrm{ij}}$$: Stiffness operator
δ_r_: Non-dimensionalised spin speed,
$${\mathrm{k}}_{\upeta \mathrm{t}}\left(\right),$$$${\mathrm{k}}_{\upeta \mathrm{c}}\left(\right),$$$${\mathrm{k}}_{\upeta \mathrm{r}}\left(\right)$$: Stiffness operators about $$\upeta$$ axis
$${\mathrm{k}}_{\upxi \mathrm{t}}\left(\right),$$$${\mathrm{k}}_{\upxi \mathrm{c}}\left(\right),$$$${\mathrm{k}}_{\upxi \mathrm{r}}\left(\right)$$: Stiffness operators about $$\upxi$$ axis
$${\mathrm{A}}_{\upeta \mathrm{t}}\left(\right),$$$${\mathrm{A}}_{\upeta \mathrm{c}}\left(\right),$$$${\mathrm{A}}_{\upeta \mathrm{r}}\left(\right)$$: Non-dimensionalized Stiffness coefficients about η axis
$${\mathrm{A}}_{\upxi \mathrm{t}}\left(\right),$$$${\mathrm{A}}_{\upxi \mathrm{c}}\left(\right),$$$${\mathrm{A}}_{\upxi \mathrm{r}}\left(\right)$$: Non-dimensionalised Stiffness coefficients about $$\upxi$$ axis
${\mathrm{k}}_{0},{\mathrm{c}}_{0}$: Non-dimensionalised stiffness and damping
$$\mathrm{k}\left(\right),$$$${\mathrm{k}}_{\mathrm{b}}\left(\right)$$: Stiffness operator
$${\mathrm{a}}_{0}, {\mathrm{a}}_{10}, {\mathrm{a}}_{20},\text{ p}$$: Dimensionless parameters
