# Supplementary material for: Dynamic analysis of misalignment modelling with flexible damped coupling in rotor shaft system using classical approach
Source: Sci Rep. 2026 Apr 6;16:16494. doi: 10.1038/s41598-026-40743-9 (PMC13216537; doi:10.1038/s41598-026-40743-9)
Supplement: Supplementary file 1 — Supplementary Information. [file 41598_2026_40743_MOESM1_ESM.docx]

**Supplementary Materials**

# Appendix-1

## Non-Dimensionalization of Stiffness Coefficients

The general stiffness operator in indicial notation is represented as $k_{\mathrm{ij}}$where $i=\eta and \xi$, and $j=t,c and r$. The nature of these coefficients is given below.

$k_{\eta t}$ and $k_{\xi t}$ are $F/d$ or $\mathrm{Force}/{unit deflection}$

$k_{\eta c}$ and $k_{\xi c}$ are $F/\mathrm{ad}$or $\mathrm{Force}/{unit angular deflection}$and also $M/d$or$\mathrm{Moment}/{unit deflection}$ $k_{\eta r}$ and $k_{\xi r}$ are $M/\mathrm{ad}$ or $\mathrm{Moment}/{unit angular deflection}$

| $k_{\eta t} =\frac{\begin{aligned} (3\mathrm{EI}_{\eta}(a_{1}^{4}(3a^{2}k\mathrm{EI}_{\eta}+a^{3}kk_{b}+3\mathrm{EI}_{\eta}k_{b}) \\ +4a_{1}a_{2}^{3}\left( 3a^{2}k\mathrm{EI}_{\eta}+a^{3}kk_{b}+3\mathrm{EI}_{\eta}k_{b} \right) \\ +a_{1}^{3}\left( 12\mathrm{EI}_{\eta}^{2}+a^{4}kk_{b}+4\mathrm{EI}_{\eta}\left( a^{3}k+3ak_{b} \right) \right) \\ +a_{2}^{3}(12\mathrm{EI}_{\eta}^{2}+a^{4}kk_{b}+4\mathrm{EI}_{\eta}(a^{3}k+3ak_{b})))) \end{aligned}}{\begin{aligned} (a_{1}^{3}a_{2}^{3}(12\mathrm{EI}_{\eta}^{2}+a^{3}k(a+a_{1})k_{b} \\ +\mathrm{EI}_{\eta}(4a^{3}k+3a^{2}ka_{1}+12ak_{b}+3a_{1}k_{b}))) \end{aligned}}$ |
| --- |
| $k_{\eta c}=\frac{\begin{aligned} (3\mathrm{EI}_{\eta}(a_{1}^{3}(3a^{2}k\mathrm{EI}_{\eta}+a^{3}kk_{b}+3\mathrm{EI}_{\eta}k_{b}) \\ -2a_{1}a_{2}^{2}\left( 3a^{2}k\mathrm{EI}_{\eta}+a^{3}kk_{b}+3\mathrm{EI}_{\eta}k_{b} \right) \\ +a_{1}^{2}\left( 12\mathrm{EI}_{\eta}^{2}+a^{4}kk_{b}+4\mathrm{EI}_{\eta}\left( a^{3}k+3ak_{b} \right) \right) \\ -a_{2}^{2}(12\mathrm{EI}_{\eta}^{2}+a^{4}kk_{b}+4\mathrm{EI}_{\eta}(a^{3}k+3ak_{b})))) \end{aligned}}{\begin{aligned} (a_{1}^{2}a_{2}^{2}(12\mathrm{EI}_{\eta}^{2}+a^{3}k(a+a_{1})k_{b} \\ +\mathrm{EI}_{\eta}(4a^{3}k+3a^{2}ka_{1}+12ak_{b}+3a_{1}k_{b}))) \end{aligned}}$ |
| $k_{\eta r}=\frac{\begin{aligned} (\mathrm{EI}_{\eta}(3a_{1}^{2}(3a^{2}k\mathrm{EI}_{\eta}+a^{3}kk_{b}+3\mathrm{EI}_{\eta}k_{b}) \\ +3a_{2}\left( 12\mathrm{EI}_{\eta}^{2}+a^{4}kk_{b}+4\mathrm{EI}_{\eta}\left( a^{3}k+3ak_{b} \right) \right) \\ +a_{1}(36\mathrm{EI}_{\eta}^{2}+a^{3}k(3a+4a_{2})k_{b} \\ +12\mathrm{EI}_{\eta}(a^{3}k+a^{2}ka_{2}+3ak_{b}+a_{2}k_{b})))) \end{aligned}}{\begin{aligned} (a_{1}a_{2}(12\mathrm{EI}_{\eta}^{2}+a^{3}k(a+a_{1})k_{b} \\ +\mathrm{EI}_{\eta}(4a^{3}k+3a^{2}ka_{1}+12ak_{b}+3a_{1}k_{b}))) \end{aligned}}$ |
| $k_{\xi t} =\frac{\begin{aligned} (3\mathrm{EI}_{\xi}(a_{1}^{4}(3a^{2}k\mathrm{EI}_{\xi}+a^{3}kk_{b}+3\mathrm{EI}_{\xi}k_{b}) \\ +4a_{1}a_{2}^{3}\left( 3a^{2}k\mathrm{EI}_{\xi}+a^{3}kk_{b}+3\mathrm{EI}_{\xi}k_{b} \right) \\ +a_{1}^{3}\left( 12\mathrm{EI}_{\xi}^{2}+a^{4}kk_{b}+4\mathrm{EI}_{\xi}\left( a^{3}k+3ak_{b} \right) \right) \\ +a_{2}^{3}(12\mathrm{EI}_{\xi}^{2}+a^{4}kk_{b}+4\mathrm{EI}_{\xi}(a^{3}k+3ak_{b})))) \end{aligned}}{\begin{aligned} (a_{1}^{3}a_{2}^{3}(12\mathrm{EI}_{\xi}^{2}+a^{3}k(a+a_{1})k_{b} \\ +\mathrm{EI}_{\xi}(4a^{3}k+3a^{2}ka_{1}+12ak_{b}+3a_{1}k_{b}))) \end{aligned}}$ |
| $k_{\xi c} =\frac{\begin{aligned} (3\mathrm{EI}_{\xi}(a_{1}^{3}(3a^{2}k\mathrm{EI}_{\xi}+a^{3}kk_{b}+3\mathrm{EI}_{\xi}k_{b}) \\ -2a_{1}a_{2}^{2}\left( 3a^{2}k\mathrm{EI}_{\xi}+a^{3}kk_{b}+3\mathrm{EI}_{\xi}k_{b} \right) \\ +a_{1}^{2}\left( 12\mathrm{EI}_{\xi}^{2}+a^{4}kk_{b}+4\mathrm{EI}_{\xi}\left( a^{3}k+3ak_{b} \right) \right) \\ -a_{2}^{2}(12\mathrm{EI}_{\xi}^{2}+a^{4}kk_{b}+4\mathrm{EI}_{\xi}(a^{3}k+3ak_{b})))) \end{aligned}}{\begin{aligned} (a_{1}^{2}a_{2}^{2}(12\mathrm{EI}_{\xi}^{2}+a^{3}k(a+a_{1})k_{b} \\ +\mathrm{EI}_{\xi}(4a^{3}k+3a^{2}ka_{1}+12ak_{b}+3a_{1}k_{b}))) \end{aligned}}$ |
| $k_{\xi r} =\frac{\begin{aligned} (\mathrm{EI}_{\xi}(3a_{1}^{2}(3a^{2}k\mathrm{EI}_{\xi}+a^{3}kk_{b}+3\mathrm{EI}_{\xi}k_{b}) \\ +3a_{2}\left( 12\mathrm{EI}_{\xi}^{2}+a^{4}kk_{b}+4\mathrm{EI}_{\xi}\left( a^{3}k+3ak_{b} \right) \right) \\ +a_{1}(36\mathrm{EI}_{\xi}^{2}+a^{3}k(3a+4a_{2})k_{b} \\ +12\mathrm{EI}_{\xi}(a^{3}k+a^{2}ka_{2}+3ak_{b}+a_{2}k_{b})))) \end{aligned}}{\begin{aligned} (a_{1}a_{2}(12\mathrm{EI}_{\xi}^{2}+a^{3}k(a+a_{1})k_{b} \\ +\mathrm{EI}_{\xi}(4a^{3}k+3a^{2}ka_{1}+12ak_{b}+3a_{1}k_{b}))) \end{aligned}}$ |

Here $\mathrm{EI}_{\xi}$ and $\mathrm{EI}_{\eta}$ are flexural rigidity of shaft along $\xi and \eta$ axis respectively.

Using $K_{S}=\frac{48\mathrm{EI}_{\xi}}{l^{3}}$ thus $\mathrm{EI}_{\xi}=\frac{K_{S}l^{3}}{48}$. Putting dimensionless parameters as $a_{0}=\frac{a}{l}$, $a_{10}=\frac{a_{1}}{l}$, $a_{20}=\frac{a_{2}}{l}$ $p=\frac{\mathrm{EI}_{\eta}}{\mathrm{EI}_{\xi}}$, $k_{0}=\frac{k}{K_{S}}$ and$k_{b0}=\frac{k_{b}}{K_{S}}$.

using the non-dimensional parameters $R=\frac{I_{p}}{I_{d}}$, $I_{p}=mG^{2}$, $G_{o}=\frac{G}{l}$, $\delta_{r}=\frac{\Omega}{w_{n}}$,$\delta_{\mathrm{st}}=\frac{\mathrm{mg}}{w_{n}}$, $l_{ho}={l_{h}}/l$, $T_{o}=T\left( \mathrm{mgl} \right)$, where $w_{n}=\sqrt{\frac{K_{s}}{m}}$, $'G'$ is the radius of gyration and ‘l’ is the total length of shaft

Stiffness coefficients are non-dimensionalised as follow:

$k_{\eta t}=K_{S}A_{\eta t}$

| $A_{\eta t}=\frac{\begin{aligned} (12a_{10}^{4}(a_{0}^{2}k_{0}+k_{b0}+16a_{0}^{3}k_{0}k_{b0}) \\ +48a_{10}a_{20}^{3}\left( a_{0}^{2}k_{0}+k_{b0}+16a_{0}^{3}k_{0}k_{b0} \right) \\ +a_{10}^{3}\left( 1+16a_{0}^{3}k_{0}+48a_{0}k_{b0}+192a_{0}^{4}k_{0}k_{b0} \right) \\ +a_{20}^{3}(1+16a_{0}^{3}k_{0}+48a_{0}k_{b0}+192a_{0}^{4}k_{0}k_{b0})) \end{aligned}}{\begin{aligned} (16a_{10}^{3}a_{20}^{3}(1+12a_{0}^{2}a_{10}k_{0}+48a_{0}k_{b0} \\ +12a_{10}k_{b0}+192a_{0}^{4}k_{0}k_{b0} \\ +16a_{0}^{3}(k_{0}+12a_{10}k_{0}k_{b0}))) \end{aligned}}$  Similarly, $k_{\eta c}=K_{S}lA_{\eta c}$, $k_{\eta r}=K_{S}l^{2}A_{\eta c}$, $k_{\xi t}=K_{S}A_{\xi t}$, $k_{\xi c}=K_{S}lA_{\xi t}$ & $k_{\xi r}=K_{S}l^{2}A_{\xi r}$ |
| --- |
| $A_{\eta c}=\frac{\begin{aligned} (12a_{10}^{3}(a_{0}^{2}k_{0}+k_{b0}+16a_{0}^{3}k_{0}k_{b0}) \\ -24a_{10}a_{20}^{2}\left( a_{0}^{2}k_{0}+k_{b0}+16a_{0}^{3}k_{0}k_{b0} \right) \\ +a_{10}^{2}\left( 1+16a_{0}^{3}k_{0}+48a_{0}k_{b0}+192a_{0}^{4}k_{0}k_{b0} \right) \\ -a_{20}^{2}(1+16a_{0}^{3}k_{0}+48a_{0}k_{b0}+192a_{0}^{4}k_{0}k_{b0})) \end{aligned}}{\begin{aligned} (16a_{10}^{2}a_{20}^{2}(1+12a_{0}^{2}a_{10}k_{0}+48a_{0}k_{b0} \\ +12a_{10}k_{b0}+192a_{0}^{4}k_{0}k_{b0} \\ +16a_{0}^{3}(k_{0}+12a_{10}k_{0}k_{b0}))) \end{aligned}}$ |
| $A_{\eta r}=\frac{\begin{aligned} (12a_{10}^{2}(a_{0}^{2}k_{0}+k_{b0}+16a_{0}^{3}k_{0}k_{b0}) \\ +a_{20}\left( 1+16a_{0}^{3}k_{0}+48a_{0}k_{b0}+192a_{0}^{4}k_{0}k_{b0} \right) \\ +a_{10}(1+16a_{0}^{2}a_{20}k_{0}+48a_{0}k_{b0}+16a_{20}k_{b0} \\ +192a_{0}^{4}k_{0}k_{b0}+16a_{0}^{3}(k_{0}+16a_{20}k_{0}k_{b0}))) \end{aligned}}{\begin{aligned} (16a_{10}a_{20}(1+12a_{0}^{2}a_{10}k_{0}+48a_{0}k_{b0} \\ +12a_{10}k_{b0}+192a_{0}^{4}k_{0}k_{b0} \\ +16a_{0}^{3}(k_{0}+12a_{10}k_{0}k_{b0}))) \end{aligned}}$ |
| $A_{\xi t}=\frac{\begin{aligned} (p(12pa_{0}^{2}a_{10}(a_{10}^{3}+4a_{20}^{3})k_{0} \\ +48pa_{0}\left( a_{10}^{3}+a_{20}^{3} \right)k_{b0}+192a_{0}^{4}\left( a_{10}^{3}+a_{20}^{3} \right)k_{0}k_{b0} \\ +p\left( pa_{10}^{3}+pa_{20}^{3}+12a_{10}^{4}k_{b0}+48a_{10}a_{20}^{3}k_{b0} \right) \\ +16a_{0}^{3}k_{0}(pa_{10}^{3}+pa_{20}^{3}+12a_{10}^{4}k_{b0}+48a_{10}a_{20}^{3}k_{b0}))) \end{aligned}}{\begin{aligned} (16a_{10}^{3}a_{20}^{3}(12pa_{0}^{2}a_{10}k_{0}+48pa_{0}k_{b0} \\ +192a_{0}^{4}k_{0}k_{b0}+p\left( p+12a_{10}k_{b0} \right) \\ +16a_{0}^{3}k_{0}(p+12a_{10}k_{b0}))) \end{aligned}}$ |
| $A_{\xi c}=\frac{\begin{aligned} (p(12pa_{0}^{2}a_{10}(a_{10}^{2}-2a_{20}^{2})k_{0} \\ +48pa_{0}\left( a_{10}^{2}-a_{20}^{2} \right)k_{b0}+192a_{0}^{4}\left( a_{10}^{2}-a_{20}^{2} \right)k_{0}k_{b0} \\ +p\left( pa_{10}^{2}-pa_{20}^{2}+12a_{10}^{3}k_{b0}-24a_{10}a_{20}^{2}k_{b0} \right) \\ +16a_{0}^{3}k_{0}(pa_{10}^{2}-pa_{20}^{2}+12a_{10}^{3}k_{b0}-24a_{10}a_{20}^{2}k_{b0}))) \end{aligned}}{\begin{aligned} (16a_{10}^{2}a_{20}^{2}(12pa_{0}^{2}a_{10}k_{0}+48pa_{0}k_{b0} \\ +192a_{0}^{4}k_{0}k_{b0}+p\left( p+12a_{10}k_{b0} \right) \\ +16a_{0}^{3}k_{0}(p+12a_{10}k_{b0}))) \end{aligned}}$ |
| $A_{\xi r}=\frac{\begin{aligned} (p(4pa_{0}^{2}a_{10}(3a_{10}+4a_{20})k_{0} \\ +48pa_{0}\left( a_{10}+a_{20} \right)k_{b0}+192a_{0}^{4}\left( a_{10}+a_{20} \right)k_{0}k_{b0} \\ +p\left( pa_{20}+12a_{10}^{2}k_{b0}+a_{10}\left( p+16a_{20}k_{b0} \right) \right) \\ +16a_{0}^{3}k_{0}(pa_{20}+12a_{10}^{2}k_{b0}+a_{10}(p+16a_{20}k_{b0})))) \end{aligned}}{\begin{aligned} (16a_{10}a_{20}(12pa_{0}^{2}a_{10}k_{0}+48pa_{0}k_{b0} \\ +192a_{0}^{4}k_{0}k_{b0}+p\left( p+12a_{10}k_{b0} \right) \\ +16a_{0}^{3}k_{0}(p+12a_{10}k_{b0}))) \end{aligned}}$ |

## Non-Dimensionalization of Viscoelastic Parameter

Example: 6 link coupling (from Tables 1 and 2)

$$k=3\left( K_{1}+K_{2} \right)=3\left( \frac{A}{l}+\frac{12l}{l_{C}^{3}} \right)E_{C}$$

For linear viscoelastic material $E_{C}$ is replaced by operator $E_{C}\left( \right)$ and for 2 parameter Kelvin-Voigt model $E_{C}\left( \right)=E_{C}+\mu_{C}D$, where $D$ is $\frac{d}{dt}$. Thus

$$k\left( \right)=3\left( \frac{A}{l}+\frac{12l}{l_{C}^{3}} \right)\left( E_{C}+\mu_{C}D \right)$$

$$k\left( \right)=3\left( \frac{A}{l}+\frac{12l}{l_{C}^{3}} \right)E_{C}+3\left( \frac{A}{l}+\frac{12l}{l_{C}^{3}} \right)\mu_{C}D=k+cD$$

Here, $k$ represents the stiffness of coupling link, is equal to $3\left( \frac{A}{l}+\frac{\mathbf{12l}}{\mathbf{l}_{\mathbf{C}}^{\mathbf{3}}} \right)E_{C}$ and $c$ represents damping in the coupling link is equal to $3\left( \frac{A}{l}+\frac{12l}{l_{C}^{3}} \right)\mu_{C}$

For a disc of mass m non-dimensionalizing stiffness by $K_{S}$ and damping by $C_{S}=2\sqrt{mK_{S}}$ and using $k_{0}=\frac{k}{K_{S}}$ and $c_{0}=\frac{C}{C_{S}}$

$$k\left( \right)=k_{0}K_{S}+c_{0}2\sqrt{mK_{S}} D$$

$k\left( \right)=k_{0}K_{S}\left( 1+\frac{2c_{0}D}{k_{0}\omega_{n}} \right)$ here $\omega_{n}=\sqrt{\frac{K_{S}}{m}}$

$k_{0}\left( \right)=\frac{k\left( \right)}{K_{S}}=k_{0}\left( 1+\Lambda D^{'} \right)$, here $\Lambda=2\frac{c_{0}}{k_{0}}$ and $D^{'}=\frac{D}{\omega_{n}}$

Similarly, following the procedure for calculating $k_{b}\left( \right)$ and replacing $E_{C}\left( \right)=E_{C}+\mu_{C}D$ in $k_{b}$ or a link coupling $k_{b}\left( \right)=1.5 l_{r}^{2}12E_{C}\left( \right)\frac{l_{1}}{l_{3}}=18l_{r}^{2}E_{C}\left( \right)\frac{l_{1}}{l_{3}}$

$k_{b}\left( \right)=k_{b}+k_{b}D$, where $k_{b}=18l_{r}^{2}E_{C}\left( \right)\frac{l_{1}}{l_{3}}$ and $18l_{r}^{2}\mu_{C}D\frac{l_{1}}{l_{3}}$

Using non-dimensionalised parameter $k_{b0}=\frac{k_{b}}{\mathrm{lK}_{S}}$ and $c_{b0}\left( \right)=\frac{c_{b}}{lC_{S}}$

$k_{b0}\left( \right)=\frac{k_{b}\left( \right)}{K_{S}}=k_{b0}\left( 1+\Lambda D^{'} \right)$,, here $\Lambda=2\frac{c_{b0}}{k_{b0}}$ and $D^{'}=\frac{D}{\omega_{n}}$

To incorporate viscoelasticity in the stiffness coefficients obtained in Table 1, replace $k_{0}$ and $k_{b0}$ by $k_{0}\left( \right)$ and $k_{b0}\left( \right)$, thus both the numerator and denominator in $A_{\mathrm{ij}}\left( \right)$ become polynomials in $D^{'}$

Thus, the generalised stiffness operator $k_{\mathrm{ij}}$becomes $k_{\mathrm{ij}}\left( \right)$ and the corresponding coefficients $A_{\mathrm{ij}}$Aij becomes $A_{\mathrm{ij}}\left( \right)$ where $i=\eta and \xi$, and $j=t,c and r$.

# Appendix-2

## Viscoelastic Model Extraction

The links have been assumed, for realistic purposes, to be made of a viscoelastic material that stores and dissipates energy simultaneously during dynamic deformation. This energy depends on the excitation frequency. Thus, various multi-element spring-damper models, such as two-, three-, and four-element models, are used in the literature [9-10] to model the storage and loss behaviour of the material, as shown in Fig. 26.

In this paper, an operator-based approach is used to derive the constitutive relation proposed by Dutt and Roy [13, 14], in which stiffness is represented by a time-differential operator to account for dissipation in the constitutive equation.

| 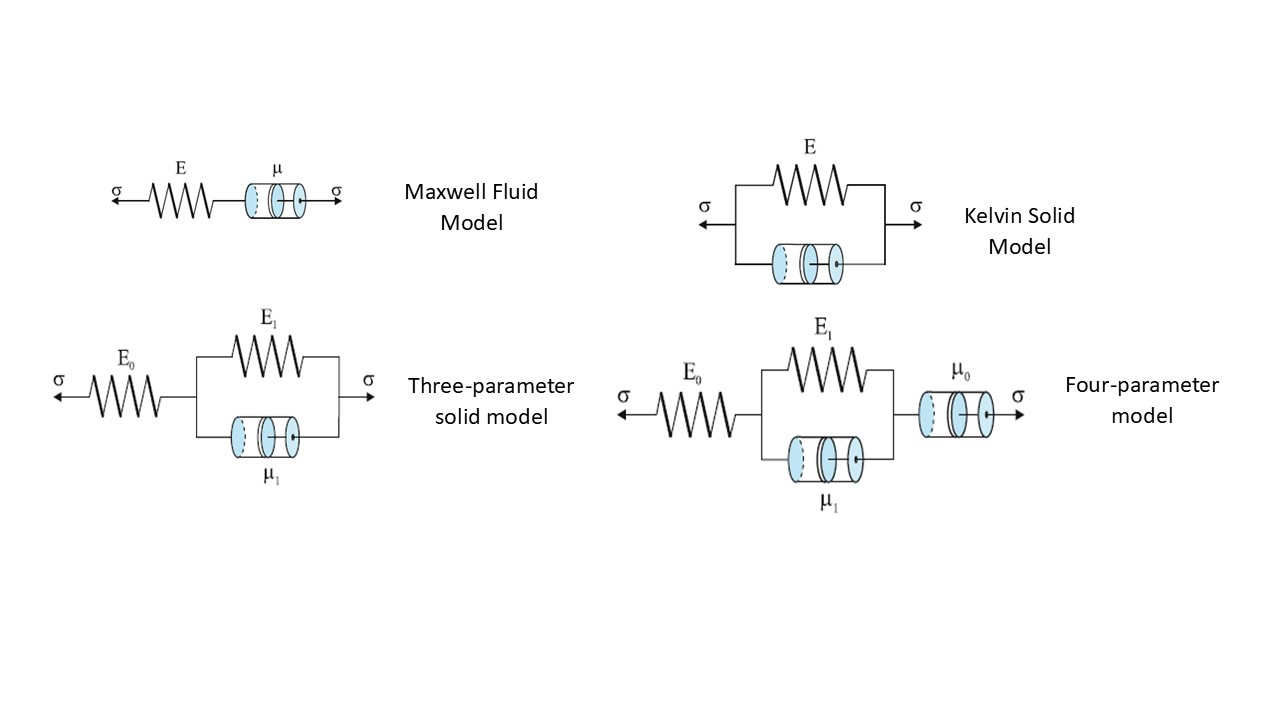 |
| --- |
| **Fig. 26: Spring - Damper arrangement for Maxwell, Kelvin, Three and Four parameter models. Image courtesy Brinson and Brinson [11]** |

In the given work, a 2-parameter Kelvin-Voigt model is assumed for the coupling and the shaft. This is done for simplicity; however, the method can accommodate any constitutive behaviour, represented by the operator. For this, the material constant E is replaced by the operator $E\left( \right)$, which, in this case, is equal to $E+\mu D$ for coupling, where $D=\frac{d}{\mathrm{dt}}$ represents the differential operator, E is Young’s modulus, and $\mu$ is the viscosity of the material. Appendix 1(b) shows an example of the calculation of viscoelastic coupling stiffness.

**Extraction of Material Properties of a link of the coupling**

In the analysis, a 2-parameter Kelvin-Voigt model is assumed for link material modelling, which is given as E( ) = E+$\mu$D. The material used for the link is g11 FRP, and UTM is used to calculate E by following the tensile test standard ASTM - D638-14 (Specimen type IV, for plastics with thickness less than 4 mm, as shown in Fig. 27). The tensile test result shows the load vs. deformation graph in Fig. 19. E is calculated to be equal to 9855 MPa.

|  |
| --- |
| **Fig. 27: Sample Specimen – Tensile test standard for plastic ASTM - D638-14 (Type IV), W = 6, WO = 19, Wc = W to W–0.1 mm, L = 33, LO = 115, G = 25, D = 65, R = 15, and RO = 25** |
| 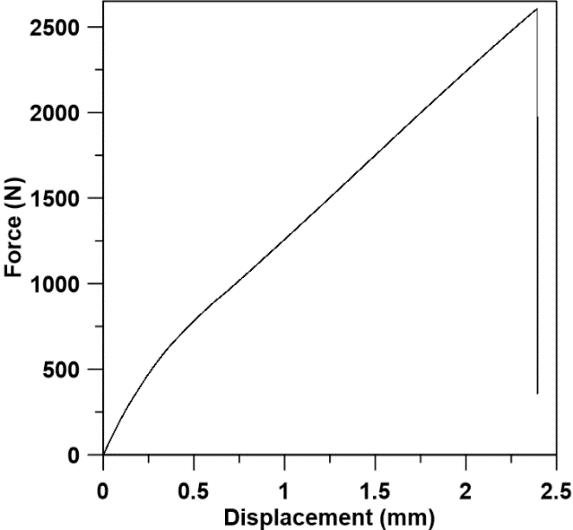 |
| **Fig. 28: Force v/s Displacement graph – UTM** |

Damping in the material is calculated by the cantilever test, and the logarithmic decrement is used to calculate the value of $\mu_{c}$ = 15.19 x 106 (from the experiment setup shown in Fig. 20). The sensitivity of the accelerometer used is 1V/mm.

| 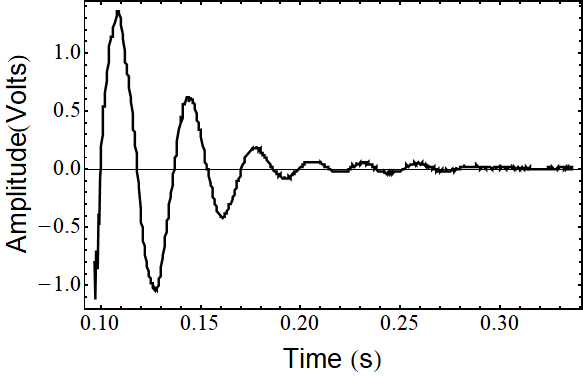 | 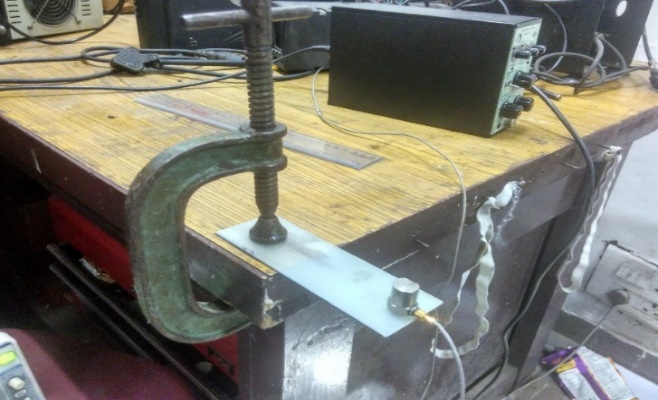 |
| --- | --- |
| **Fig. 29: Cantilever Test Set Up and Result** | |

Viscoelasticity is a property wherein a material exhibits the characteristics of both fluid and solid, and this combined material behaviour is represented by a combination of springs and dampers, and thus, the corresponding constitutive relation is obtained. Two basic models, the Maxwell fluid and Kelvin solid, are used to illustrate relaxation and creep, respectively; however, they have limited applicability in representing the actual stress-strain behaviour of materials. Thus, different multi-element spring-damper models, such as two-, three-, and four-element models, are used to model the material's storage and loss behaviour. While several combinations are possible, a more systematic approach can be followed by connecting Maxwell elements in parallel and thus, obtaining the generalised Wiechert model as shown in Fig. 30, in which, addition of each Maxwell element in parallel introduces a new relaxation field, and thus, allows a more realistic representation of the material behaviour.

| 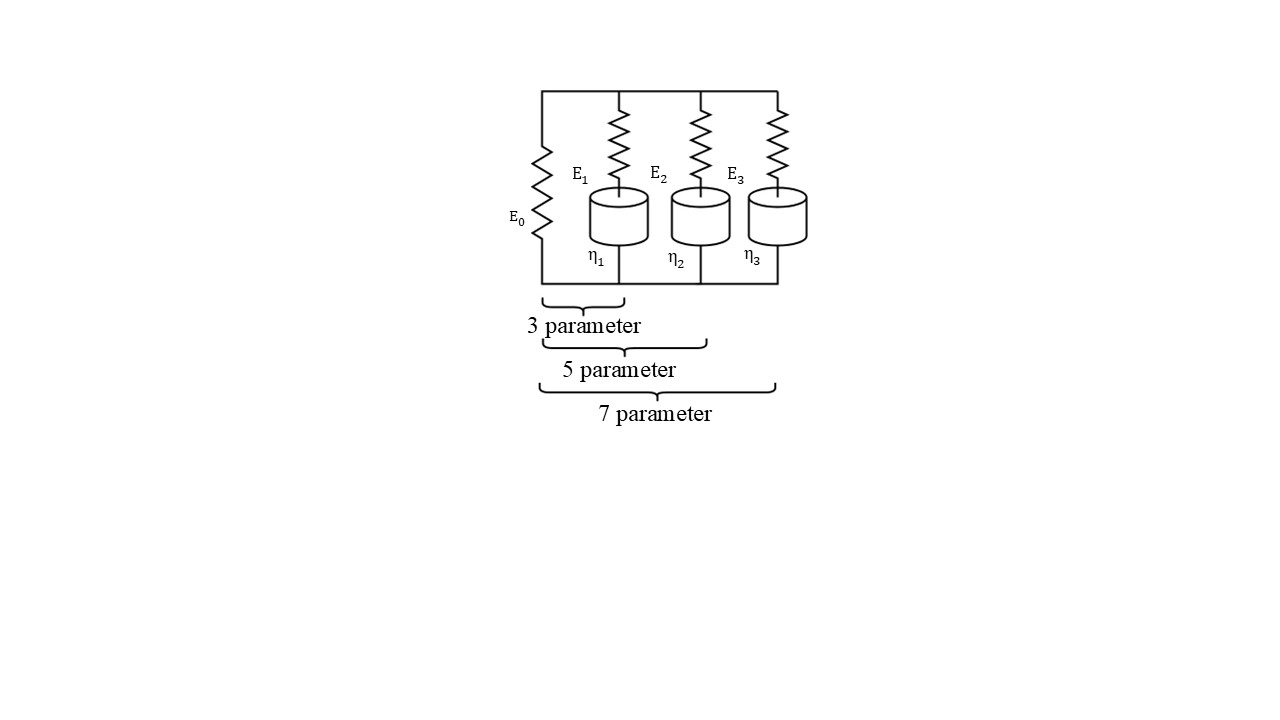 |
| --- |
| **Fig. 30 Wiechert Model** |

The storage and dissipation energy due to dynamic deformation of such material is frequency-dependent and is obtained using an operator-based approach, as proposed by Dutt and Roy [18], in which stiffness is represented as a differential operator of time.

| $E*(D) = E_{0}+\sum_{j=1}^{j=n} \frac{E_{j} D}{\left( \frac{E_{j}}{\eta_{j}} + D \right)}$ | (66) |
| --- | --- |

Following Brinson [17], the frequency-dependent complex modulus (E*) for the Weichert model is obtained as

| E*(w)=E_s_(w)+iE_l_(w) | (67) |
| --- | --- |
| E_s_(storage modulus) =$E_{\infty}+\sum_{j=1}^{j=n} \frac{E_{j} w^{2}}{\left( \frac{E_{j}}{\eta_{j}} \right)^{2}+w^{2}}$ | (68) |
| E_l_ (loss modulus) = $\sum_{j=1}^{j=n} \frac{{E_{j}}^{2}\left( \frac{w}{\eta_{j}} \right)}{\left( \frac{E_{j}}{\eta_{j}} \right)^{2}+w^{2}}$ | (69) |
| tan(ς) = E_l_/ E_s_ | (70) |

**Experiment DMA**

Dynamical Mechanical Analyser, or DMA, is a machine that mechanically deforms a material sample and measures its response, as a function of temperature, time or frequency. A multi-frequency test was performed on a single cantilever setup using a TA Instruments QDMA 800 machine to determine the variation in storage and loss moduli with excitation frequency. Various models were then fitted to the data, and the seven-parameter Weichert model was found to be most suitable within the specified error limits.

**Model Extraction**

The material has principal axes inclined at 0°, 45°, and 90° to the fibre, as shown in Fig. 31; thus, the DMA test is performed at Ψ = 0°, 45°, and 90°. Here, 1 and 2 denote the directions along and perpendicular to the fibre, Ψ denotes the angle made by the fibre axes (1 and 2) with the x axis

| 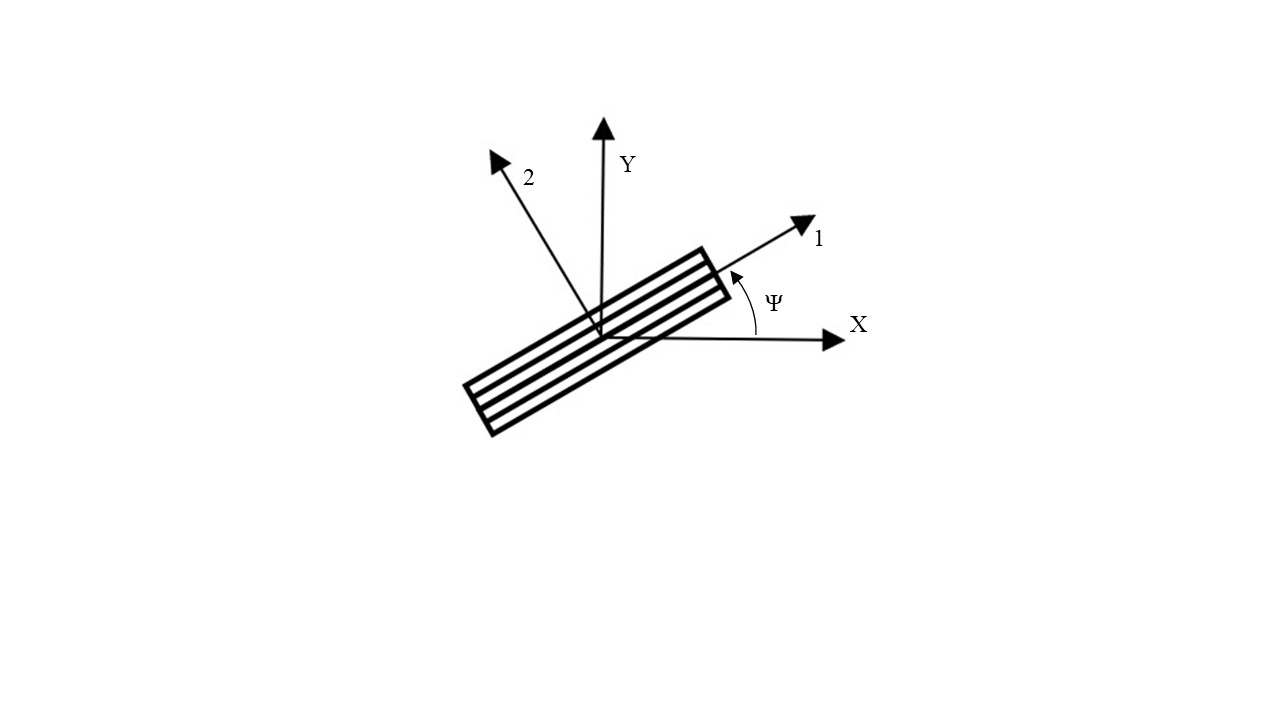 |
| --- |
| **Fig. 31 The fibre axes (1 and 2) w.r.t the material axes (X and Y)** |

To extract the parameters E_∞_, E_j_ and η_j_ (j = 1 to 3, in this case) for various models, a non-linear optimisation technique based on the Generalised Reduced Gradient (GRG) method is used. The flowchart below explains the method for finding the parameters.

Let E_se_ , E_le_ , tan(ς)_e_ and E_sm_ , E_sm_ , tan(ς)_m_  be the values obtained by experiment and model fitting, respectively. Then

Objective function:

Z = Min ( $\sum({{tan(\varsigma)}_{e}-{\tan\left( \varsigma\right)}_{m})}^{2}$)

Constraints:

Max. of Abs(100*(E_se_ - E_sm_)/ E_se_) $\leq$ a

Max. of Abs(100*(E_le_ – E_lm_)/ E_le_) $\leq$ b

Variables: E_∞_, E_j_ and η_j_ (j = 1 to 3) > 0

| 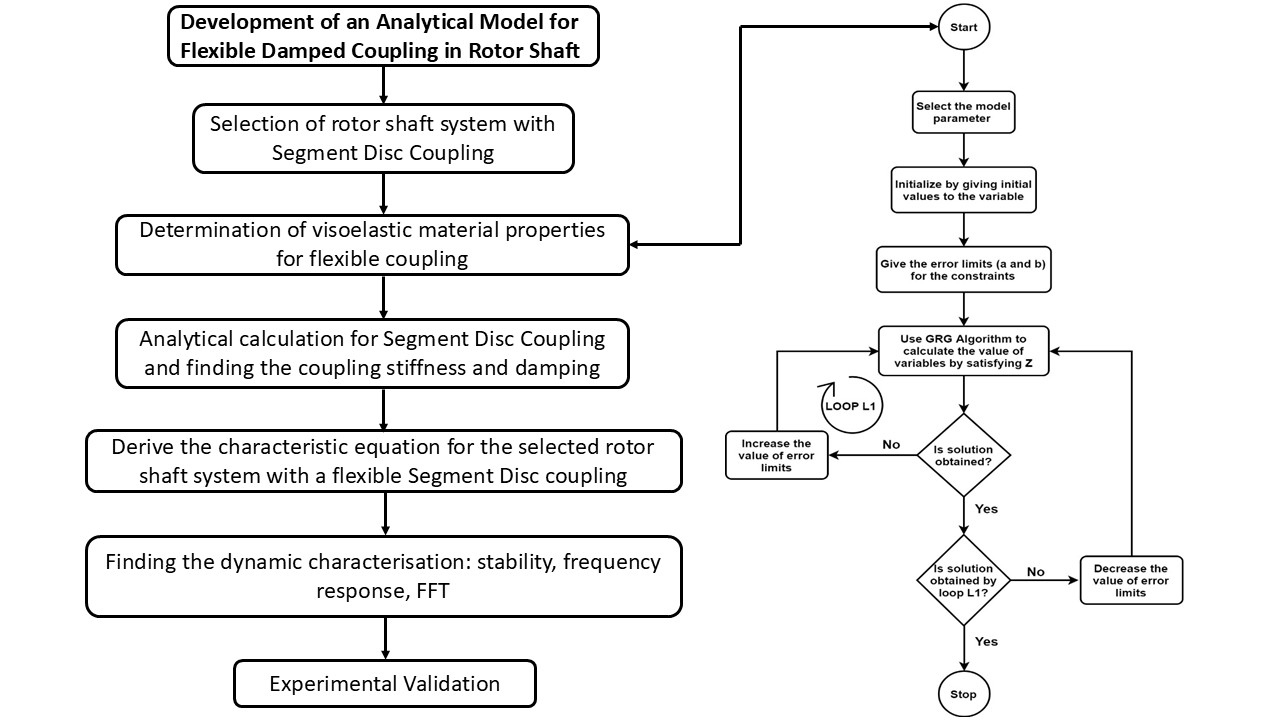 |
| --- |
| **Fig. 32 Flow chart explaining the methodology and model extraction procedure** |

Tables [6-8] show the extracted model in terms of the parameters E_∞_, E_j_ and η_j_ (j = 1 to 3).

**Table 6 Value of parameters for different models with fiber angle 0^0^**

| Model  Parameter | E_∞_ | E_1_ | E_2_ | E_3_ | $\eta_{1}$ | $\eta_{2}$ | $\eta_{3}$ |
| --- | --- | --- | --- | --- | --- | --- | --- |
| 3 | 14528.58 | 376.7628 | - | - | 1.5747 | - | - |
| 5 | 14399.33 | 263.2593 | 409.9384 | - | 22.6409 | 0.981096 | - |
| 7 | 14422.44 | 253.7447 | 104.4472 | 2.11×10^8^ | 29.1921 | 1.2104 | 0.59086 |

**Table 7 Value of parameters for different models with fiber angle 45^0^**

| Model  Parameter | E_∞_ | E_1_ | E_2_ | E_3_ | $\eta_{1}$ | $\eta_{2}$ | $\eta_{3}$ |
| --- | --- | --- | --- | --- | --- | --- | --- |
| 3 | 12518.09 | 332.9675 | - | - | 1.5091 | - | - |
| 5 | 12366.06 | 247.2794 | 354.9503 | - | 21.2314 | 0.8867 | - |
| 7 | 12356.26 | 229.6856 | 106.7878 | 431388 | 28.9879 | 1.3491 | 0.5215 |

**Table 8 Value of parameters for different models with fiber angle 90^0^**

| Model  Parameter | E_∞_ | E_1_ | E_2_ | E_3_ | $\eta_{1}$ | $\eta_{2}$ | $\eta_{3}$ |
| --- | --- | --- | --- | --- | --- | --- | --- |
| 3 | 12220.53 | 302.2567 | - | - | 1.1993 | - | - |
| 5 | 12082.3 | 216.8446 | 348.3851 | - | 18.5320 | 0.7310 | - |
| 7 | 12124.89 | 211.7214 | 72.7008 | 2.24×10^15^ | 25.0762 | 0.7948 | 0.4877 |

Fig.[33-35] show the experimental data with the model fit data for 3, 5 and 7 parameter models with angles Ψ = 0, 45 and 90 degrees. As the number of relaxation fields increases, the model becomes more accurate in predicting the material's behaviour. The maximum percentage error in Table 9 shows that the 7-parameter model most appropriately represents the material characteristic, with an error within the tolerance range of less than 10%.

| 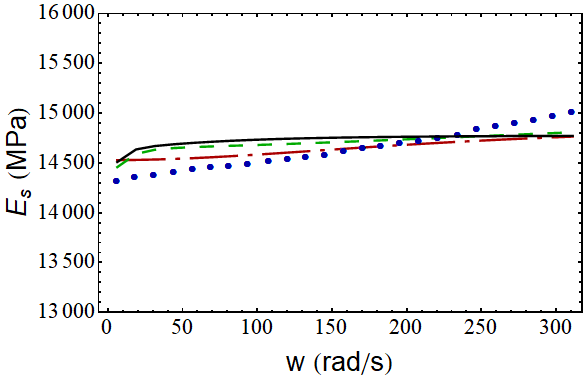 | 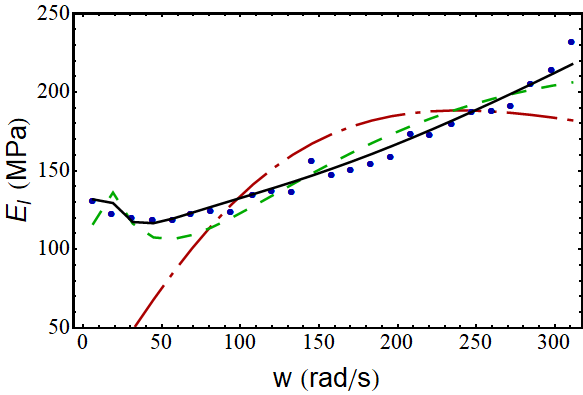 |
| --- | --- |
| **Fig. 33. Variation of storage (E_s_) and loss moduli (E_l_) with excitation frequency (w) for fiber angle (Ψ) = 0 degree. Key:**  **Experiment; 3 parameter;**  **5 parameter;** **7 parameter** | |
| 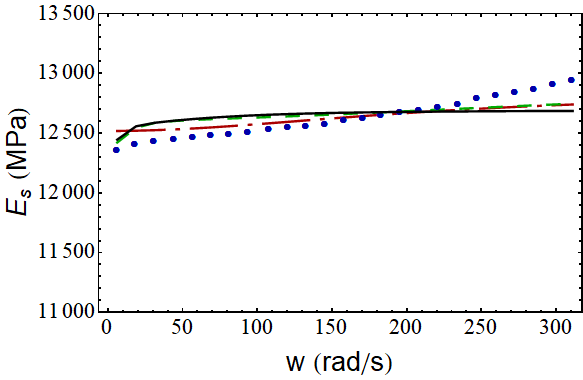 | 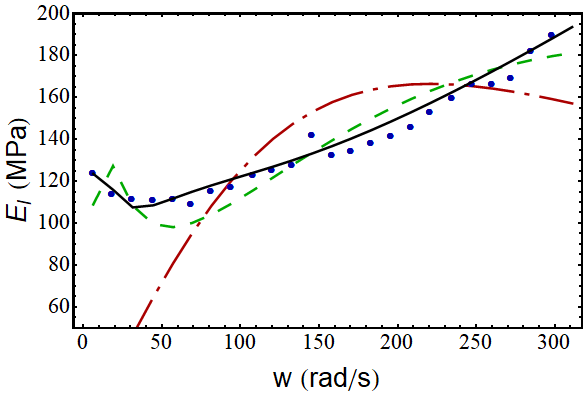 |
| **Fig. 34. Variation of storage (E_s_) and loss moduli (E_l_) with excitation frequency (w) for fiber angle (Ψ) = 45 degree.** | |
| 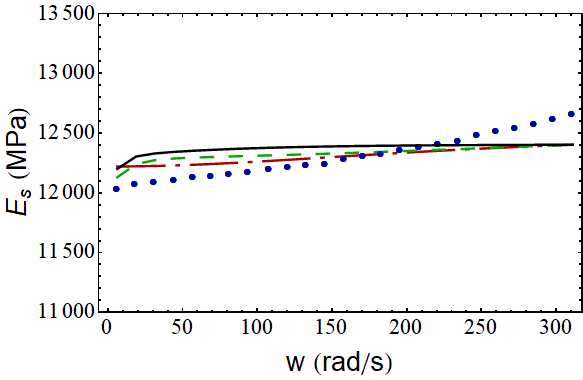 | 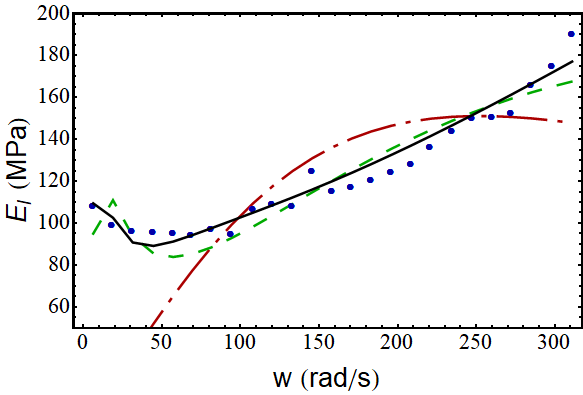 |
| **Fig. 35. Variation of storage (E_s_) and loss moduli (E_l_) with excitation frequency (w) for fiber angle (Ψ) = 90 degree.** | |

**Table 9: Maximum % error for various parametric models at different angles**

| Model | Modulus | 0 Degree (%) | 45 Degree (%) | 90 Degree (%) |
| --- | --- | --- | --- | --- |
| 3 Parameter | E_s_ | 1.69 | 1.58 | 1.99 |
|  | E_l_ | 92.43 | 92 | 93.02 |
| 5 Parameter | E_s_ | 1.38 | 1.53 | 2 |
|  | E_l_ | 11 | 12 | 12 |
| 7 Parameter | E_s_ | 2 | 2 | 2 |
|  | E_l_ | 6 | 6 | 7 |

The model of E* obtained in the above analysis is oriented at 0, 45, and 90 degrees with respect to the fibre. The method presented in Jones [25] is used to determine the value of E* at any angle Ψ relative to the principal axis.

| $\frac{1}{E_{x}^{*}}= \frac{1}{E_{0}^{*}}{cos(\Psi)}^{4}+\left[ \frac{1}{G_{12}}-\frac{2 \nu_{12}}{E_{0}^{*}} \right]{sin(\Psi)}^{2}{cos(\Psi)}^{2}+\frac{1}{E_{90}^{*}}{sin(\Psi)}^{4}$ | (71) |
| --- | --- |

Here, ν_12_, G_12_ are the Poisson’s ratio and shear moduli in the 1-2 plane, respectively. $E_{0}^{*}$, $E_{90}^{*}$_,_ $E_{45}^{*}$ and $E_{x}^{*}$ are the values of E* at 0 degrees, 45 degrees, 90 degrees and at any arbitrary angle $\Psi$ with 1 axis, respectively, and are represented as

| $E_{0}^{*}= E_{s0}+iE_{l0}$, $E_{90}^{*}= E_{s90}+iE_{l90}$ and $E_{x}^{*}= E_{\mathrm{sx}}+iE_{\mathrm{lx}}$ | (72) |
| --- | --- |
| $\left[ \frac{1}{G_{12}}-\frac{2 \nu_{12}}{E_{0}^{*}} \right]= \left[ \frac{4}{E_{45}^{*}}-\frac{1}{E_{0}^{*}}-\frac{1}{E_{90}^{*}} \right]$ | (73) |

Fig. 36 shows the variation of storage and loss moduli with angle $\Psi$. In the figures as w increases (outwards from the origin), both the storage and loss modulus increase, but the effect is more predominantly seen on the loss modulus.

| 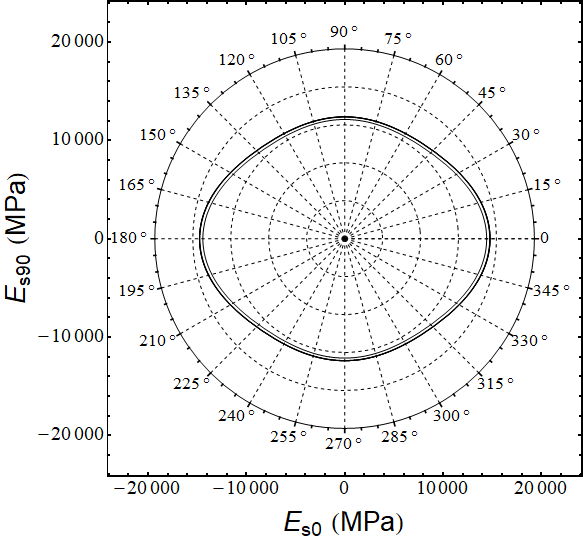 | 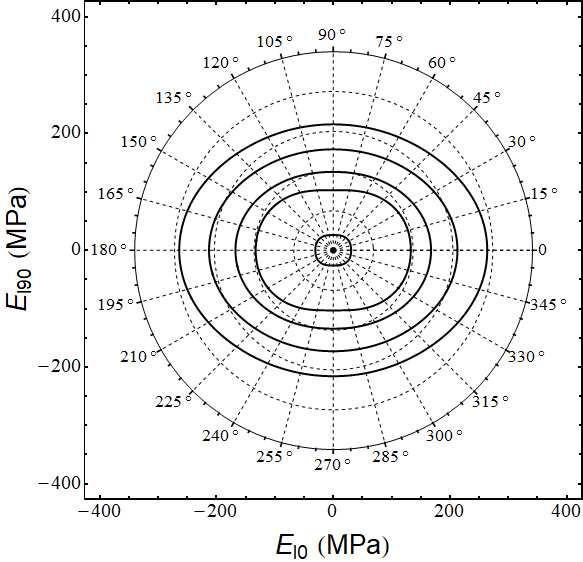 |
| --- | --- |
| **Fig. 36. Polar plot showing variation of Storage and Loss modulus with Ψ at different values of w (increases outwards from the origin)** | |

Fig. 37 a) shows the variation of loss modulus with increasing angular frequency. As the frequency of excitation increases the dampers present in the model (represented as combination of springs and dampers) began to freeze and thus loss modulus begins to drop after a particular frequency, the process continues till all the dampers get jammed, at this point loss modulus approaches zero and the resultant magnitude of the modulus approaches a constant value Fig. 37 b), i.e. it fully becomes solid, thus, material should be used below this frequency otherwise damping will not come into action.

| 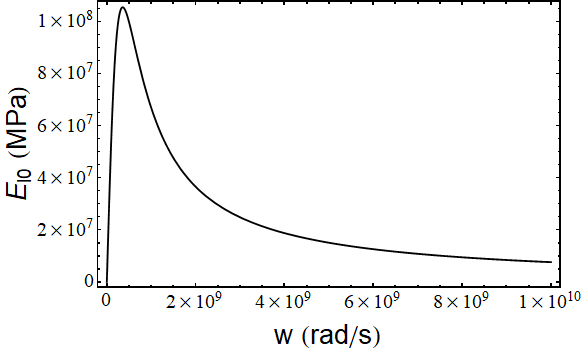  (a) | 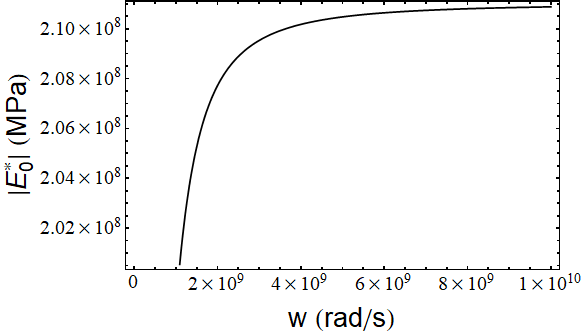  (b) |
| --- | --- |
| **Fig. 37 a) and b) Variation of loss modulus and total modulus with excitation frequency** | |

The coupling analysed in Section 8 had a link material with a fibre angle of 0 degrees; thus, the parameters obtained by model extraction for the 7-parameter model, Table 6, and the following Eq. 66.

| $E_{0}^{*}$(D) = $14422.44\text{ }+\frac{253.745D}{8.692\text{ }+D}+\frac{104.447D}{86.291\text{ }+D}+\frac{2.11\times{10}^{8}D}{3.571\times{10}^{8}+D}$ | (74) |
| --- | --- |

Ec used for the coupling in Appendix 1(a) is replaced by $E_{0}^{*}$(D) and the stiffness matrix is obtained
